# Prospects for artificial intelligence-enhanced electrocardiogram as a unified screening tool for cardiac and non-cardiac conditions: an explorative study in emergency care

**DOI:** 10.1093/ehjdh/ztae039

**Published:** 2024-05-12

**Authors:** Nils Strodthoff, Juan Miguel Lopez Alcaraz, Wilhelm Haverkamp

**Affiliations:** Carl von Ossietzky Universität Oldenburg, School VI Medicine and Health Services, Department of Health Services Research, Ammerländer Heerstr. 114-118, 26129 Oldenburg, Germany; Carl von Ossietzky Universität Oldenburg, School VI Medicine and Health Services, Department of Health Services Research, Ammerländer Heerstr. 114-118, 26129 Oldenburg, Germany; Charité Universitätsmedizin Berlin, Department of Cardiology and Metabolism, Clinic for Cardiology, Angiology, and Intensive Care Medicine, Berlin, Germany

**Keywords:** Artificial intelligence, ECG analysis, Deep learning, Diagnostic algorithms, Clinical decision support system

## Abstract

**Aims:**

Current deep learning algorithms for automatic ECG analysis have shown notable accuracy but are typically narrowly focused on singular diagnostic conditions. This exploratory study aims to investigate the capability of a single deep learning model to predict a diverse range of both cardiac and non-cardiac discharge diagnoses based on a single ECG collected in the emergency department.

**Methods and results:**

In this study, we assess the performance of a model trained to predict a broad spectrum of diagnoses. We find that the model can reliably predict 253 ICD codes (81 cardiac and 172 non-cardiac) in the sense of exceeding an AUROC score of 0.8 in a statistically significant manner.

**Conclusion:**

The model demonstrates proficiency in handling a wide array of cardiac and non-cardiac diagnostic scenarios, indicating its potential as a comprehensive screening tool for diverse medical encounters.

## Introduction

The electrocardiogram (ECG) holds a distinctive role as the primary tool for assessing a patient’s cardiac status, with over one-fourth of US emergency department visits involving an ECG.^1^ Presently, manual assessment predominates, with limited algorithmic support from rule-based ECG devices, known for their constraints.^2^ The emergence of deep learning has sparked interest in artificial intelligence (AI)-enhanced ECG interpretation, revolutionizing diagnostic perspectives.^3,4^ Numerous studies showcase deep learning’s accuracy in inferring diverse cardiac conditions, from myocardial infarction and comprehensive ECG statements^5,6^ to rhythm abnormalities.^7^ Remarkably, deep learning models demonstrate proficiency in inferring age, sex,^8^ ejection fraction,^9^ atrial fibrillation during sinus rhythm,^10^ anaemia,^11^ and non-cardiac conditions like diabetes^12^ and cirrhosis,^13^ challenging for human experts to discern from an ECG.

While notable AI-enabled ECG studies demonstrate impressive performance, a prevalent limitation is their narrow scope. Typically confined to binary prediction problems, these studies face challenges in defining appropriate control groups, potentially leading to an overestimation of algorithmic performance in real-world scenarios. Additionally, these studies are almost exclusively based on closed-source datasets, which hinder reproducibility and scientific progress. The availability of public ECG datasets has increased considerably,^14^ however, they typically lack clinical ground truth, limiting their utility for uncovering the diagnostic boundaries of the ECG. Finally, the emergence of specialized FDA-approved ECG algorithms raises questions about the feasibility of numerous isolated apps with limited scope, overlooking the intricate clinical reality of co-occurring diseases.

Existing works violate at least one of the points raised above. First of all, there is no comprehensive prediction algorithm beyond cardiovascular conditions based on raw ECGs as input. Even for cardiovascular diseases, binary conditions are the most common setup, with a few notable exceptions:^15^ cover different cardiovascular conditions, but restrict themselves to a rather coarse set of six conditions;^6^ achieve excellent results for the prediction of 66 cardiovascular conditions, which still fall short compared to the more than 150 cardiovascular conditions considered in this work, and base their work exclusively on a closed in-hospital dataset. Prediction models trained on public ECG datasets^14^ such as^5^ cover a somewhat extensive set of cardiovascular conditions, but lack clinical ground truth for more comprehensive investigations. Finally,^16^ is closest to our work as they also address discharge diagnosis prediction from the raw ECG, however, exclusively work on a closed in-hospital dataset and do not provide any external validation.

As already mentioned above, many, not exclusively, cardiac conditions leave traces in the ECG. However, apart from a small, selected number of conditions, this question has not been answered comprehensively, see Kashou *et al*.^17^ for a recent perspective. We envision that a deep learning-based ECG analysis algorithm trained on a comprehensive set of a general set of clinical diagnostic statements could provide patient profiles with detailed personalized risk scores (after appropriate calibration). Furthermore, learned features of such models could be used for deep phenotyping, in this case, obtained from supervised fine-tuning very much analogous to the widely used models pre-trained on ImageNet in computer vision, complementary to advances in self-supervised pre-training.^18^ These offer exciting prospects in terms of patient retrieval, also in combination with patient profiles from other modalities such as whole-genome sequencing or foundation models for medical imaging.

As a demonstration, we consider a subset of the full dataset under consideration of ECGs that were taken at the emergency department (ED) and investigate the feasibility of predicting ED diagnoses or (if available) hospital discharge diagnoses from them. The specific use case we have in mind is the triage in the ED, where electronic differential diagnostic support could lead to a significant reduction in diagnostic errors if integrated properly into the scope and the context of the ED triage process.^19^ The proposed model could be further supplemented by basic patient metadata such as patient demographics, chief complaints, and basic lab values to further improve model accuracy and robustness.

**Figure 1:**
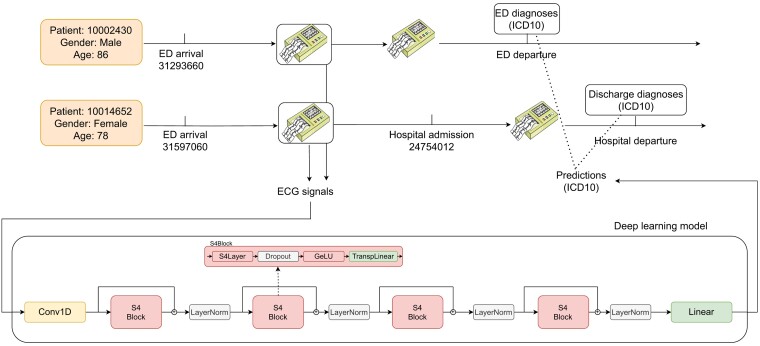
Schematic illustration depicting the proposed workflow, featuring two patient use cases. First, consider Patient 10002430, who does not undergo hospital admission, we try to infer the ED discharge diagnosis from the initial 10 s of ECG. This snippet is fed into our deep learning model, which outputs probabilities for each of the most reliably predictable ICD-10 codes (e.g. 439 codes with AUROC exceeding 0.8). Second, consider the arrival of Patient 10014652 to the emergency department (ED), where a variety of ECG recordings are obtained during both the ED stay and subsequent hospital admission. In this particular scenario, our objective is to predict the hospital discharge diagnosis, again based on the first 10 s of the recorded ECG. This approach allows us to leverage the most accurate clinical ground truth and to connect it to the first recorded ECG of the patient, which can provide valuable information for decisions at the ED.

In this exploratory study, we address the above limitations and explore the potential of deep learning in predicting a broad range of diagnoses, i.e. cardiac and non-cardiac discharge diagnoses from a single 12-lead ECG, with an application as a screening method in an ED setting in mind, based exclusively on publicly available data. We construct the MIMIC-IV-ECG-ICD-ED dataset from publicly available MIMIC-IV-ECG and MIMIC-IV data. State-of-the-art prediction models are trained and evaluated on this dataset, showing strong performance across over 1000 cardiac and non-cardiac conditions. We perform an initial external validation and compare our model’s performance to narrower-scope prediction models from the literature, discussing implications for ED triage. The course of the study is summarized in *Figure 1*.

## Methods

### Dataset construction and pre-processing

The proposed MIMIC-IV-ECG-ICD(-ED) dataset was created by linking signals from the MIMIC-IV-ECG^20^ dataset to clinical ground truth from the clinical MIMIC-IV dataset.^21^ This involved aligning ECG recording times with patient admission and discharge times, retrieval, and standardization of diagnostic codes (ICD-9-CM to ICD-10-CM), where hospital discharge diagnoses were given preference over ED diagnoses due to higher comprehensiveness and reliability. A detailed description of the dataset construction and pre-processing steps can be found in the Supplementary material.

### Prediction tasks and training procedures

The prediction task is a multi-label classification, where each patient’s discharge diagnosis is a set of ICD-10 statements, capturing clinical complexity comprehensively. We use all ECGs in the training set and minimize binary cross-entropy loss for multi-label prediction. Models are optimized using AdamW with a learning rate of 0.001 and weight decay of 0.001, trained for 20 epochs with a batch size of 32. We applied model selection based on the highest macro AUROC on the validation set to prevent overfitting, where the best-performing model was typically found around epoch 15. Prior research^5^ showed better performance by averaging predictions from shorter 2.5 s crops, despite models’ ability to appropriately handle long-range interactions.^22^ Therefore, we train on random 2.5 s crops and average predictions over four non-overlapping crops during testing. A single model training took ∼19 h on a single NVIDIA A30 GPU.

### Evaluation procedures

In contrast to the model training process, the test and validation sets only include the first ECG per ED/hospital stay per patient to prevent bias in model evaluation from patients with a large number of ECGs per stay. The primary evaluation metric is the macro average across all areas under the respective receiver operating curves (AUROC) (macro AUROC). To assess statistical uncertainty resulting from the finite size and specific composition of the test set, we employ empirical bootstrap on the test set with *n* = 1000 iterations. We report 95% confidence intervals for both macro AUROC and individual label AUROCs.

We primarily focus on the ED use case in our proposed dataset, enabling investigation into various conditions based on subsets used for training/evaluation and label sets, which may not necessarily coincide. To differentiate between them, we introduce the notation T(*A*2*B*)-E(*C*2*D*), where *A*, *C* ALL, ED, HOSP refers to the subset of ECGs used for training/evaluation and *B*, *D* ALL, ED, HOSP refers to the label sets used for training/evaluation. The main scenario is denoted as T(ED2ALL)-E(ED2ALL). In the Supplementary material, we compare this model with one trained on the most comprehensive dataset, T(ALL2ALL)-E(ALL2ALL), and explore various cross-evaluation scenarios, such as evaluating the comprehensive model on the ED subset (T(ALL2ALL)-E(ED2ALL)). Notably, the model trained on the most comprehensive dataset, T(ALL2ALL)-E(ED2ALL), achieved slightly lower performance compared to the specialized T(ED2ALL)-E(ED2ALL) model, with macro AUCs of 0.7691 and 0.7742, respectively, which was statistically significant. However, the specialized model performs considerably weaker across different evaluation scenarios. Detailed descriptions of these scenarios and extensive performance comparisons are provided in the Supplementary material.

## Results

### MIMIC-IV-ECG-ICD-ED dataset

**Figure 2:**
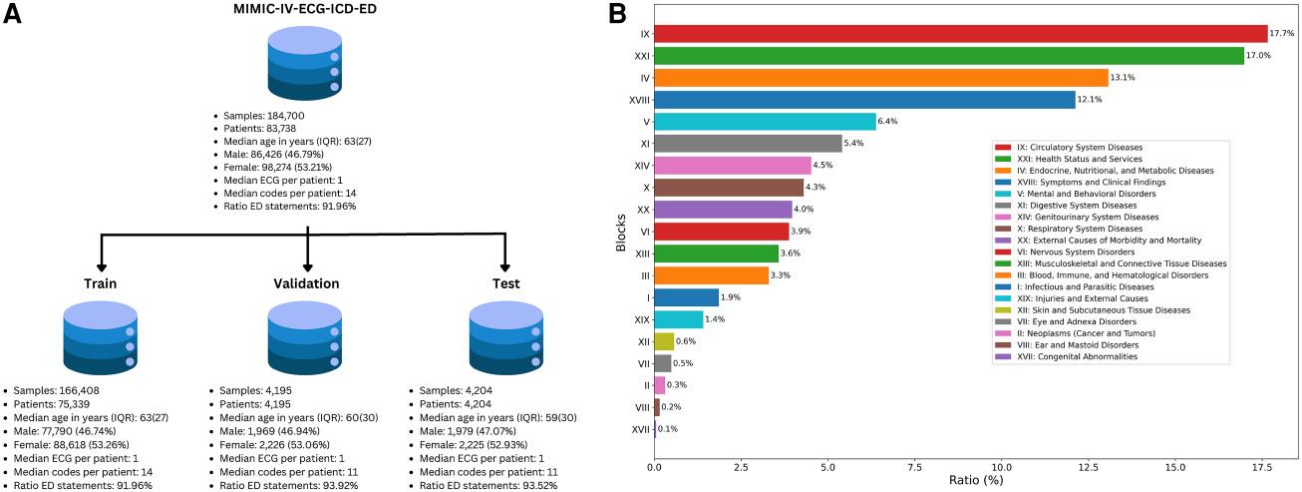
Schematic summary of the dataset composition and distribution of ICD codes across the dataset. (*A*) From the main MIMIC-IV-ECG-ICD-ED database of 184 700 samples across 83 738 patients, we utilize records of 75 339 patients for training, records of 4195 patients for model selection in the validation stage, and records of 4204 patients for testing. The median ECG records per patient is 1, however, the distribution is long-tailed with a maximum of ECG records per patient at 171. (*B*) represents the distribution of ICD codes according to chapters (all percentages as relative fractions compared to the dataset size), where chapter IX (Circulatory system diseases)is the most strongly represented chapter with 17.7%, closely followed by chapter XXI (Health system and status) with 17%, we present in Supplementary material the distribution of cardiac conditions within chapter IX (Circulatory system diseases categories).

We construct an ECG dataset with clinical labels, MIMIC-IV-ECG-ICD, as a subset of the MIMIC-IV-ECG dataset^20^ obtained by joining its records with hospital discharge diagnosis or ED diagnosis (in case the former is unavailable)from the MIMIC-IV dataset.^21^ In this work, we will only work with the subset of ECGs captured in the ED and refer to it as MIMIC-IV-ECG-ICD-ED dataset. In *Figure 2A*, we illustrate the dataset composition along with corresponding descriptive statistics, similarly, in *Figure 2B*, we summarize the ED subset in terms of the label distribution according to ICD-10 chapters.

### Model performance

We report classification results for a structured state-space sequence (S4) model,^23^ which outperformed state-of-the-art convolutional model, see the additional results in the Supplementary material, confirming earlier findings.^22^ In the first column in *Table 1*, we summarize performance according to ICD-10 chapters with most predictive chapters IX (Circulatory system diseases; AUROC 0.841) and X (Respiratory system diseases; AUROC 0.803). In 439 out of the 1079 considered ICD-10 codes, the performance exceeds an AUROC score of 0.8. The statistical uncertainty assessed via bootstrap confidence intervals is reasonably low with a median of 0.0506 (IQR 0.0373) across all codes. As a more conservative criterion for discovery, we consider only those codes for which the lower bound of the bootstrap confidence interval exceeds 0.8, i.e. conditions where the performance exceeds 0.8 in a statically significant manner. This singles out 253 ICD codes, 81 of which are cardiac and 172 of which are non-cardiac.

**Table 1:** Best-performing individual statements organized according to selected ICD chapters underscoring the breadth of accurately predictable statements

Block: block description. Block AUROC	Code: code AUROC. Code description	Code: code AUROC. Code description
IX: Circulatory System Diseases. 0.843	I210: 0.986. ST elevation (STEMI) myocardial infarction of anterior wall I447: 0.976. Left bundle-branch block, unspecified I451: 0.964. Other and unspecified right bundle-branch block I132: 0.948. Hypertensive heart and chronic kidney disease with heart failure and with stage 5 chronic kidney disease, or end stage renal disease I081: 0.944. Rheumatic disorders of both mitral and tricuspid valves I5043: 0.94. Acute on chronic combined systolic (congestive) and diastolic (congestive) heart failure I340: 0.913. Nonrheumatic mitral (valve) insufficiency I110: 0.906. Hypertensive heart disease with heart failure I851: 0.895. Secondary esophageal varices I46: 0.89. Cardiac arrest	I314: 0.979. Cardiac tamponade I481: 0.966. Persistent atrial fibrillation I255: 0.964. Ischemic cardiomyopathy I078: 0.948. Other rheumatic tricuspid valve diseases I2789: 0.943. Other specified pulmonary heart diseases I7025: 0.927. Atherosclerosis of native arteries of other extremities with ulceration I428: 0.906. Other cardiomyopathies I359: 0.897. Nonrheumatic aortic valve disorder, unspecified I200: 0.891. Unstable angina I120: 0.888. Hypertensive chronic kiey disease with stage 5 chronic kidney disease or end stage renal disease
X: Respiratory System Diseases. 0.804	J9621: 0.951. Acute and chronic respiratory failure with hypoxia J80: 0.905. Acute respiratory distress syndrome	J948: 0.925. Other specified pleural conditions J910: 0.904. Malignant pleural effusion
II: Neoplasms (Cancer and Tumors). 0.798	C7952: 0.933. Secondary malignant neoplasm of bone marrow C25: 0.884. Malignant neoplasm of pancreas	C925: 0.922. Acute myelomonocytic leukemia D469: 0.869. Myelodysplastic syndrome, unspecified
XXI: Health Status and Services. 0.793	Z998: 0.946. Dependence on other enabling machines and devices Z4502: 0.941. Encounter for adjustment and management of automatic implantable cardiac defibrillator	Z681: 0.944. Body mass index (BMI) 19.9 or less, adult Z950: 0.94. Presence of cardiac pacemaker
XII: Skin and Subcutaneous Tissue Diseases. 0.790	L891: 0.875. Pressure ulcer of back L0312: 0.851. Acute lymphangitis of other parts of limb	L9740: 0.87. Non-pressure chronic ulcer of unspecified heel and midfoot
IV: Endocrine, Nutritional, and Metabolic Diseases. 0.785 XIX: Injuries and External Causes. 0.777	E1129: 0.924. Type 2 diabetes mellitus with other diabetic kidney complication E103: 0.899. Type 1 diabetes mellitus with ophthalmic complications T8612: 0.944. Kidney transplant failure T380: 0.898. Poisoning by, adverse effect of and underdosing of glucocorticoids and synthetic analogues	E660: 0.907. Obesity due to excess calories E43: 0.886. Unspecified severe protein-calorie malnutrition T8285: 0.898. Stenosis due to cardiac and vascular prosthetic devices, implants and grafts
I: Infectious and Parasitic Diseases. 0.771	B9620: 0.862. Unspecified Escherichia coli [E. coli] as the cause of diseases classified elsewhere A40: 0.859. Streptococcal sepsis	A419: 0.86. Sepsis, unspecified organism
V: Mental and Behavioral Disorders. 0.766 XIV: Genitourinary System Diseases. 0.759	F1022: 0.894. Alcohol dependence with intoxication F4310: 0.864. Post-traumatic stress disorder, unspecified N186: 0.887. End stage renal disease N9982: 0.857. Postprocedural hemorrhage of a genitourinary system organ or structure following a procedure	F1721: 0.88. Nicotine dependence, cigarettes N08: 0.878. Glomerular disorders in diseases classified elsewhere N170: 0.852. Acute kidney failure with tubular necrosis
III: Blood, Immune, and Hematological Disorders. 0.755	D65: 0.933. Disseminated intravascular coagulation [defibrination syndrome]	D684: 0.878. Acquired coagulation factor deficiency
	D631: 0.857. Anemia in chronic kidney disease	
XI: Digestive System Diseases. 0.741 XVIII: Symptoms and Clinical Findings. 0.7162	K7031: 0.973. Alcoholic cirrhosis of liver with ascites K7290: 0.947. Hepatic failure, unspecified without coma R570: 0.931. Cardiogenic shock R18: 0.887. Ascites	K762: 0.948. Central hemorrhagic necrosis of liver K3189: 0.921. Other diseases of stomach and duodenum R64: 0.9. Cachexia R6521: 0.887. Severe sepsis with septic shock

The table shows the four best-performing individual statements per ICD chapter [20 for chapter IX (Circulatory system diseases)], where we show only AUROC point predictions above 0.85 where also the lower bound of the 95% bootstrap confidence interval exceeds 0.80. To showcase the breadth of reliably predictable statements, we list only the best-performing statement per three-digit ICD code. The complete list of AUROC scores for all 1076 ICD codes is provided in the Supplementary material as a summary of ICD codes at a three-digit level with AUROC scores above 0.9, 0.8, and below 0.7, respectively.

*Table 1* gives a comprehensive overview of conditions grouped by ICD chapters based on their predictability measured by AUROC scores. This list provides a broad view that can be seen as an exploratory approach towards many clinical directions. It contains a wide range set of cardiac conditions such as ST-elevation myocardial infarction, cardiac tamponade, left/right bundle branch block, persistent atrial fibrillation, or ischaemic cardiomyopathy (all with AUROC scores above 0.95). Notably, there were also non-cardiac codes with exceptionally high predictive performance such as *Escherichia coli*, leukaemia, type 2 diabetes, alcohol dependence, respiratory failure, alcoholic liver cirrhosis, ulcer, renal disease, cardiogenic shock, poisoning, traffic accidents, or the presence of assistance devices.

We analyse the results underlying *Table 1* from a complementary perspective by aggregating codes on the level of three-digit ICD codes and indicating also the *coverage*, i.e. the fraction of sub-statements (including the three-digit code itself)of a particular three-digit code that exceeds a pre-defined accuracy threshold (in this case chosen as AUROC > 0.9). A high coverage indicates that the model has acquired a good understanding of the particular condition including its corresponding differential diagnoses. Focusing on statements with a coverage of 75% or more, we see that the ECG is highly predictive for a wide range of cardiac conditions such as atrial fibrillation, hypertensive heart diseases, left bundle branch block, acute myocardial infarction, and heart failure. Notable non-cardiac conditions include pleural conditions, (alcoholic) liver diseases, traffic accidents, and assistant-device-related conditions.

We again assess groups of statements that can be reliably predicted from the ECG, however, this time at a slightly lower accuracy threshold of AUROC scores above 0.8. In the discussion below, we focus again on statements with a high (75%)coverage. Cardiovascular conditions from chapter IX most notably include chronic ischaemic heart diseases, atrial fibrillation, heart failure, hypertension, pulmonary heart diseases, acute myocardial infarction, valve disorders, cardiomyopathy, left bundle branch blocks, and other conduction disorders.

Our results imply that the ECG may have predictive capabilities across a very broad range of conditions including sepsis (I), neoplasms and leukaemia (II), anaemia and disseminated intravascular coagulation (III), diabetes type I, overweight and malnutrition, and partly also diabetes type II (IV), dementia and psychoactive drugs (V), Alzheimer’s and Parkinson’s (VI), respiratory failure, pleural effusion (X), liver diseases, alcoholic liver, stomach diseases, hepatic failure from (XI), ulcer (XII), gout (XIII), benign prostatic hyperplasia and chronic kidney diseases (XIV), heart valve malformation (XVII), systemic inflammation, shock (XVIII), different kinds of poisoning (XIX), traffic accidents (XX), and presence of cardiac implants or assistance devices, body mass index, absence of limb, and implanted device management (XXI). In parentheses, we always indicate the respective ICD chapter, which is also listed in full form in *Table 1*.

We acknowledge the importance of an external validation^24^ and therefore validate the performance of our model on the CODE test set^15^ across a set of six different cardiac conditions. The mapping between conditions and ICD-10 codes is non-trivial, so we picked the most typical ICD-10 code that aligns with it. Across all conditions in *Table 2*, the model exhibits an even stronger performance than on the internal test set despite a sizeable mismatch in label distribution across both datasets. As the most likely explanation, it is worth stressing that the prediction task on the internal is much more fine-grained requiring to differentiate between similar differential diagnoses instead of differentiating within a coarse set of only six conditions. The comparably weak performance on the bradycardia and tachycardia conditions might also be affected by missing annotations in the internal dataset, which are not ECG-specific annotations and only include the most important persistent conditions. There is presently no publicly available ECG dataset covering an ED patient cohort, which would qualify as an external validation dataset covering non-cardiac conditions.

**Table 2:** External validation on CODE test for diverse cardiac conditions

Statement	ICD-10 codes	Internal	External
1AVB	I440	0.908	0.942
AFIB	I4891	0.908	0.970
LBBB	I447	0.976	0.999
RBBB	I4510	0.964	0.989
SBRAD	R001	0.791	0.957
STACH	R000	0.849	0.985

We report AUROC scores for specified ICD-10 codes on the internal and on the external CODE test datasets.

1AVB, 1st degree AV block; AFIB, atrial fibrillation; LBBB, left bundle branch block; RBBB, right bundle branch block; SBRAD, sinus bradycardia; STACH, sinus tachycardia.

## Discussion

### Artificial intelligence-enhanced electrocardiogram as a unified screening tool

Our study demonstrates that using deep learning on a single 12-lead ECG effectively predicts both cardiac and non-cardiac conditions for discharge diagnoses, making it a valuable screening tool in an ED setting. In line with the explorative nature of this investigation, we see a large number of accurately predictable (also non-cardiac) statements as a strong hint at the diagnostic power of the AI-enhanced ECG, which remains to be validated in detailed follow-up studies. In addition to the external validation for common cardiac conditions, we further validate our model by comparing its performance to existing predictive models from the literature, which, however suffers from systematic uncertainties due to varying definitions of conditions and limited coverage of relevant pathologies in control groups. To address these challenges, the proposed MIMIC-IV-ECG-ICD dataset facilitates standardized comparisons with clinical ground truth, similar to PTB-XL,^14^ to accelerate progress in the field.

In addition to our external validation, we set our results into perspective by comparing them to landmark results from recent literature;^9^ report an AUROC score of 0.932 for the detection of a low ejection fraction of <35%, which is often associated with heart failure. To put this into perspective, for heart failure with reduced ejection fraction, we report an AUROC of 0.936. Another landmark paper assessed AF from sinus rhythm with an AUROC of 0.87. We compare this to the performance of our model on paroxysmal atrial fibrillation, which reaches an AUROC of 0.891. Turning to non-cardiac conditions,^25^ have developed predictive models for the detection of cirrhosis from ECGs with an AUROC of 0.908, we report 0.906 AUROC for cirrhosis detection as well as 0.973 for cirrhosis with ascites. Finally,^11^ demonstrated the feasibility of predicting anaemia from the ECG, reporting an AUROC score 0.923. We report AUROCs up to 0.857 for different sorts of anaemias.

While the above literature comparison might seem very selective, we present an extensive comparison of literature results in the Supplementary material. These findings highlight the competitiveness of the proposed model in both cardiac and non-cardiac conditions. A unique strength is its fine-grained predictions. While qualitative ECG changes are known for many non-cardiac conditions, our study provides the first quantitative evidence for their predictability. The alignment with literature results and correspondence with known qualitative ECG changes validate our approach, extending to conditions where predictability is reported for the first time.

Our model excels in predicting non-cardiac conditions, which may appear surprising given their apparent lack of correlation with the heart’s electrical function. While commonly thought to solely depict the heart’s electrical activity, the ECG is, in fact, intricately influenced by factors such as the autonomic nervous system, gender, hormones, age, weight, and extracardiac elements (thoracic configuration and impedance). Inflammatory or autoimmune diseases^26^ or even chest trauma may also cause ECG changes. Artificial intelligence’s recognition of certain profiles might be rooted in these extracardiac factors.

### Limitations

The proposed approach has several limitations. First, discharge diagnoses may include events unrelated to the patient’s condition captured by the ED ECG, and the coding process itself is prone to biases. The former could be addressed by incorporating temporal metadata, whereas the latter could be mitigated through the use of full-text discharge reports. However, it is worth stressing that the discharge diagnoses serve as a proxy for the clinical ground truth and hence represent a qualitative improvement over labels from expert annotations.

As a second limitation, we stress that our findings are associative and do not indicate causal relationships. Confounding factors like demographic variables, concurrent ailments, treatments, and nuanced medical history may obscure causal links. Clinical metadata, including chief complaint summaries, could uncover more intricate factors. Thirdly, in addressing co-occurring diseases as confounding factors, our approach is less susceptible to uncontrolled confounding effects compared to common binary approaches. Unlike methods requiring a distinct control group, our utilization of all ED ECGs encompasses a clinically relevant patient collective. Patients without the specific condition implicitly serve as a control group. For instance, a recent study^27^ noted confounding in detecting cirrhosis from an ECG due to ascites. Our model explicitly resolves this, achieving high AUROC scores for cirrhosis with and without ascites without creating specific control sets.

Lastly, despite efforts to control confounding factors, our approach may still be affected by comorbidities. We analyse correlations within the test set labels using Matthew’s correlation coefficients (MCCs) (detailed in Supplementary material). Among the 1076 most prevalent labels, the highest correlations exist between specific and parent statements, suggesting that the model might potentially not be able to capture the parent statement in its full breadth. Excluding parent statements reveals correlations across various labels, typically representing variations of codes for the same underlying condition or statements relating to a specific condition and a corresponding treatment (e.g. renal diseases and dialysis with MCC 0.84). Notably, the correlation between type 1 diabetes mellitus with opthalmological and neurological complications stands out with an MCC of 0.64. While our analysis does not strongly suggest significant confounding effects from co-occurring labels, they might have been exploited by the model in selected cases compromising the model’s ability for generalization and therefore warrants consideration in future investigations.

Using a similar methodology, we also investigated correlations of demographic subgroups (gender and subgroups of patients exceeding a certain age) and all considered diagnostic conditions in the test set. We identified mostly age-related correlations with certain diagnostic conditions (lipidaemia, atrial fibrillation, heart failure, atherosclerotic heart disease, dementia, chronic kidney diseases), all of them with moderate MCCs between 0.2 and 0.3, which could be at least exploited by the model, which could be analysed using concept-based XAI methods.^28^ However, it does not compromise the study’s aim of assessing the predictability of diagnostic statements from the ECG alone but rather provides supporting evidence for the inclusion of additional clinical metadata.

## Future research directions

As a promising direction for future work, leveraging explainable AI methods^28,29^ could enhance comprehension of disease-related ECG changes as insights from the model’s predictions. Similarly, while our model exclusively uses ECG data, future enhancements should prioritize the inclusion of additional inputs, such as demographic,^22^ chief complaint summaries,^30^ and basic lab values.

## Supplementary Material

ztae039_Supplementary_Data

## Data Availability

This study is based on the publicly available MIMIC-IV-ECG dataset^20^ (https://doi.org/10.13026/4nqg-sb35)in combination with clinical ground truth from the clinical MIMIC-IV dataset^21^ (https://doi.org/10.13026/6mm1-ek67). External validation was carried out based on the CODE test set.^15^ The source code underlying our investigations is available under https://github.com/AI4HealthUOL/ECG-MIMIC.
