# Supplementary material for: Prospects for artificial intelligence-enhanced electrocardiogram as a unified screening tool for cardiac and non-cardiac conditions: an explorative study in emergency care
Source: Eur Heart J Digit Health. 2024 May 12;5(4):454–60. doi: 10.1093/ehjdh/ztae039 (PMC11284007; doi:10.1093/ehjdh/ztae039)
Supplement: ztae039_Supplementary_Data [file ztae039_supplementary_data.zip › MIMIC_ECG.docx]

Prospects for AI-Enhanced ECG as a Unified Screening Tool for Cardiac and Non-Cardiac Conditions – An Explorative Study in Emergency Care

Nils Strodthoff^1,*^, Juan Miguel Lopez Alcaraz^1^, and Wilhelm Haverkamp^2^

1Carl von Ossietzky Universität Oldenburg, Oldenburg, Germany, {nils.strodthoff,juan.lopez.alcaraz}@uol.de

2Charité Universitätsmedizin Berlin, Berlin, Germany, [wilhelm.haverkamp@dhzc-charite.de](mailto:wilhelm.haverkamp@dhzc-charite.de)

*Corresponding author

April 11, 2024

Current deep learning algorithms designed for automatic ECG analysis have exhibited notable accuracy. However, akin to traditional electrocardiography, they tend to be narrowly focused and typically address a singular diagnostic condition. In this exploratory study, we specifically investigate the capability of a single model to predict a diverse range of both cardiac and non-cardiac discharge diagnoses based on a sole ECG collected in the emergency department. We find that 253, 81 cardiac and 172 non-cardiac, ICD codes can be reliably predicted in the sense of exceeding an AUROC score of 0.8 in a statistically significant manner. This underscores the model’s proficiency in handling a wide array of cardiac and non-cardiac diagnostic scenarios which demonstrates potential as a screening tool for diverse medical encounters.

**Keywords:** *Artificial intelligence, ECG analysis, Deep learning, Diagnostic algorithms, Clinical decision support system.*

# Introduction

The electrocardiogram (ECG) holds a distinctive role as the primary tool for assessing a patient’s cardiac status, with over one- fourth of U.S. emergency department visits involving an ECG [[1].](#_bookmark3) Presently, manual assessment predominates, with limited algorithmic support from rule-based ECG devices, known for their constraints [[2].](#_bookmark4) The emergence of deep learning, has sparked interest in AI-enhanced ECG interpretation, revolutionizing diagnostic perspectives [[3,](#_bookmark5) [4].](#_bookmark6) Numerous studies showcase deep learning’s accuracy in inferring diverse cardiac conditions, from myocardial infarction and comprehensive ECG statements [[5,](#_bookmark7) [6]](#_bookmark8) to rhythm abnormalities [[7].](#_bookmark9) Remarkably, deep learning models demonstrate proficiency in inferring age, sex [[8],](#_bookmark10) ejection fraction [[9],](#_bookmark11) atrial fibrillation during sinus rhythm [[10],](#_bookmark12) anemia [[11],](#_bookmark13) and non-cardiac conditions like diabetes [[12]](#_bookmark14) and cirrhosis [[13],](#_bookmark15) challenging for human experts to discern from an ECG.

While notable AI-enabled ECG studies demonstrate impressive performance, a prevalent limitation is their narrow scope. Typically confined to binary prediction problems, these studies face challenges in defining appropriate control groups, poten- tially leading to an overestimation of algorithmic performance in real-world scenarios. Additionally, these studies are almost exclusively based on closed-source datasets, which hinder reproducibility and scientific progress. The availability of public ECG datasets has increased considerably [[14],](#_bookmark16) however, they typically lack clinical ground truth, limiting their utility for un- covering the diagnostic boundaries of the ECG. Finally, the emergence of specialized FDA-approved ECG algorithms raises questions about the feasibility of numerous isolated apps with limited scope, overlooking the intricate clinical reality of co- occurring diseases.

Existing works violate at least one of the points raised above. First of all, there is no comprehensive prediction algorithm beyond cardiovascular conditions based on raw ECGs as input. Even for cardiovascular diseases, binary conditions are the most common setup, with a few notable exceptions. [[15]](#_bookmark17) cover different cardiovascular conditions, but restrict themselves to a rather coarse set of 6 conditions. [[6]](#_bookmark8) achieve excellent results for the prediction of 66 cardiovascular conditions, which still fall short compared to the more than 150 cardiovascular conditions considered in this work, and base their work exclusively on a closed in-hospital dataset. Prediction models trained on public ECG datasets [[14]](#_bookmark16) such as [[5]](#_bookmark7) cover a somewhat extensive set of cardiovascular conditions, but lack clinical ground truth for more comprehensive investigations. Finally, [[16]](#_bookmark18) is closest to our work as they also address discharge diagnosis prediction from the raw ECG, however, exclusively work on a closed in-hospital dataset and do not provide any external validation.

As already mentioned above, many, not exclusively, cardiac conditions leave traces in the ECG. However, apart from a small, selected number of conditions, this question has not been answered comprehensively, see [[17]](#_bookmark19) for a recent perspective. We envision that a deep-learning-based ECG analysis algorithm trained on a comprehensive set of a general set of clinical diagnostic


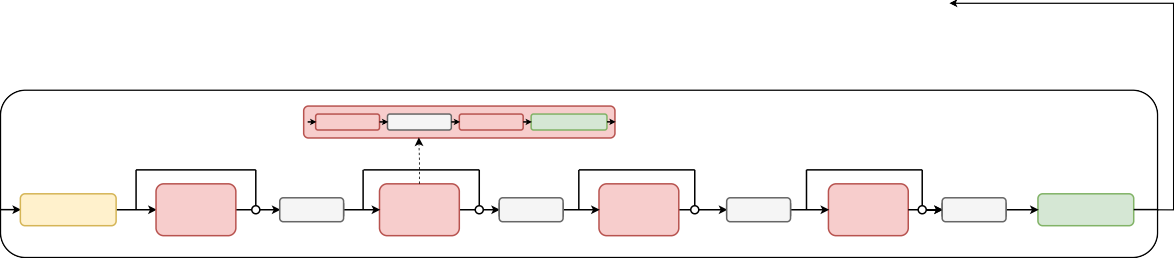

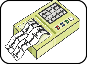

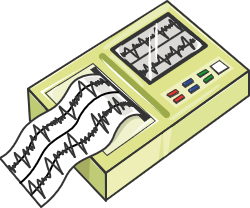

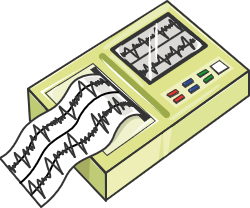

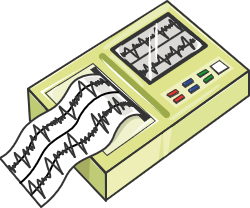


Patient: 10002430 Gender: Male

Age: 86

ED diagnoses (ICD10)

ED arrival 31293660

ED departure

Patient: 10014652 Gender: Female Age: 78

Discharge diagnoses (ICD10)

ED arrival 31597060

Hospital admission 24754012

Hospital departure

ECG signals

Predictions (ICD10)

Deep learning model

S4Block

S4Layer

Dropout

GeLU TranspLinear

Conv1D

S4

Block

+

LLaayyeerrNNoorrmm

S4

Block

+

LLaayyeerrNNoorrmm

S4

Block

+

LLaayyeerrNNoorrmm

S4

Block

+

LLaayyeerrNNoorrmm

Linear

Figure 1: Schematic illustration depicting the proposed workflow, featuring two patient use cases. First, consider Patient 10002430, who does not undergo hospital admission, we try to infer the ED discharge diagnosis from the initial 10s of ECG. This snippet is fed into our deep learning model, which outputs probabilities for each of the most reliably predictable ICD- 10 codes (e.g., 439 codes with AUROC exceeding 0.8). Second, consider the arrival of Patient 10014652 to the Emergency Department (ED), where a variety of ECG recordings are obtained during both the ED stay and subsequent hospital admission. In this particular scenario, our objective is to predict the hospital discharge diagnosis, again based on the first 10 seconds of the recorded ECG. This approach allows us to leverage the most accurate clinical ground truth and to connect it to the first recorded ECG of the patient, which can provide valuable information for decisions at the ED.

statements could provide patient profiles with detailed personalized risk scores (after appropriate calibration). Furthermore, learned features of such models could be used for deep phenotyping, in this case, obtained from supervised fine-tuning very much analogous to the widely used models pre-trained on ImageNet in computer vision, complementary to advances in self- supervised pre-training [[18].](#_bookmark20) These offer exciting prospects in terms of patient retrieval, also in combination with patient profiles from other modalities such as whole-genome sequencing or foundation models for medical imaging.

As a demonstration, we consider a subset of the full dataset under consideration of ECGs that were taken at the emergency department (ED) and investigate the feasibility of predicting ED diagnoses or (if available) hospital discharge diagnoses from them. The specific use case we have in mind is the triage in the ED, where electronic differential diagnostic support could lead to a significant reduction in diagnostic errors if integrated properly into the scope and the context of the ED triage process [[19].](#_bookmark21) The proposed model could be further supplemented by basic patient metadata such as patient demographics, chief complaints, and basic lab values to further improve model accuracy and robustness.

In this exploratory study, we address the above limitations and explore the potential of deep learning in predicting a broad range of diagnoses, i.e., cardiac and non-cardiac discharge diagnoses from a single 12-lead ECG, with an application as a screening method in an ED setting in mind, based exclusively on publicly available data. We construct the *MIMIC-IV-ECG- ICD-ED* dataset from publicly available MIMIC-IV-ECG and MIMIC-IV data. State-of-the-art prediction models are trained and evaluated on this dataset, showing strong performance across over 1000 cardiac and non-cardiac conditions. We perform an initial external validation and compare our model’s performance to narrower-scope prediction models from the literature, discussing implications for ED triage.

# Methods

## Dataset construction and preprocessing

The proposed *MIMIC-IV-ECG-ICD(-ED)* dataset was created by linking signals from the MIMIC-IV-ECG [[20]](#_bookmark22) dataset to clinical ground truth from the clinical MIMIC-IV dataset [[21].](#_bookmark23) This involved aligning ECG recording times with patient admission and discharge times, retrieval and standardization of diagnostic codes (ICD-9-CM to ICD-10-CM), where hospital discharge diagnoses was given preference over ED diagnoses due to higher comprehensiveness and reliability. A detailed description of the dataset construction and preprocessing steps can be found in the supplementary material.

## Prediction tasks and training procedures

The prediction task is a multi-label classification, where each patient’s discharge diagnosis is a set of ICD-10 statements, capturing clinical complexity comprehensively. We use all ECGs in the training set and minimize binary cross-entropy loss for multi-label prediction. Models are optimized using AdamW with a learning rate of 0.001 and weight decay of 0.001, trained for 20 epochs with a batch size of 32. We applied model selection based on the highest macro AUROC on the validation set to prevent overfitting, where the best-perfoming model was typically found around epoch 15. Prior research [[5]](#_bookmark7) showed better performance by averaging predictions from shorter 2.5s crops, despite models’ ability to appropriately handle long-range interactions [[22].](#_bookmark24) Therefore, we train on random 2.5s crops and average predictions over four non-overlapping crops during testing. A single model training took approximately 19 hours on a single NVIDIA A30 GPU.

## Evaluation procedures

In contrast to the model training process, the test and validation sets only include the first ECG per ED/hospital stay per patient to prevent bias in model evaluation from patients with a large number of ECGs per stay. The primary evaluation metric is the macro average across all areas under the respective receiver operating curves (AUROC) (macro AUROC). To assess statistical uncertainty resulting from the finite size and specific composition of the test set, we employ empirical bootstrap on the test set with *n* = 1000 iterations. We report 95% confidence intervals for both macro AUROC and individual label AUROCs.

We primarily focus on the ED use case in our proposed dataset, enabling investigation into various conditions based on sub- sets used for training/evaluation and label sets, which may not necessarily coincide. To differentiate between them, we introduce the notation T(*A*2*B*)-E(*C*2*D*), where *A, C* ALL*,* ED*,* HOSP refers to the subset of ECGs used for training/evaluation and *B, D* ALL*,* ED*,* HOSP refers to the label sets used for training/evaluation. The main scenario is denoted as T(ED2ALL)- E(ED2ALL). In the supplementary material, we compare this model with one trained on the most comprehensive dataset, T(ALL2ALL)-E(ALL2ALL), and explore various cross-evaluation scenarios, such as evaluating the comprehensive model on the ED subset (T(ALL2ALL)-E(ED2ALL)). Notably, the model trained on the most comprehensive dataset, T(ALL2ALL)- E(ED2ALL), achieved slightly lower performance compared to the specialized T(ED2ALL)-E(ED2ALL) model, with macro AUCs of 0.7691 and 0.7742, respectively, which was statistically significant. However, the specialized model performs con- siderably weaker across different evaluation scenarios. Detailed descriptions of these scenarios and extensive performance comparisons are provided in the supplementary material.

∈

∈

**MIMIC-IV-ECG-ICD-ED**


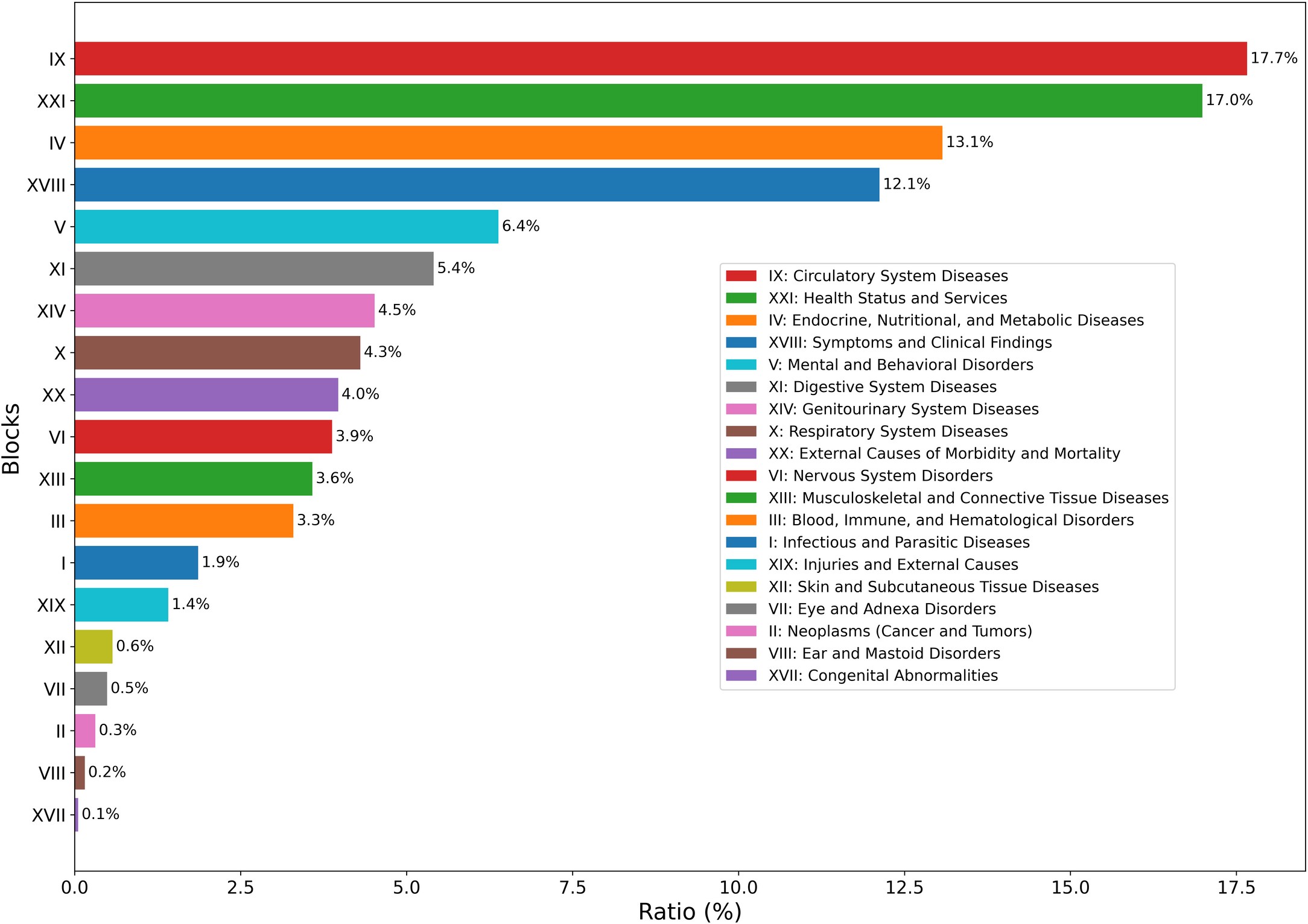


Samples: 184,700

Patients: 83,738


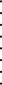


Median age in years (IQR): 63(27) Male: 86,426 (46.79%)

Female: 98,274 (53.21%)

Median ECG per patient: 1 Median codes per patient: 14 Ratio ED statements: 91.96%

**Train Validation Test**

Samples: 166,408


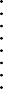


Patients: 75,339

Median age in years (IQR): 63(27) Male: 77,790 (46.74%)

Female: 88,618 (53.26%)

Median ECG per patient: 1 Median codes per patient: 14 Ratio ED statements: 91.96%

Samples: 4,195

Patients: 4,195


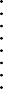


Median age in years (IQR): 60(30) Male: 1,969 (46.94%)

Female: 2,226 (53.06%)

Median ECG per patient: 1 Median codes per patient: 11 Ratio ED statements: 93.92%

**A**

Samples: 4,204

Patients: 4,204


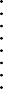


Median age in years (IQR): 59(30) Male: 1,979 (47.07%)

Female: 2,225 (52.93%)

Median ECG per patient: 1 Median codes per patient: 11 Ratio ED statements: 93.52%

**B**

Figure 2: Schematic summary of the dataset composition and distribution of ICD codes across the dataset. (A) From the main MIMIC-IV-ECG-ICD-ED database of 184,700 samples across 83,738 patients, we utilize records of 75,339 patients for training, records of 4,195 patients for model selection in the validation stage, and records of 4,204 patients for testing. The median ECG records per patient is 1, however, the distribution is long-tailed with a maximum of ECG records per patient at 171.

1. represents the distribution of ICD codes according to chapters (all percentages as relative fractions compared to the dataset size), where chapter IX (Circulatory system diseases) is the most strongly represented chapter with 17.7%, closely followed by chapter XXI (Health system and status) with 17%, we present in supplementary material the distribution of cardiac conditions within chapter IX (Circulatory system diseases categories).

# Results

## MIMIC-IV-ECG-ICD-ED Dataset

We construct an ECG dataset with clinical labels, *MIMIC-IV-ECG-ICD*, as a subset of the MIMIC-IV-ECG dataset [[20]](#_bookmark22) obtained by joining its records with hospital discharge diagnosis or ED diagnosis (in case the former is unavailable) from the MIMIC-IV dataset [[21].](#_bookmark23) In this work, we will only work with the subset of ECGs captured in the ED and refer to it as *MIMIC-IV-ECG- ICD-ED* dataset. In Fig. [2,](#_bookmark0) (A) we illustrate the dataset composition along with corresponding descriptive statistics, similarly, in (B) we summarize the ED subset in terms of the label distribution according to ICD-10 chapters.

## Model performance

We report classification results for a structured state-space sequence (S4) model [[23],](#_bookmark25) which outperformed state-of-the-art convolutional model, see the additional results in the supplementary material, confirming earlier findings [[22].](#_bookmark24) In the first column in Tab. [1,](#_bookmark1) we summarize performance according to ICD-10 chapters with most predictive chapters IX (Circulatory system diseases; AUROC 0.841) and X (Respiratory system diseases; AUROC 0.803). In 439 out of the 1079 considered ICD-10 codes the performance exceeds an AUROC score of 0.8. The statistical uncertainty assessed via bootstrap confidence intervals is reasonably low with a median of 0.0506 (IQR 0.0373) across all codes. As a more conservative criterion for discovery, we consider only those codes for which the lower bound of the bootstrap confidence interval exceeds 0.8, i.e., conditions where the performance exceeds 0.8 in a statically significant manner. This singles out 253 ICD codes, 81 of which are cardiac and 172 of which are non-cardiac.

Tab. [1](#_bookmark1) gives a comprehensive overview of conditions grouped by ICD chapters based on their predictability measured by AUROC scores. This list provides a broad view that can be seen as an exploratory approach toward many clinical directions. It contains a wide range set of cardiac conditions such as ST-elevation myocardial infarction, cardiac tamponade, left/right bundle branch block, persistent atrial fibrillation, or ischemic cardiomyopathy (all with AUROC scores above 0.95). Notably, there were also non-cardiac codes with exceptionally high predictive performance such as E. coli, leukemia, type 2 diabetes, alcohol dependence, respiratory failure, alcoholic liver cirrhosis, ulcer, renal disease, cardiogenic shock, poisoning, traffic accidents, or the presence of assistance devices.

We analyze the results underlying Tab. [1](#_bookmark1) from a complementary perspective by aggregating codes on the level of 3-digit ICD codes and indicating also the *coverage*, i.e., the fraction of sub-statements (including the 3-digit code itself) of a particular 3-digit code that exceeds a predefined accuracy threshold (in this case chosen as AUROC > 0.9). A high coverage indicates that the model has acquired a good understanding of the particular condition including its corresponding differential diagnoses. Focusing on statements with a coverage of 75% or more, we see that the ECG is highly predictive for a wide range of cardiac conditions such as atrial fibrillation, hypertensive heart diseases, left bundle branch block, acute myocardial infarction, and heart failure. Notable non-cardiac conditions include pleural conditions, (alcoholic) liver diseases, traffic accidents, and assistant- device-related conditions.

We again assess groups of statements that can be reliably predicted from the ECG, however, this time at a slightly lower accuracy threshold of AUROC scores above 0.8. In the discussion below we focus again on statements with a high (75%) coverage. Cardiovascular conditions from Chapter IX most notably include chronic ischemic heart diseases, atrial fibrillation, heart failure, hypertension, pulmonary heart diseases, acute myocardial infarction, valve disorders, cardiomyopathy, left bundle branch blocks, and other conduction disorders.

Our results imply that the ECG may have predictive capabilities across a very broad range of conditions including sepsis (I), neoplasms and leukemia (II), anemia and disseminated intravascular coagulation (III), diabetes type I, overweight and malnutrition, and partly also diabetes type II (IV), dementia and psychoactive drugs (V), Alzheimer’s and Parkinson’s (VI), respiratory failure, pleural effusion (X), liver diseases, alcoholic liver, stomach diseases, hepatic failure from (XI), ulcer (XII), gout (XIII), benign prostatic hyperplasia and chronic kidney diseases (XIV), heart valve malformation (XVII), systemic inflam- mation, shock (XVIII), different kinds of poisoning (XIX), traffic accidents (XX), presence of cardiac implants or assistance devices, body mass index, absence of limb, and implanted device management (XXI). In parentheses, we always indicate the respective ICD chapter, which is also listed in full form in Tab. [1.](#_bookmark1)

We acknowledge the importance of an external validation [[24]](#_bookmark26) and therefore validate the performance of our model on the CODE test set [[15]](#_bookmark17) across a set of six different cardiac conditions. The mapping between conditions and ICD-10 codes is non-trivial, so we picked the most typical ICD-10 code that aligns with it. Across all conditions in Tab. [2,](#_bookmark2) the model exhibits an even stronger performance than on the internal test setdespite a sizeable mismatch in label distribution across both datasets. As the most likely explanation, it is worth stressing that the prediction task on the internal is much more finegrained requiring to differentiate between similar differential diagnoses instead of differentiating within a coarse set of only 6 conditions. The comparably weak performance on the bradycardia and tachycardia conditions might also be affected by missing annotations in the internal dataset, which are not ECG-specific annotations and only include the most important persistent conditions. There

Block: Block description. Block AUROC Code: Code AUROC. Code description Code: Code AUROC. Code description

I210: 0.986. ST elevation (STEMI) myocardial infarction of anterior wall

I314: 0.979. Cardiac tamponade

I447: 0.976. Left bundle-branch block, unspecified I481: 0.966. Persistent atrial fibrillation I451: 0.964. Other and unspecified right bundle-branch block I255: 0.964. Ischemic cardiomyopathy

I132: 0.948. Hypertensive heart and chronic kidney disease with heart failure and with stage 5 chronic kidney disease, or end stage renal dis- ease

I078: 0.948. Other rheumatic tricuspid valve diseases

IX: Circulatory System Diseases. 0.843 I081: 0.944. Rheumatic disorders of both mitral and tricuspid valves I2789: 0.943. Other specified pulmonary heart diseases

I5043: 0.94. Acute on chronic combined systolic (congestive) and di- astolic (congestive) heart failure

I7025: 0.927. Atherosclerosis of native arteries of other extremities with ulceration

I340: 0.913. Nonrheumatic mitral (valve) insufficiency I428: 0.906. Other cardiomyopathies

I110: 0.906. Hypertensive heart disease with heart failure I359: 0.897. Nonrheumatic aortic valve disorder, unspecified I851: 0.895. Secondary esophageal varices I200: 0.891. Unstable angina

I46: 0.89. Cardiac arrest I120: 0.888. Hypertensive chronic kidney disease with stage 5 chronic kidney disease or end stage renal disease

X: Respiratory System Diseases. 0.804

J9621: 0.951. Acute and chronic respiratory failure with hypoxia J948: 0.925. Other specified pleural conditions J80: 0.905. Acute respiratory distress syndrome J910: 0.904. Malignant pleural effusion

C7952: 0.933. Secondary malignant neoplasm of bone marrow C925: 0.922. Acute myelomonocytic leukemia

II: Neoplasms (Cancer and Tumors). 0.798

C25: 0.884. Malignant neoplasm of pancreas D469: 0.869. Myelodysplastic syndrome, unspecified Z998: 0.946. Dependence on other enabling machines and devices Z681: 0.944. Body mass index (BMI) 19.9 or less, adult

XXI: Health Status and Services. 0.793

Z4502: 0.941. Encounter for adjustment and management of automatic

implantable cardiac defibrillator

Z950: 0.94. Presence of cardiac pacemaker

XII: Skin and Subcutaneous Tissue Diseases. 0.790

L891: 0.875. Pressure ulcer of back L9740: 0.87. Non-pressure chronic ulcer of unspecified heel and mid- foot

L0312: 0.851. Acute lymphangitis of other parts of limb

IV: Endocrine, Nutritional, and Metabolic Diseases. 0.785

E1129: 0.924. Type 2 diabetes mellitus with other diabetic kidney com- plication

E660: 0.907. Obesity due to excess calories

E103: 0.899. Type 1 diabetes mellitus with ophthalmic complications E43: 0.886. Unspecified severe protein-calorie malnutrition

T8612: 0.944. Kidney transplant failure T8285: 0.898. Stenosis due to cardiac and vascular prosthetic devices,

XIX: Injuries and External Causes. 0.777

I: Infectious and Parasitic Diseases. 0.771

T380: 0.898. Poisoning by, adverse effect of and underdosing of gluco- corticoids and synthetic analogues

B9620: 0.862. Unspecified Escherichia coli [E. coli] as the cause of diseases classified elsewhere

A40: 0.859. Streptococcal sepsis

implants and grafts

A419: 0.86. Sepsis, unspecified organism

F1022: 0.894. Alcohol dependence with intoxication F1721: 0.88. Nicotine dependence, cigarettes F4310: 0.864. Post-traumatic stress disorder, unspecified

V: Mental and Behavioral Disorders. 0.766

N186: 0.887. End stage renal disease N08: 0.878. Glomerular disorders in diseases classified elsewhere

XIV: Genitourinary System Diseases. 0.759

III: Blood, Immune, and Hematological Disorders. 0.755

N9982: 0.857. Postprocedural hemorrhage of a genitourinary system organ or structure following a procedure

D65: 0.933. Disseminated intravascular coagulation [defibrination syn- drome]

D631: 0.857. Anemia in chronic kidney disease

N170: 0.852. Acute kidney failure with tubular necrosis D684: 0.878. Acquired coagulation factor deficiency

K7031: 0.973. Alcoholic cirrhosis of liver with ascites K762: 0.948. Central hemorrhagic necrosis of liver K7290: 0.947. Hepatic failure, unspecified without coma K3189: 0.921. Other diseases of stomach and duodenum

XI: Digestive System Diseases. 0.741

R570: 0.931. Cardiogenic shock R64: 0.9. Cachexia

R18: 0.887. Ascites R6521: 0.887. Severe sepsis with septic shock

XVIII: Symptoms and Clinical Findings. 0.7162

Table 1: Best-performing individual statements organized according to selected ICD chapters underscoring the breadth of accurately predictable statements. The table shows the four best-performing individual statements per ICD chapter (20 for chapter IX (Circulatory system diseases)), where we show only AUROC point predictions above 0.85 where also the lower bound of the 95% bootstrap confidence interval exceeds 0.80. To showcase the breadth of reliably predictable statements, we list only the best-performing statement per 3-digit ICD code. The complete list of AUROC scores for all 1076 ICD codes is provided in the supplementary material as a summary of ICD codes at a 3-digit level with AUROC scores above 0.9, 0.8, and below 0.7 respectively.

| Statement | ICD-10 codes | Internal | External |
| --- | --- | --- | --- |
| 1AVB | I440 | 0.908 | 0.942 |
| AFIB | I4891 | 0.908 | 0.970 |
| LBBB | I447 | 0.976 | 0.999 |
| RBBB | I4510 | 0.964 | 0.989 |
| SBRAD | R001 | 0.791 | 0.957 |
| STACH | R000 | 0.849 | 0.985 |

Table 2: External validation on CODE-test for diverse cardiac conditions (1AVB: 1st degree AV block, AFIB: atrial fibriallation, LBBB: left bundle branch block, RBBB: right bundle branch block, SBRAD: sinus bradycardia, STACH: sinus tachycardia). We report AUROC scores for specified ICD-10 codes on the internal and on the external CODE-test dataset.

is presently no publicly available ECG dataset covering an ED patient cohort, which would qualify as an external validation dataset covering non-cardiac conditions.

# Discussion

## AI-enhanced ECG as a unified screening tool

Our study demonstrates that using deep learning on a single 12-lead ECG effectively predicts both cardiac and non-cardiac conditions for discharge diagnoses, making it a valuable screening tool in an Emergency Department (ED) setting. In line with the explorative nature of this investigation, we see a large number of accurately predictable (also non-cardiac) statements as a strong hint at the diagnostic power of the AI-enhanced ECG, which remains to be validated in detailed follow-up studies. In addition to the external validation for common cardiac conditions, we further validate our model by comparing its performance to existing predictive models from the literature, which, however suffers from systematic uncertainties due to varying definitions of conditions and limited coverage of relevant pathologies in control groups. To address these challenges, the proposed *MIMIC- IV-ECG-ICD* dataset facilitates standardized comparisons with clinical ground truth, similar to PTB-XL [[14],](#_bookmark16) to accelerate progress in the field.

In addition to our external validation, we set our results into perspective by comparing them to landmark results from recent literature. [[9]](#_bookmark11) report an AUROC score of 0.932 for the detection of a low ejection fraction of less than 35%, which is often associated with heart failure. To put this into perspective, for *heart failure with reduced ejection fraction* we report an AUROC of 0.936. Another landmark paper assessed AF from sinus rhythm with an AUROC of 0.87. We compare this to the performance of our model on *paroxysmal atrial fibrillation*, which reaches an AUROC of 0.891. Turning to non-cardiac conditions, [[25]](#_bookmark27) have developed predictive models for the detection of cirrhosis from ECGs with an AUROC of 0.908, we report 0.906 AUROC for *cirrhosis* detection as well as 0.973 for *cirrhosis with ascites*. Finally, [[11]](#_bookmark13) demonstrated the feasibility of predicting *anemia* from the ECG, reporting an AUROC score 0.923. We report AUROCs up to 0.857 for different sorts of anemias.

While the above literature comparison might seem very selective, we present an extensive comparison of literature results in the supplementary material. These findings highlight the competitiveness of the proposed model in both cardiac and non-cardiac conditions. A unique strength is its fine-grained predictions. While qualitative ECG changes are known for many non-cardiac conditions, our study provides the first quantitative evidence for their predictability. The alignment with literature results and correspondence with known qualitative ECG changes validate our approach, extending to conditions where predictability is reported for the first time.

Our model excels in predicting non-cardiac conditions, which may appear surprising given their apparent lack of correlation with the heart’s electrical function. While commonly thought to solely depict the heart’s electrical activity, the ECG is, in fact, intricately influenced by factors such as the autonomic nervous system, gender, hormones, age, weight, and extracardiac elements (thoracic configuration and impedance). Inflammatory or autoimmune diseases [[26]](#_bookmark28) or even chest trauma may also cause ECG changes. AI’s recognition of certain profiles might be rooted in these extracardiac factors.

## Limitations

The proposed approach has several limitations. First, discharge diagnoses may include events unrelated to the patient’s condi- tion captured by the ED ECG, and the coding process itself is prone to biases. The former could be addressed by incorporating temporal metadata, whereas the latter could be mitigated through the use of full-text discharge reports. However, it is worth stressing that the discharge diagnoses serve as a proxy for the clinical ground truth and hence represent a qualitative improve- ment over labels from expert annotations.

As a second limitation, we stress that our findings are associative and do not indicate causal relationships. Confounding factors like demographic variables, concurrent ailments, treatments, and nuanced medical history may obscure causal links. Clinical metadata, including chief complaint summaries, could uncover more intricate factors. Thirdly, in addressing co- occurring diseases as confounding factors, our approach is less susceptible to uncontrolled confounding effects compared to common binary approaches. Unlike methods requiring a distinct control group, our utilization of all ED ECGs encompasses a clinically relevant patient collective. Patients without the specific condition implicitly serve as a control group. For instance, a recent study [[27]](#_bookmark29) noted confounding in detecting cirrhosis from an ECG due to ascites. Our model explicitly resolves this, achieving high AUROC scores for cirrhosis with and without ascites without creating specific control sets.

Lastly, despite efforts to control confounding factors, our approach may still be affected by comorbidities. We analyze correlations within the test set labels using Matthew’s correlation coefficients (MCCs) (detailed in supplementary material). Among the 1076 most prevalent labels, the highest correlations exist between specific and parent statements, suggesting that the model might potentially not be able to capture the parent statement in its full breadth. Excluding parent statements reveals correlations across various labels, typically representing variations of codes for the same underlying condition or statements relating to a specific condition and a corresponding treatment (e.g. renal diseases and dialysis with MCC 0.84). Notably, the

correlation between type 1 diabetes mellitus with opthalmological and neurological complications stands out with an MCC of 0.64). While our analysis does not strongly suggest significant confounding effects from co-occurring labels, they might have been exploited by the model in selected cases compromizing the model’s ability for generalization and therefore warrants consideration in future investigations.

Using a similar methodology, we also investigated correlations of demographic subgroups (gender and subgroups of patients exceeding a certain age) and all considered diagnostic conditions in the test set. We identified mostly age-related correlations with certain diagnostic conditions (lipidemia, atrial fibrillation, heart failure, atherosclerotic heart disease, dementia, chronic kidney diseases) all of them with moderate MCCs between 0.2 and 0.3, which could be at least exploited by the model, which could be analyzed using concept-based XAI methods [[28].](#_bookmark30) However, it does not compromise the study’s aim of assessing the predictability of diagnostic statements from the ECG alone but rather provides supporting evidence for the inclusion of additional clinical metadata.

## Future research directions

As a promising direction for future work, leveraging explainable AI methods [[28,](#_bookmark30) [29]](#_bookmark31) could enhance comprehension of disease- related ECG changes as insights from the model’s predictions. Similarly, while our model exclusively uses ECG data, future enhancements should prioritize the inclusion of additional inputs, such as demographic [[22],](#_bookmark24) chief complaint summaries [[30],](#_bookmark32) and basic lab values.

**Contributors** NS and WH conceptualized the study. NS produced the first code prototype. JMLA. carried out the full experiments. NS and JMLA summarized the outputs and produced display items. All authors interpreted the results. NS and JMLA wrote the first draft and all authors revised it. All authors approved the submitted version.

# Data sharing

This study is based on the publicly available MIMIC-IV-ECG dataset [[20](](#_bookmark22)<https://doi.org/10.13026/4nqg-sb35>) in combination with clinical ground truth from the clinical MIMIC-IV dataset [[21](](#_bookmark23)[https://doi.org/10.13026/](https://doi.org/10.13026/6mm1-ek67) [6mm1-ek67](https://doi.org/10.13026/6mm1-ek67)). External validation was carried out based on the CODE test set [[15].](#_bookmark17) The source code underlying our in- vestigations is available under <https://github.com/AI4HealthUOL/ECG-MIMIC>.

# Declaration of Interests

The authors declare no competing interests.

# References

- 1. CDC, “National Hospital Ambulatory Medical Care Survey: 2021 National Summary Tables,” tech. rep., Centers for Disease Control and Prevention, 2021.
  2. J. Schläpfer and H. J. Wellens, “Computer-interpreted electrocardiograms: benefits and limitations,” *Journal of the Amer- ican College of Cardiology*, vol. 70, no. 9, pp. 1183–1192, 2017.
  3. E. J. Topol, “What’s lurking in your electrocardiogram?,” *The Lancet*, vol. 397, no. 10276, p. 785, 2021.
  4. K. C. Siontis, P. A. Noseworthy, Z. I. Attia, and P. A. Friedman, “Artificial intelligence-enhanced electrocardiography in cardiovascular disease management,” *Nature Reviews Cardiology*, vol. 18, pp. 465–478, Feb. 2021.
  5. N. Strodthoff, P. Wagner, T. Schaeffter, and W. Samek, “Deep learning for ECG analysis: Benchmarks and insights from PTB-XL,” *IEEE Journal of Biomedical and Health Informatics*, pp. 1–1, 2020.
  6. A. H. Kashou, W.-Y. Ko, Z. I. Attia, M. S. Cohen, P. A. Friedman, and P. A. Noseworthy, “A comprehensive artificial intelligence–enabled electrocardiogram interpretation program,” *Cardiovascular Digital Health Journal*, vol. 1, pp. 62– 70, Sept. 2020.
  7. A. Y. Hannun, P. Rajpurkar, M. Haghpanahi, G. H. Tison, C. Bourn, M. P. Turakhia, and A. Y. Ng, “Cardiologist-level arrhythmia detection and classification in ambulatory electrocardiograms using a deep neural network,” *Nature Medicine*, vol. 25, pp. 65–69, Jan. 2019.
  8. Z. I. Attia, P. A. Friedman, P. A. Noseworthy, F. Lopez-Jimenez, D. J. Ladewig, G. Satam, P. A. Pellikka, T. M. Munger,

S. J. Asirvatham, C. G. Scott, R. E. Carter, and S. Kapa, “Age and sex estimation using artificial intelligence from standard 12-lead ECGs,” *Circulation: Arrhythmia and Electrophysiology*, vol. 12, Sept. 2019.

- 1. Z. I. Attia, S. Kapa, F. Lopez-Jimenez, P. M. McKie, D. J. Ladewig, G. Satam, P. A. Pellikka, M. Enriquez-Sarano,

P. A. Noseworthy, T. M. Munger, S. J. Asirvatham, C. G. Scott, R. E. Carter, and P. A. Friedman, “Screening for cardiac contractile dysfunction using an artificial intelligence–enabled electrocardiogram,” *Nature Medicine*, vol. 25, pp. 70–74, Jan. 2019.

- 1. Z. I. Attia, P. A. Noseworthy, F. Lopez-Jimenez, S. J. Asirvatham, A. J. Deshmukh, B. J. Gersh, R. E. Carter, X. Yao, A. A. Rabinstein, B. J. Erickson, *et al.*, “An artificial intelligence-enabled ecg algorithm for the identification of patients with atrial fibrillation during sinus rhythm: a retrospective analysis of outcome prediction,” *The Lancet*, vol. 394, no. 10201, pp. 861–867, 2019.
  2. J.-M. Kwon, Y. Cho, K.-H. Jeon, S. Cho, K.-H. Kim, S. D. Baek, S. Jeung, J. Park, and B.-H. Oh, “A deep learning algorithm to detect anaemia with ECGs: a retrospective, multicentre study,” *The Lancet Digital Health*, vol. 2, pp. e358– e367, July 2020.
  3. A. R. Kulkarni, A. A. Patel, K. V. Pipal, S. G. Jaiswal, M. T. Jaisinghani, V. Thulkar, L. Gajbhiye, P. Gondane, A. B. Patel,

M. Mamtani, *et al.*, “Machine-learning algorithm to non-invasively detect diabetes and pre-diabetes from electrocardio- gram,” *BMJ Innovations*, vol. 9, no. 1, 2023.

- 1. J. C. Ahn, Z. I. Attia, P. Rattan, A. F. Mullan, S. Buryska, A. M. Allen, P. S. Kamath, P. A. Friedman, V. H. Shah,

P. A. Noseworthy, and D. A. Simonetto, “Development of the AI-cirrhosis-ECG score: An electrocardiogram-based deep learning model in cirrhosis,” *American Journal of Gastroenterology*, vol. 117, pp. 424–432, Dec. 2021.

- 1. P. Wagner, N. Strodthoff, R.-D. Bousseljot, D. Kreiseler, F. I. Lunze, W. Samek, and T. Schaeffter, “PTB-XL, a large publicly available electrocardiography dataset,” *Scientific Data*, vol. 7, no. 1, p. 154, 2020.
  2. A. H. Ribeiro, M. H. Ribeiro, G. M. M. Paixão, D. M. Oliveira, P. R. Gomes, J. A. Canazart, M. P. S. Ferreira, C. R. Andersson, P. W. Macfarlane, W. Meira, T. B. Schön, and A. L. P. Ribeiro, “Automatic diagnosis of the 12-lead ECG using a deep neural network,” *Nature Communications*, vol. 11, Apr. 2020.
  3. W. Sun, S. V. Kalmady, A. Salimi, N. Sepehrvand, E. Ly, A. Hindle, R. Greiner, and P. Kaul, “Ecg for high-throughput screening of multiple diseases: Proof-of-concept using multi-diagnosis deep learning from population-based datasets,” in *Medical Imaging meets NeurIPS workshop*, 2021.
  4. A. H. Kashou, D. A. Adedinsewo, K. C. Siontis, and P. A. Noseworthy, “Artificial intelligence-enabled ecg: Physiologic and pathophysiologic insights and implications,” *Comprehensive Physiology*, pp. 3417–3424, June 2022.
  5. T. Mehari and N. Strodthoff, “Self-supervised representation learning from 12-lead ECG data,” *Computers in Biology and Medicine*, vol. 141, p. 105114, 2022.
  6. M. Sibbald, B. Abdulla, A. Keuhl, G. Norman, S. Monteiro, and J. Sherbino, “Electronic diagnostic support in emergency physician triage: Qualitative study with thematic analysis of interviews,” *JMIR Human Factors*, vol. 9, p. e39234, Sept. 2022.
  7. B. Gow, T. Pollard, L. A. Nathanson, A. Johnson, B. Moody, C. Fernandes, N. Greenbaum, J. W. Waks, P. Eslami,

T. Carbonati, A. Chaudhari, E. Herbst, D. Moukheiber, S. Berkowitz, R. Mark, and S. Horng, “Mimic-iv-ecg: Diagnostic electrocardiogram matched subset,” 2023.

- 1. A. E. W. Johnson, L. Bulgarelli, L. Shen, A. Gayles, A. Shammout, S. Horng, T. J. Pollard, S. Hao, B. Moody, B. Gow,

L. wei H. Lehman, L. A. Celi, and R. G. Mark, “MIMIC-IV, a freely accessible electronic health record dataset,” *Scientific Data*, vol. 10, Jan. 2023.

- 1. T. Mehari and N. Strodthoff, “Towards quantitative precision for ECG analysis: Leveraging state space models, self- supervision and patient metadata,” *IEEE Journal of Biomedical and Health Informatics*, pp. 1–9, 2023.
  2. A. Gu, K. Goel, and C. Re, “Efficiently modeling long sequences with structured state spaces,” in *International Conference on Learning Representations*, 2021.
  3. A. M. Chekroud, M. Hawrilenko, H. Loho, J. Bondar, R. Gueorguieva, A. Hasan, J. Kambeitz, P. R. Corlett, N. Kout- souleris, H. M. Krumholz, J. H. Krystal, and M. Paulus, “Illusory generalizability of clinical prediction models,” *Science*, vol. 383, p. 164âA˘ S¸ 167, Jan. 2024.
  4. J. C. Ahn, Z. I. Attia, P. Rattan, A. F. Mullan, S. Buryska, A. M. Allen, P. S. Kamath, P. A. Friedman, V. H. Shah, P. A. Noseworthy, *et al.*, “Development of the ai-cirrhosis-ecg (ace) score: an electrocardiogram-based deep learning model in cirrhosis,” *The American journal of gastroenterology*, vol. 117, no. 3, p. 424, 2022.
  5. P. L. Capecchi, F. Laghi-Pasini, N. El-Sherif, Y. Qu, M. Boutjdir, and P. E. Lazzerini, “Autoimmune and inflammatory k+ channelopathies in cardiac arrhythmias: Clinical evidence and molecular mechanisms,” *Heart Rhythm*, vol. 16, pp. 1273– 1280, Aug. 2019.
  6. D. Ouyang, “The uncanny and unreasonable performance of ai in medical imaging,” in *Causality in Medical Image Computing (MICCAI 2023 tutorial)*, 2023. https://sites.google.com/view/causemic.
  7. P. Wagner, T. Mehari, W. Haverkamp, and N. Strodthoff, “Explaining deep learning for ecg analysis: Building blocks for auditing and knowledge discovery,” *arXiv preprint arXiv:2305.17043*, 2023.
  8. J. Vielhaben, S. Blücher, and N. Strodthoff, “Multi-dimensional concept discovery (MCD): A unifying framework with completeness guarantees,” *Transactions on Machine Learning Research*, 2023.
  9. H.-Y. Zhou, Y. Yu, C. Wang, S. Zhang, Y. Gao, J. Pan, J. Shao, G. Lu, K. Zhang, and W. Li, “A transformer-based representation-learning model with unified processing of multimodal input for clinical diagnostics,” *Nature Biomedical Engineering*, vol. 7, pp. 743–755, June 2023.
