# Supplementary material for: Prospects for artificial intelligence-enhanced electrocardiogram as a unified screening tool for cardiac and non-cardiac conditions: an explorative study in emergency care
Source: Eur Heart J Digit Health. 2024 May 12;5(4):454–60. doi: 10.1093/ehjdh/ztae039 (PMC11284007; doi:10.1093/ehjdh/ztae039)
Supplement: ztae039_Supplementary_Data [file ztae039_supplementary_data.zip › Supplement.pdf]

Supplementary material for "AI-Enhanced ECG: A Unified Screening Tool for Cardiac and Non-Cardiac Conditions – An Explorative Study in Emergency Care"

Dataset construction and preprocessing

To link samples from the MIMIC-IV-ECG dataset [20] to clinical ground truth from the clinical MIMIC-IV dataset [21], we identified ECGs taken in the ED or hospital by comparing recording times to patient admission, discharge, and potential death times. This process yielded ED stay IDs (*stayid*) for ED-captured ECGs and hospital admission IDs (*hadmid*) for ECGs taken in the hospital or for ED ECGs of patients subsequently admitted. ED stay IDs enabled retrieval of ED discharge diagnoses (max. 9 ICD-9-CM or ICD-10-CM codes), while hospital admission IDs link samples to hospital discharge diagnoses (max. 39 ICD-9-CM or ICD-10-CM codes).

We used the Python package *icd-mappings* to convert ICD-9-CM to ICD-10-CM codes, establishing a common vocabulary. All codes were truncated to a consistent five-digit format (e.g., I48.92 for unspecified atrial flutter), retaining entries with fewer digits and removing trailing 'X' placeholder characters. Superclasses up to a three-digit level were included to ensure consistent mapping (e.g., I48.9 and I48 for unspecified atrial fibrillation and atrial flutter, respectively). ECG samples were resampled to 100Hz [22], with missing signal values linearly interpolated and infrequent missing values at sequence boundaries replaced with zero. Signals were clipped to a maximum amplitude of 3 mV. Apart from resampling, handling missing values, and clipping, no further preprocessing was applied to the raw ECG signals. The MIMIC-IV-ECG dataset patients were randomly assigned to twenty folds: 18 for training, 1 for validation and model selection, and 1 for testing. Corresponding fold assignments are included in the code repository for reproducibility.

The linking process defines three label sets: ED discharge diagnoses, hospital discharge diagnoses (from ED ECGs of subsequently admitted patients or from hospital ECGs), and a combination of both. In cases of both present, the hospital diagnosis is prioritized for its comprehensiveness (max. 39 vs. 9 ED discharge diagnoses) and higher diagnostic precision. Although training generally occurs on the combined set, various subsets with different label sets are evaluated. A negligible fraction of ECGs with an empty set of discharge diagnoses is discarded, as examination of corresponding discharge reports showed many lacked ICD annotations. The final label sets are selected by discarding codes occurring less than 2000 times in the dataset, resulting in a label set comprising 1076 3- to 5-digit ICD-10-CM codes for training.

Literature comparison: overview

Most accurately predictable cardiac codes We begin our discussion on cardiovascular conditions with high-performing statements from Tab. 1. Interestingly, several of these statements have primarily been inferred from imaging modalities rather than directly from the ECG. [31] predict *cardiac tamponade* after AF diagnoses based on tabular features (patient metadata, previous diagnoses, laboratory values) with 0.84 AUROC. We predict it from a single ECG alone with a significantly higher AUROC score of 0.979. Prior work on *ischemic cardiomyopathy* was typically based on imaging modalities such as echocardiography [32, 33] with AUROC scores of 0.934. We report a performance of 0.964 for ischemic cardiomyopathy from the ECG alone. [34] detected *cardiac arrest* (within 24h hours) from ECG signals with a performance of 0.913 AUROC. We report an AUROC of 0.890 without any constraints on the time frame. The assessment of *pulmonary heart diseases* is challenging as the typical signs may be insensitive and often appear late in the disease. We report an AUROC score of 0.943.

Accurately predictable cardiac codes The list of accurately predictable cardiac conditions is extensive and we provide selected insights prioritized by prevalence. For a more comprehensive discussion, please refer to the supplementary material. In the domain of *chronic ischemic heart diseases*, [35] used imaging noninvasive tests, and biomarker panels for the detection of atherosclerotic. We report AUROCs of 0.827 for chronic ischemic heart disease as a broad category, as well as 0.825, and 0.903 for atherosclerotic with native coronary artery without angina pectoris and with angina pectoris, respectively. [36] review deep-learning methods for *atrial fibrillation* and flutter detection by ECGs, which includes the work of [37], with AUROCs ranging from 0.82 to 0.99. Deep learning models [5] on open source datasets reach AUROC scores of 0.982. This should be compared to a score of 0.966 for persistent atrial fibrillation. In a more fine-grained setting [38] reported an AUROC of 0.876 for *paroxysmal atrial fibrillation*, in comparison to 0.891 from our model. [39] used temporal, spectral, and complex HRV dynamics to detect *heart failure* with an AUROC of 0.97. [40] used demographics and ECG features as predictive variables with an AUROC of 0.843. For a fair comparison, we report an AUROC of 0.906 as a broad category heart failure, however, stress that our model provides very accurate differential diagnoses for acute/chronic systolic/diastolic heart failure. [9] report an AUROC score of 0.932 for the detection of a low ejection fraction of less than 35%, which is often associated with heart failure. To put this into perspective, for heart failure with reduced ejection fraction (I50.20) we report an AUROC of 0.936. [41] review ECG changes related to *hypertensive heart disease*. We report 0.814 AUROC for HCKD detection, and 0.929 for a combination of hypertensive heart and chronic kidney disease. [42] predicted hypertensive crisis (HC) from 24-hour ambulatory blood pressure monitoring, while [43] predicted hospital readmissions for HC specifically for pregnancy with AUROC of 0.85. We report 0.881 for HC detection. [44] predicted pulmonary hypertension from ECGs and transthoracic echocardiography with

an AUROC of 0.88, while [45] reported 0.89, we report 0.890 for its detection. [46] differentiated ST-elevation *myocardial infarction* (STEMI) from controls based on ECG data with 0.995 AUROC, whereas [47] achieved 0.991. We report a global performance of 0.870, however, we achieved higher performance on more fine-grained settings such as STEMI of the anterior wall and inferior wall of 0.986 and 0.950 respectively. Similarly, [47] achieved 0.832 for non-ST elevation (NSTEMI) whereas we achieved 0.847.

Predictability of non-cardiac conditions We focus on non-cardiac conditions from selected chapters. A more detailed discussion across all chapters is in the supplementary material. Direct comparisons are limited due to scarce literature results. One such example is [48], who report an AUROC score of 0.901 for detecting *sepsis*. Here we achieve an AUROC score of 0.86 across different kinds of sepsis in a more comprehensive patient collective. [11] demonstrated the feasibility of predicting *anemia* from the ECG, reporting an AUROC score 0.923. We report AUROCs up to 0.857 for different sorts of anemias based on diverse causes. [25] have developed predictive models for the detection of cirrhosis from ECGs with an AUROC of 0.908, we report 0.906 AUROC for cirrhosis detection as well as 0.973 for cirrhosis with ascites. Also *chronic kidney disease* has been shown to lead to ECG changes in patients with hypoalbuminemia [49] and has been previously detected from ECGs with an AUROC of 0.767 [50], we report an AUROC of 0.834. Another work predicted mitral valve prolapse from ECGs either congenital or not with an AUROC of 0.80 [51]. We report an AUROC of 0.863 for *congenital malformations of aortic and mitral valves*.

Literature comparison: Cardiovascular conditions

We proceed by discussing ICD codes at the 3-digit level that can be predicted accurately:

I25: Chronic ischemic heart diseases In the domain of *chronic ischemic heart diseases*, [35] used imaging noninvasive tests, and biomarker panels for the detection of atherosclerotic heart disease. [52] uses a neuro-fuzzy binary classification setting for ischemic heart disease detection using ECG signals, specifically features extracted from the QRST zone. Remarkably, the study achieves a high recognition score, however, they do not use a performance metric that allows for a direct comparison to our results. We report AUROCs of 0.827 for chronic ischemic heart disease as a broad category, as well as 0.825, and 0.903 for atherosclerotic heart disease with native coronary artery without angina pectoris and with angina pectoris, respectively.

I48: Atrial fibrillation [36] review diverse deep-learning methods for *atrial fibrillation* and flutter detection by ECGs, which includes the work of [37], with AUROCs ranging from 0.82 to 0.99. Deep learning models [5] on open source datasets such as PTB-XL [14] reach AUROC scores of 0.982. This should be compared to a score of 0.966 for persistent atrial fibrillation obtained from our model. Furthermore, [53] used a deep learning model to predict atrial fibrillation from 24-h Holter recording, as well as [54] based on RR-intervals, however, neither of them reports AUROC scores. In a more fine-grained setting, [38] reported an AUROC of 0.876 for *paroxysmal atrial fibrillation*, in comparison to 0.891 from our model. Further, we report 0.966 for persistent AF, and 0.948 for chronic AF.

I50: Heart failure [39] used temporal, spectral, and complex HRV dynamics to detect *heart failure* with an AUROC of 0.97. [40] used demographics and ECG features as predictive variables and reached an AUROC of 0.843. We report an AUROC of 0.906 as a broad category heart failure, however, stress that our model provides very accurate differential diagnoses for acute/chronic and systolic/diastolic heart failure. [9] report an AUROC score of 0.932 for the detection of a low ejection fraction <35%, which is often associated with heart failure. For heart failure with reduced ejection fraction (I50.20) we report an AUROC of 0.936.

I11-I16: Hypertensive heart diseases [41] review ECG changes related to *hypertensive heart disease*. [55] investigated strategies to improve care for patients with hypertensive chronic kidney disease of certain (HCKD) ethnicity. We report 0.814 AUROC for HCKD detection, and 0.929 for a combination of hypertensive heart and chronic kidney disease. [42] predicted hypertensive crisis (HC) from 24-hour ambulatory blood pressure monitoring, while [43] predicted hospital readmissions for HC specifically within pregnancy with AUROC of 0.85. We report 0.881 for HC detection. [44] predicted pulmonary hypertension from ECGs and transthoracic echocardiography with an AUROC of 0.88, while [45] reported 0.89, we report 0.890 for its detection.

I21 Acute myocardial infarction [46] differentiated ST-elevation *myocardial infarction* (STEMI) from controls based on ECG data with 0.995 AUROC, whereas [47] achieved 0.991. We report a global performance of 0.870, however, we achieved higher performance on more fine-grained settings such as STEMI of the anterior wall and inferior wall of 0.986 and 0.950 respectively. Similarly, [47] achieved 0.832 for non-ST elevation (NSTEMI) whereas we achieved 0.847. Finally, myocardial infarction type 2 represents a more challenging diagnosis to differentiate and also increases the risk of post-discharge mortality [56], for which we achieved 0.936. Further, [57] also investigated myocardial infarction however with different evaluation metrics.

I07,I08,I34,I35: Valve disorders With respect to *valve disorders*, [58] achieved 0.88 for mitral valve prolapse, whereas [59] reported 0.77. The proposed model reaches an AUROC score of 0.913. [58] achieved 0.89 on aortic stenosis, where [60] correlates LVH with aortic stenosis with an AUROC of 0.92. We achieve 0.879. Further, we achieve 0.948 for tricuspid valve diseases, and significant performance on disorders on multiple valves such as mitral and artic with 0.863, as well as mitral and tricuspid with 0.944.

I44,I45: Conduction disorders The predictive power of the ECG for the prediction of *atrioventricular blocks* (AV) has been studied prominently in the literature [15], however, they only report global model performance which includes more than these conditions. [5] report 0.971, 0.860, and 0.995 for 1st, 2nd, and 3rd degree whereas we report 0.908, 0.953, and 0.957 respectively. Similarly, other blocks such as left and right bundle-branch (LBBB) and (RBBB) have previously reported significant predictive performance in the literature, where [61] achieved 0.875 and [5] 0.998 for LBBB while we report 0.976. [62] achieved 0.93 and [5] 0.998 for RBBB while we achieved 0.964.
I65-I69: Cerebrovascular issues Imaging modalities are commonly used to characterize the anatomy of *cerebrovascular issues* such as carotid issues and justify open surgical interventions, see [63] for a review. From the ECG alone, we can detect carotid artery occlusion and stenosis with 0.836, cerebral atherosclerosis with 0.800, and sequels of cerebral infarction with 0.877, with fine-grained diagnosis after the infarction such as hemiplegia and hemiparesis with 0.873.

Literature comparison: Non-cardiovascular conditions

We structure the discussion of non-cardiovascular conditions by ICD-10 chapters:
I: Certain infectious and parasitic diseases [48] presented an algorithm to detect *sepsis* with a high predictive accuracy (AUROC 0.901). Here, we achieve an AUROC score of 0.86 across different kinds of sepsis in a more realistic patient collective. There are no literature results on the direct prediction of specific infectious diseases from the raw ECG (e.g. *E.coli* with AUROC 0.862).
II: Neoplasms Also the prediction of *neoplasms* has not been investigated directly from ECG data, even though isolated records on ECG changes due to the presence of lung cancer exist [64]. Similarly, [65] investigated the sub-clinical heart damage and ECG abnormalities due to the toxic chemotherapy effect, as well as [66] for melanoma patients, which might in fact also represent a confounding factor in our case. We demonstrate significant predictive performance on neoplasms, primarily in the bronchus and lungs 0.827, brain 0.827, prostate 0.802, pancreas 0.884, and bone marrow 0.933, as well as *leukemia* with 0.922.
III: Diseases of the blood and blood-forming organs and certain disorders involving the immune mechanism [11] demonstrated the feasibility of predicting *anemia* from the ECG, reporting an AUROC score 0.923, whereas we have AUROCs up to 0.857 for different sorts of anemias based on diverse causes. Nevertheless, we also report significant AUROCs for the disease of the blood and blood-forming organs such as 0.812 for *pancytopenia* and 0.933 for *disseminated intravascular coagulation*.
IV: Endocrine, nutritional and metabolic diseases *Diabetes* represents the dominant condition in the endocrine, nutritional, and metabolic chapter, which is known to impact the ECG [67, 68]. [12] developed an algorithm that was able to discriminate between no-diabetes, pre-diabetes and type 2 diabetes, but did not report AUROC scores. We report significant prediction accuracy both on diabetes type 1 and 2 with diverse secondary complications, e.g. type 2 with kidney complications 0.924 and neurological complications 0.880. Further, we also achieve high performance on sterol and serum electrolyte conditions such as *cholesterolemia*, *calcemias*, *kalemias* and *magnesemias*.
V: Mental and behavioural disorders . We report an AUROC of 0.819 for the direct detection of dementia from raw ECG signals, whereas prior work already demonstrated the feasibility of inferring dementia risk within a horizon of 5 years based on the ECG [69]. Similarly, we also report high predictive performance for diverse *substance disorders and dependencies* such as opioids, cocaine, alcohol, and tobacco, which are know for leaving traces in the ECG [70].
VI: Diseases of the nervous system Recent studies [71] highlight the potential to predict *Parkinson's disease* (PD) from ECGs achieving an AUROC score of 0.74 up to 1 year before diagnosis. We report 0.803 AUROC for PD (after diagnosis). *Alzheimer's* . Our AUROC score of 0.803 (after detection) is in line with this finding. Previous studies demonstrate certain ECG abnormalities on patients with *polyneuropathy* [72], we report 0.832 AUROC for its detection. Finally, we also found a certain degree of significance for *anoxic brain damage* with 0.813 AUROC, which to the best of our knowledge has not been investigated before.
X: Diseases of the respiratory system *Respiratory failure* correlations with ECGs have previously been investigated based on abnormal ECG features in pediatrics prognosis [73], the presence of cardiac arrhythmias in respiratory failure from chronic obstructive pulmonary disease [74] and respiratory signal extraction from ECGs [75]. We report AUROCs of 0.923 for acute and chronic respiratory failure and 0.951 with the additional presence of *hypoxia*. Specific ECG feature abnormalities were found in a single patient with *malignant pleural effusion* [76], we report an AUROC of 0.904 for malignant pleural effusion detection. A similar study displayed specific heart conditions and ECG signal abnormalities with the presence of *bronchiectasis* [77], we report an AUROC of 0.803, as well as 0.817 for *atelectasis*, 0.883 for *interstitial pulmonary diseases*, and 0.801 for *asthma*.
XI: Diseases of the digestive system Recent studies around liver conditions such as *cirrhosis* have been able to differentiate ECG changes between patients with cirrhosis and hepatitis [78], as well developing predictive models for its detection [25] with AUROC of 0.908. We report 0.906 AUROC for cirrhosis detection as well as 0.973 for cirrhosis with ascites. Furthermore, we also are able to predict with significant AUROCs diverse additional conditions which to the best of our knowledge, have not been investigated in similar predictive settings such as *alcoholic hepatitis* 0.930, *hemorrhagic necrosis of liver* 0.948, *hepatic failure* 0.947, *gastroparesis* 0.867, *peritonitis* 0.853, and *cholangitis* 0.848.

XII: Diseases of the skin and subcutaneous tissue The detection of *ulcers* by ECG has not been investigated in detail, however, there are some works that found a correlation between ECG changes and upper gastrointestinal bleeding [79], as well as peptic ulcer detection from ECG and additional predictors such as respiration rate, heart rate, pH of saliva, and temperature [80]. We report significant AUROCs on *pressure ulcers* at different locations such as back 0.975, and sacral region 0.874, as well as *non-pressure ulcers* such as heel and midfoot 0.87, lower limb 0.819, and foot 0.811. Similarly, for *acute lymphangitis* we report an AUROC score of 0.851.

XIII: Diseases of the musculoskeletal system and connective tissue *Gout*, specifically serum uric acid levels, have been previously linked to ECG abnormalities [81].At the time of the publication, we are not aware of any work that detects gout by a single ECG, a task for which we report an AUROC score of 0.803. Similarly, *lupus* have been found to affect ECG signals [82]. This is in line with an AUROC score of 0.801 for lupus detection from ECG achieved by our model. Our robust approach also allows us to detect diverse musculoskeletal and tissue diseases such as *arthropath* with 0.847, *cartilage disorder* with 0.819, and *disorders of bone density and structure* with 0.804 of AUROCs.

XIV: Diseases of the genitourinary system *Chronic kidney disease* has been previously detected through ECGs with an AUROC of 0.767 [50], we report an AUROC of 0.834. *Benign prostatic hyperplasia* have been shown to increase the incident of atrial fibrillation [83], we report an AUROC of 0.811 for its detection. Patients with reduced glomerular filtration rate are more likely to have a history of cardiovascular diseases [84], we report an AUROC of 0.878 for the detection of *glomerular disease*. Kidney failure, particularly in end-stage renal disease patients is associated with an increased risk of sudden cardiac death by low heart rate variability [85], we report an AUROC of 0.852 for *kidney failure with tubular necrosis* detection.

XVII: Congenital malformations, deformations and chromosomal abnormalities Although congenital heart valve malformations are one of the most common types of birth defects, their severity is diverse. Most of these malformations are diagnosed by echocardiograms, however, early works provided a review that summarizes specific ECG abnormalities for various malformations [86]. Similarly, another work predicted mitral valve prolapse from ECGs either congenital or not with an AUROC of 0.80 [51]. We report an AUROC of 0.863 for *congenital malformations of aortic and mitral valves*.

XVIII: Symptoms, signs and abnormal clinical and laboratory findings It has been shown that systemic inflammation increases the risk for atrial fibrillation [87], we report an AUROC of 0.870 for *systemic inflammation* detection. Although *cardiogenic shock* is difficult to diagnose due to variable presentations, overlapping with other shock states, and specialized test requirements, previous works were able to identify it at ED by ECGs and ultrasounds [88].We report a 0.931 AUROC for its detection. To the best of our knowledge, *cachexia* has not been detected by an ECG alone, however, as it is linked to an increased risk of arrhythmias, and can cause electrolyte imbalances it seems a possible task to achieve, for which we report an AUROC of 0.900.

XIX: Injury, poisoning and certain other consequences of external causes *Patient poisoning* due to administrated drugs can cause diverse effects ranging from rash to brain damage, coma, or death. A previous work [89] explored with a controlled study the changes that ECGs suffer from patients with acute cardiotoxicity, classifying these by specific ion channelsBased on the nature of our ICD-10 labeling, we report significant AUROCs for diverse poisoning based on groups of drugs, such as anticoagulants 0.880, antibiotics 0.872, antineoplastic and immunosuppressive drugs 0.870, systemic and hematological agents 0.851, hormones 0.841, diuretics 0.824, and opioids 0.817. Similarly, as injury we report 0.8345 of AUROC for *pertrochanteric fracture*.

XX: External causes of morbidity and mortality In the domain of *traffic accidents*, a prior study [90] measured survivors' HRV within 24 hours post-accident, at 2 months, and 6 months thereafter. The findings underscore a consistent diminution in HRV over time. Similarly, other works investigated specific body damaged areas such as blunt cardiac injuries [91], however, concluded that an ECG on its own does not achieve significant sensitivity. Surprisingly, our model excels in predicting various ICD-10 codes associated with *road traffic and non-traffic accidents* with high AUROCs.

XXI: Factors influencing health status and contact with health services With regard to health status, a previous work explored ECG changes across different *body mass indexes* of healthy individuals [92].Our work shows high discriminative power for diverse indexes, especially for extreme ones such as <19.9 and 50-59 with 0.944, and 0.940 AUROCs, respectively. Surprisingly, our model predicts with 0.841 AUROC the *absence of limb*, which highlights possible research directions in terms of limb prosthesis acceptance or rejection, for which a previous work based on patient metadata found significant results [93]. Similarly, our models report an AUROC of 0.935 for the *encounter for adjustment and management of implanted cardiac devices*, which also highlight possible research directions and application in terms of the reduction of adverse events with patient timely intervention by remote device monitoring.

Model architectures

In this work, we consider two model architectures, an example of a state-of-the-art convolutional model as a representative of this most widely model architecture and a structured state space model as a contender that showed statistically significant improvements over convolutional architectures in a previous study. We summarize both model architectures graphically in Fig. A.1.

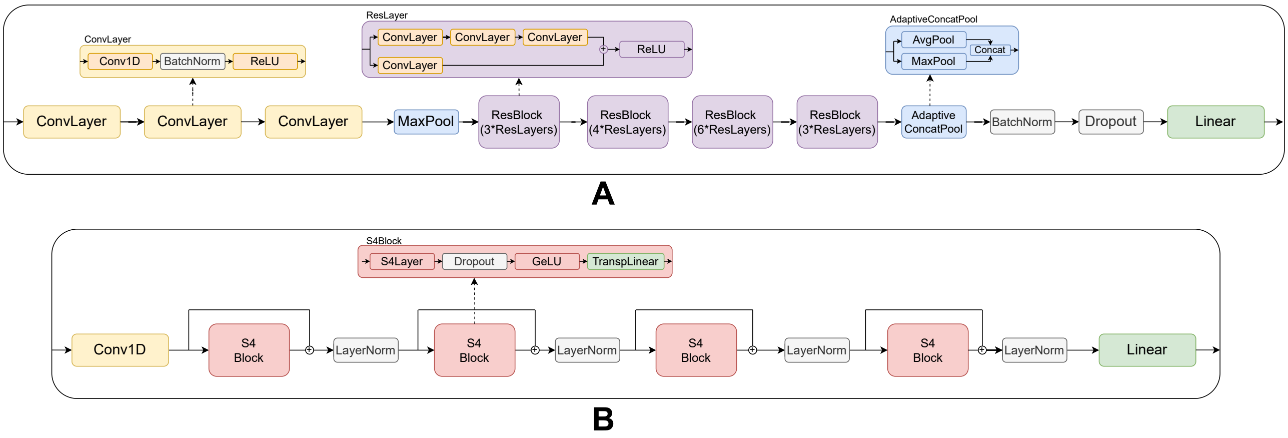

Figure A.1: Schematic representation of the two model architectures considered in this work: (A) XResNet1d50 (B) S4-model.

A recent benchmarking study on the PTB-XL dataset [5] established the superiority of modern ResNet- or Inception-based convolutional architectures over other deep learning-based architectures and feature-based approaches. This is in line with the use of ResNet as the predominant model architecture for deep-learning-based ECG analysis. The XResNet1d family introduced in [5] is a family of convolutional models as adaptations of the popular XResNet models [94] from image recognition to one-dimensional data. In particular, the XResNet1d50 architecture considered here is a convolutional neural network for 1D data that consists of 4 residual blocks, each with a set of 3,4,6, and 3 convolutional layers.

Structured state space models represent a recently proposed alternative [23] to convolutional architectures which demonstrated compelling abilities in capturing long-range dependencies in sequential data such as time series, including physiological time series. It was used as a building block for time series imputation and forecasting [95] also covering applications to ECG data as well as the generation of synthetic ECG data [96]. A recent benchmarking study [22] established statistically significant improvements in this architecture over the existing state-of-the-art of mostly convolutional and recurrent architectures also for diagnostic ECG tasks, both in the supervised and in the self-supervised setting. Here, we closely follow the hyperparameter choices from [22], who used four bidirectional S4-layers with a model dimension of 512 and a state dimension of 8. The S4-layers preserve the temporal resolution of the input and can be seen as a replacement for a transformer, an RNN layer, or a convolutional layer with unit stride. The S4 layers are followed by an average pooling layer and a linear classification head.

MIMIC-IV-ECG-ICD-ED dataset

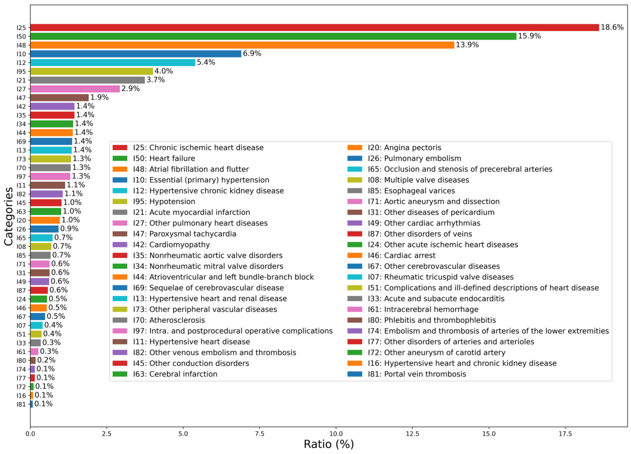

Figure A.2: Representation of the distribution of cardiac conditions in the ED dataset within chapter IX (Circulatory system diseases categories) at the 3rd digit level including all of its descendants, where category I25 (Chronic ischemic heart disease) is the most represented category with 19.6%, closely followed by I50 (heart failure) with 15.3%, I48 (Atrial fibrillation and flutter) with 13.5%, and I10 (Essential hypertension) with 10.7%.

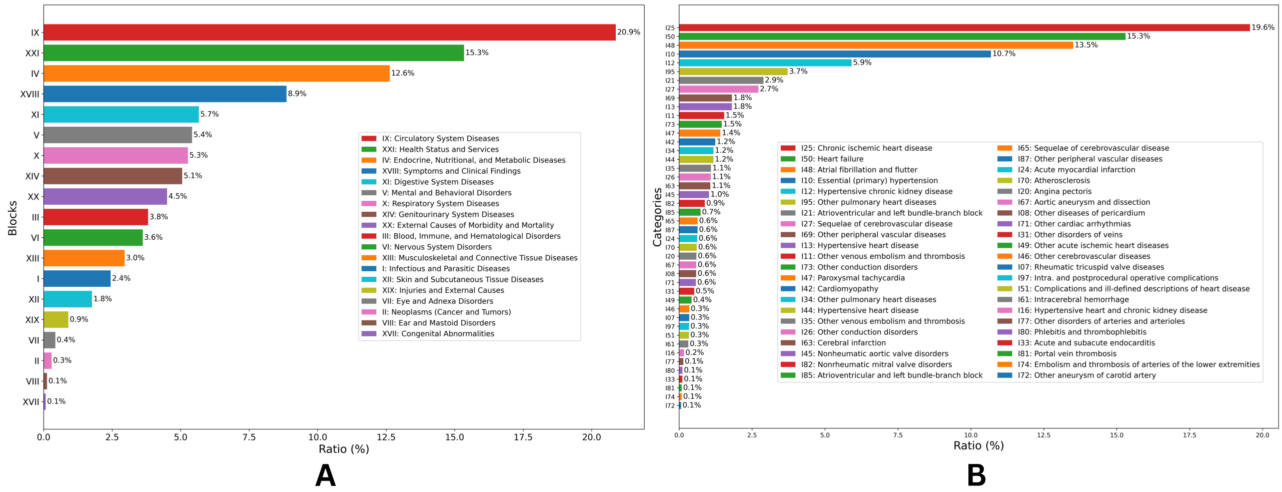

Figure A.3: Representation of the distribution of the full dataset instead of just the ED subset: Descriptive statistics on the MIMIC-IV-ECG-ICD dataset obtained by joining the MIMIC-IV-ECG with diagnoses from the clinical MIMIC-IV dataset: (A) represents the distribution of chapters, whereas (B) represents the distribution with the cardiovascular chapter IX.

In this section, we describe the ICD10 chapters and distributions of the processed MIMIC-IV-ECG database [21][97]. In Fig. A.3, we summarize the full dataset in terms of the distribution of chapters and IX categories. Fig. A.3A represents the distribution of chapter records, where chapter IX is most strongly represented chapter with 20.9%, closely followed by chapter XXI with 15.3%, chapter IV with 12.6%, and chapter XVIII with 8.9%. Fig. A.3B shows the distribution of codes within the cardiovascular chapter IX aggregated at the 3rd digit level with all of its descendants included, where category I25 is the most represented category with 18.6%, closely followed by I50 with 15.9%, I48 with 13.9%, and I10 with 6.9%.

Evaluation scenarios

Disagreement between ED and hospital discharge diagnoses Previous studies evaluated the agreement between diagnoses assigned in ED and those assigned after hospital discharge [98]. Some of their findings demonstrate that only 62.2% of ED diagnoses were concordant with hospital discharge diagnoses primarily for older patients and less urgent cases. However, there were other concordance factors such as an hour of the visit, and ED specialization degree. Thus, the correct diagnosis assignment at the ED represents a challenge not only as a system overall but also facility-wise, and it should be overcome as it has been shown that diagnoses that changed from ED to hospital discharge (wrong diagnosis at the ED department) had a 30% higher probability of death [98]. For this work, we accept a potential mismatch between ED diagnoses and hospital discharge diagnoses and take it into consideration during the model building process.

Comprehensive model T(ALL2ALL) We first want to introduce a training-testing scenario which we refer to as T(ALL2ALL)-E(ALL2ALL). The first half of the acronym refers to the training modality: The model was trained on all ECGs (hospital+ED), to predict all hospital discharge diagnoses (if available) otherwise ED diagnosis. The second half represents the evaluation scenario: The model was evaluated on all ECGs (hospital+ED) to predict all hospital discharge diagnoses (if available) otherwise ED diagnoses. In this scenario, both training and test distribution coincide both in terms of patient population as well as label selection. From the training T(ALL2ALL) strategy, we also consider four additional evaluation scenarios which differ based on the ECGs and department diagnosis subsets: T(ALL2ALL)-E(ALL2HOSP), T(ALL2ALL)-E(ED2ALL), T(ALL2ALL)-E(ED2HOSP), T(ALL2ALL)-E(ED2ED). The diagnoses label set in the evaluation scenario either for ED (2ED), or hospital (2HOSP) is restricted to these sets in contrast to all (2ALL).

Application scenarios The scenarios T(ALL2ALL)-E(ALL2ALL) and T(ALL2ALL)-E(ALL2HOSP) address the question of inferring general conditions from raw ECGs. Discharge diagnoses as compared to ED diagnoses represent the more comprehensive set of ICD codes with a potentially higher label certainty. However, the omission of ED ECGs without hospital admission in the T(ALL2ALL)-E(ALL2HOSP) scenario leads to a potentially severe bias in the target population as the subset of ED patients who did not get admitted to the hospital represents a large fraction of cases with typically less severe symptoms. The T(ALL2ALL)-E(ED2ALL), T(ALL2ALL)-E(ED2HOSP), and T(ALL2ALL)-(ED2ED) scenarios can be seen as potential applications for a triage use case. One can either predict the most comprehensive label set available, i.e., hospital discharge diagnosis if available or otherwise ED diagnosis or use only hospital diagnosis or ED diagnosis as targets. Note that the choice of hospital diagnoses (in scenarios T(ALL2ALL)-E(ALL2HOSP) and T(ALL2ALL)-E(ED2HOSP)) leads to a

substantial reduction in terms of applicable ED ECGs as all ECGs without subsequent hospital admission get excluded.
Distribution shift On the one hand, using an evaluation scenario that deviates from the training scenario T(ALL2ALL) inevitably leads to a mismatch between training and test distributions. On the other hand, one might expect that the models on the smaller selections profit from the training on the largest available dataset in scenario, i.e., T(ALL2ALL). However, our experiments demonstrate in terms of summary metrics such as macro AUROC that the advantage of training on a training dataset that matches the test distribution outperforms a model trained on potentially significantly larger training dataset. Therefore we present a model T(ED2ALL)-E(ED2ALL) that during training and evaluation only uses training ED ECGs to predict discharge diagnosis (if available) otherwise ED diagnosis, with an additional evaluation scenario T(ED2ALL)-E(ALL2ALL) which evaluated on all ECG subsets to predict discharge diagnosis (if available) otherwise ED diagnosis. Furthermore, we also present a model T(ED2ED)-E(ALL2ALL) trained on ED ECGs to predict ED labels and is evaluated on both subsets of ECGs to predict discharge diagnosis (if available) otherwise ED diagnosis.

Scenario	macro AUROC
**T(ALL2ALL)-E(ALL2ALL)**	**0.7505 (0.7478-0.7531)**
T(ED2ALL)-E(ALL2ALL)	0.7335 (0.7309-0.7366)
T(ED2ED)-E(ALL2ALL)	0.6777 (0.6736-0.6816)
T(ALL2ALL)-E(ED2ALL)	0.7691 (0.7651-0.7732)
**T(ED2ALL)-E(ED2ALL)**	**0.7742 (0.7703-0.7783)**
T(ALL2ALL)-E(ALL2HOSP)	0.7301 (0.7273-0.7330)
T(ALL2ALL)-E(ED2HOSP)	0.7394 (0.7350-0.7433)
T(ALL2ALL)-E(ED2ED)	0.6589 (0.6416-0.6568)

Table A.1: Model performance (S4-model) in terms of macro AUROC scores across different training and evaluation scenarios. The models highlighted in boldface correspond to the best-performing model for a given evaluation scenario. In the main text, the setup (ED2ALL)-E(ED2ALL) was used. Note that only scenarios with identical evaluation setups (separated by horizontal lines) are directly comparable.

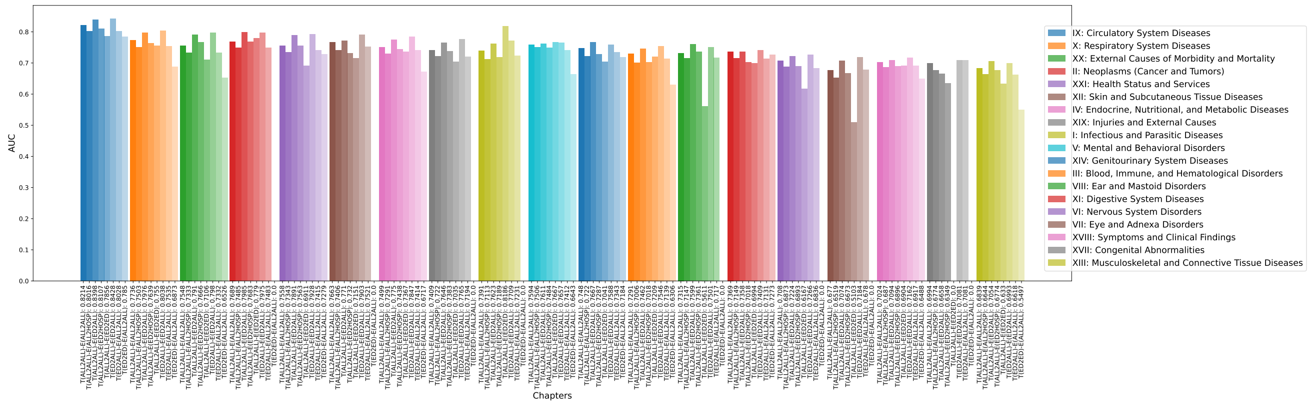

Figure A.4: The bar plot dissects all our investigated scenarios, representing diverse training and testing subsets characterized by varying ECG sources (ED, Hosp, or ALL) as well as distinct label sets (ED, Hosp, or ALL). Each color group showcases the predictive performance across each of the ICD-10 chapters This nuanced representation provides a concise yet comprehensive snapshot of health patterns across different scenarios, offering valuable insights into the impact of data source and label variation on predictive models for various medical conditions. The values chapters bar blocks are sorted by the T(ED2ALL)-E(ED2ALL) scenario. Similarly, the chapter's bar blocks are sorted left to right as they are presented in the legend box up to down.

Performance comparison E(ED2ALL) Fig. A.4 shows the model performance in each of the scenarios along with macro AUROC and confidence intervals. The model training on the full dataset and evaluated only on ED ECGs, i.e., T(ALL2ALL)-E(ED2ALL), yields a model that is only slightly weaker than a specialized model only trained on ED data, i.e., T(ED2ALL)-E(ED2ALL) (0.769 vs. 0.774). Nevertheless, as their scores overlap within error bars, we assessed the statistical significance of the performance difference using bootstrapping on the test set, where a confidence interval for the score difference that does not overlap with zero indicates a statistically significant result. The global model T(ALL2ALL)-E(ED2ALL) reported 76

conditions as significant while the T(ED2ALL)-E(ED2ALL) model 105, both models achieved 0.7691 and 0.7742 of macro AUROC respectively, where the difference also turns out significant, therefore the specialized model T(ED2ALL)-E(ED2ALL) is stronger. However, it is also worth stressing that the specialized ED model is considerably weaker on the set of all ECGs T(ED2ALL)-E(ALL2ALL) vs. T(ALL2ALL)-E(ALL2ALL) (0.734 vs. 0.751). Fig. A.4 shows the macro AUROC for each of the scenarios summarized according to ICD-10 chapters, providing a more comprehensive comparison of specific sorts of conditions for each of the possible clinical applications among departments. Bars with macro AUROC of 0 do not contain conditions at the specific chapter due to the absence of these during the label selection stage. Overall, the T(ED2ALL)-E(ED2ALL) model is best on all chapters except for II (Neoplasms), XIV (Diseases of the genitourinary system), VIII (Diseases of the ear and mastoid process), and XIII (Diseases of the musculoskeletal system and connective tissue). Furthermore, it is important to note that the additional scenario T(ALL2ALL)-E(ED2ED) is better than the two of them on chapters I (Certain infectious and parasitic diseases) and V (Mental and behavioural disorders).

Performance comparison E(ALL2ALL) The second scenario we investigate in more detail is the evaluation on both hospital and ED ECGs. To this end, we first compare T(ALL2ALL)-E(ALL2ALL), T(ED2ALL)-E(ALL2ALL), T(ED2ED)-E(ALL2ALL), which all evaluate all diagnoses from all ECGs (global perspective of any ECG), however, while trained on all records with all labels (global perspective), ED records with all labels (ED+Hospital screening use case at the ED department), and ED records on ED labels (ED screening use case at the ED department). Overall, the comprehensive model T(ALL2ALL)-E(ALL2ALL) is best on all chapters except for VII (Diseases of the eye and adnexa), and XIII (Diseases of the musculoskeletal system and connective tissue), where T(ED2ALL)-E(ALL2ALL) is better. Similarly, T(ED2ALL)-E(ALL2ALL) outperforms T(ED2ED)-E(ALL2ALL) in all ICD chapters except for XI (Diseases of the digestive system).

Statement	ICD10 codes	Internal	External
1AVB	I440	0.9007	0.9041
AFIB	I4891	0.8965	0.9872
LBBB	I447	0.9588	0.9990
RBBB	I4510	0.9384	0.9959
SBRAD	R001	0.7620	0.9368
STACH	R000	0.8305	0.9740

Table A.2: External validation for diverse cardiac conditions on the E(ALL2ALL) setting (1AVB: 1st degree AV block, AFIB: atrial fibriallation, LBBB: left bundle branch block, RBBB: right bundle branch block, SBRAD: sinus bradycardia, STACH: sinus tachycardia).

External validation E(ALL2ALL): Tab. A.2 presents the external validation scores for diverse cardiac conditions in the E(ALL2ALL) setting, once again validating an optimal performance outside of the training dataset distribution.

Comparing model architectures

Model	Scenario	macro AUROC	Significant labels
XResNet1d50	T(ALL2ALL)-E(ALL2ALL)	0.7475(0.7447-0.7504)	43/1076
**S4**	T(ALL2ALL)-E(ALL2ALL)	**0.7504(0.7478-0.7531)**$*$	**161/1076**
XResNet1d50	T(ED2ALL)-E(ED2ALL)	0.7724 (0.7682-0.7761)	42/1076
**S4**	T(ED2ALL)-E(ED2ALL)	**0.7742 (0.7703-0.7783)**	**104/1076**

Table A.3: Comparison of predictive performance between the XResNet1d50 and S4 models on the proposed MIMIC-IV-ECG-ICD-ED dataset. The table shows the macro AUROCs, and the resulting performances of a bootstrap-powered hypothesis test in terms of significant labels and macro AUROC for each model at the settings T(ALL2ALL)-E(ALL2ALL), and T(ED2ALL)-E(ED2ALL) with the S4 model outperforming the XResNet1d50 model in both scenarios. Confidence intervals for AUROC scores are provided in parentheses. The fourth column indicates the number of statements for which the model outperforms its competitor (in the sense of bootstrapping confidence intervals for the score difference not overlapping with zero). Bold values indicate the best-performing model in both scenarios. In both cases the performance difference between both models in terms of macro AUROC is statistically significant.

In this section, we provide a comparative assessment of two model architectures that have shown competitive performance on the PTB-XL dataset, see [5, 22]. More specifically, we compare an XResNet1d50 model, a convolutional neural network

with ResNet architecture, to an S4 model leveraging the recently proposed structured state-space models [23], see Methods for details. As in [5], we report AUROCs for the respective statements in the label set. Tab. A.3 shows the predictive performance on the T(ALL2ALL)-E(ALL2ALL) setting for the XResNet1d50 model, showcasing a macro AUROC of 0.7475, while the S4 model achieved a slightly higher AUROC of 0.7504. However, as their confidence intervals overlap, we assessed the statistical significance of the performance difference using bootstrapping on the test set. The XResNet1d50 model reported 43 labels as significant whereas the S4 model 161, both models achieved a macro AUROC on the hypothesis test of 0.7475 and 0.7505 respectively. Similarly, on the T(ED2ALL)-E(ED2ALL) setting, the XResNet1d50 model, showcased a macro AUROC of 0.7724, while the S4 model achieved a slightly higher AUROC of 0.7742. Again, we performed a bootstrap-powered hypothesis test to properly distinguish the significance of their performances. The XResNet1d50 model reported 42 labels as significant whereas the S4 model 104, both models achieved a macro AUROC on the hypothesis test of 0.7724 and 0.7774 respectively. Therefore, the S4 model outperforms the ResNet in terms of predictive performance in both scenarios.

Complete results tables

T(ED2ALL)-E(ED2ALL) Model: Statements with top label AUROCs

Tab. A.4 shows as extended version of Tab. 1, i.e., the best-performing ICD statements.In Tab. A.5 we summarize very accurately predictable statements (AUROC>0.9) aggregated at a 3-digit level. In Tab. A.6-Tab. A.9 we summarize accurately predictable statements (AUROC>0.8). Finally in Tab. A.10-Tab. A.12, we show challenging statements (AUROC<0.7).

T(ALL2ALL)-E(ALL2ALL) Model: Statements with top label AUROCs

Tab. A.13 shows the analogue of Tab. A.4 for the T(ALL2ALL)-model, i.e., the best-performing ICD statements. In Tab. A.14 we summarize very accurately predictable statements (AUROC>0.9) aggregated at a 3-digit level. In Tab. A.15-Tab. A.17 we summarize accurately predictable statements (AUROC>0.8). Finally in Tab. A.18-Tab. A.20, we show challenging statements (AUROC<0.7). All statements refer to the model trained on ALL (ED and hospital) ECGs.

References: Supplementary material

[31] A. Bansal, D. Cantillon, S. Nakhla, R. Madden, E. Donnellan, M. Chung, J. Rickard, B. Baranowski, A. Hussein, W. Saliba, *et al.*, "Machine learning prediction of pericardial tamponade after atrial fibrillation ablation," *American Journal of Cardiology*, vol. 175, pp. 179–180, 2022.

[32] S. Hamada, J. Schroeder, R. Hoffmann, E. Altiok, A. Keszei, M. Almalla, A. Napp, N. Marx, and M. Becker, "Prediction of outcomes in patients with chronic ischemic cardiomyopathy by layer-specific strain echocardiography: A proof of concept," *Journal of the American Society of Echocardiography*, vol. 29, no. 5, pp. 412–420, 2016.

[33] M. Zhou, Y. Deng, Y. Liu, X. Su, and X. Zeng, "Echocardiography-based machine learning algorithm for distinguishing ischemic cardiomyopathy from dilated cardiomyopathy," *BMC Cardiovascular Disorders*, vol. 23, no. 1, p. 476, 2023.

[34] J.-m. Kwon, K.-H. Kim, K.-H. Jeon, S. Y. Lee, J. Park, and B.-H. Oh, "Artificial intelligence algorithm for predicting cardiac arrest using electrocardiography," *Scandinavian journal of trauma, resuscitation and emergency medicine*, vol. 28, pp. 1–10, 2020.

[35] B. Ambale-Venkatesh, X. Yang, C. O. Wu, K. Liu, W. G. Hundley, R. McClelland, A. S. Gomes, A. R. Folsom, S. Shea, E. Guallar, *et al.*, "Cardiovascular event prediction by machine learning: the multi-ethnic study of atherosclerosis," *Circulation research*, vol. 121, no. 9, pp. 1092–1101, 2017.

[36] O. Sehrawat, A. H. Kashou, and P. A. Noseworthy, "Artificial intelligence and atrial fibrillation," *Journal of Cardiovascular Electrophysiology*, vol. 33, p. 1932âĂŞ1943, Mar. 2022.

[37] A. H. Kashou, W.-Y. Ko, Z. I. Attia, M. S. Cohen, P. A. Friedman, and P. A. Noseworthy, "A comprehensive artificial intelligence–enabled electrocardiogram interpretation program," *Cardiovascular Digital Health Journal*, vol. 1, no. 2, 2020.

[38] L. Amaya Pascasio, M. Quesada López, J. M. García-Torrecillas, A. Arjona-Padillo, and P. Martínez Sánchez, "Development of a score to predict the paroxysmal atrial fibrillation in stroke patients: the screening for atrial fibrillation scale," *Frontiers in Neurology*, vol. 13, p. 900582, 2022.

[39] L. Hussain, I. A. Awan, W. Aziz, S. Saeed, A. Ali, F. Zeeshan, K. S. Kwak, *et al.*, "Detecting congestive heart failure by extracting multimodal features and employing machine learning techniques," *BioMed research international*, vol. 2020, 2020.

[40] K. Joon-myoung, K. Kyung-Hee, J. Ki-Hyun, K. H. Mee, K. M. Jeong, L. Sung-Min, S. P. Sang, P. Jinsik, C. R. Kyeong, and O. Byung-Hee, "Development and validation of deep-learning algorithm for electrocardiography-based heart failure identification," *kcj*, vol. 49, no. 7, pp. 629–639, 2019.

[41] D. G. Vidt and L. M. Prisant, "Hypertensive heart disease," *The Journal of Clinical Hypertension*, vol. 7, no. 4, pp. 231–238, 2005.

[42] A. Schoenenberger, P. Erne, S. Ammann, M. Perrig, U. Bürgi, and A. Stuck, "Prediction of hypertensive crisis based on average, variability and approximate entropy of 24-h ambulatory blood pressure monitoring," *Journal of human hypertension*, vol. 22, no. 1, pp. 32–37, 2008.

[43] M. K. Hoffman, N. Ma, and A. Roberts, "A machine learning algorithm for predicting maternal readmission for hypertensive disorders of pregnancy," *American Journal of Obstetrics & Gynecology MFM*, vol. 3, no. 1, p. 100250, 2021.

[44] C.-M. Liu, E. S. Shih, J.-Y. Chen, C.-H. Huang, I.-C. Wu, P.-F. Chen, S. Higa, N. Yagi, Y.-F. Hu, M.-J. Hwang, *et al.*, "Artificial intelligence-enabled electrocardiogram improves the diagnosis and prediction of mortality in patients with pulmonary hypertension," *JACC: Asia*, vol. 2, no. 3_Part_1, pp. 258–270, 2022.

[45] M. A. Aras, S. Abreau, H. Mills, L. Radhakrishnan, L. Klein, N. Mantri, B. Rubin, J. Barrios, C. Chehoud, E. Kogan, X. Gitton, A. Nnewihe, D. Quinn, C. Bridges, A. J. Butte, J. E. Olgin, and G. H. Tison, "Electrocardiogram detection of pulmonary hypertension using deep learning," *Journal of Cardiac Failure*, vol. 29, no. 7, pp. 1017–1028, 2023.

[46] Y. Zhao, J. Xiong, Y. Hou, M. Zhu, Y. Lu, Y. Xu, J. Teliewubai, W. Liu, X. Xu, X. Li, *et al.*, "Early detection of st-segment elevated myocardial infarction by artificial intelligence with 12-lead electrocardiogram," *International Journal of Cardiology*, vol. 317, pp. 223–230, 2020.

[47] S. Gustafsson, D. Gedon, E. Lampa, A. H. Ribeiro, M. J. Holzmann, T. B. Schön, and J. Sundström, "Development and validation of deep learning ecg-based prediction of myocardial infarction in emergency department patients," *Scientific Reports*, vol. 12, no. 1, p. 19615, 2022.

[48] J.-m. Kwon, Y. R. Lee, M.-S. Jung, Y.-J. Lee, Y.-Y. Jo, D.-Y. Kang, S. Y. Lee, Y.-H. Cho, J.-H. Shin, J.-H. Ban, *et al.*, "Deep-learning model for screening sepsis using electrocardiography," *Scandinavian Journal of Trauma, Resuscitation and Emergency Medicine*, vol. 29, no. 1, pp. 1–12, 2021.

[49] Y.-T. Lee, C.-S. Lin, W.-H. Fang, C.-C. Lee, C.-L. Ho, C.-H. Wang, D.-J. Tsai, and C. Lin, "Artificial intelligence-enabled electrocardiography detects hypoalbuminemia and identifies the mechanism of hepatorenal and cardiovascular events," *Frontiers in Cardiovascular Medicine*, vol. 9, 2022.

[50] L. Holmstrom, M. Christensen, N. Yuan, J. Weston Hughes, J. Theurer, M. Jujjavarapu, P. Fatehi, A. Kwan, R. K. Sandhu, J. Ebinger, *et al.*, "Deep learning-based electrocardiographic screening for chronic kidney disease," *Communications Medicine*, vol. 3, no. 1, p. 73, 2023.

[51] G. H. Tison, S. Abreau, J. Barrios, L. J. Lim, M. Yang, V. Crudo, D. J. Shah, T. Nguyen, G. Hu, S. Dixit, G. Nah, F. Arya, D. Bibby, Y. Lee, and F. N. Delling, "Identifying mitral valve prolapse at risk for arrhythmias and fibrosis from electrocardiograms using deep learning," *JACC. Advances*, vol. 2, p. 100446, August 2023.

[52] V.-E. Neagoe, I.-F. Iatan, and S. Grunwald, "A neuro-fuzzy approach to classification of ecg signals for ischemic heart disease diagnosis," in *AMIA Annual Symposium Proceedings*, vol. 2003, p. 494, American Medical Informatics Association, 2003.

[53] J. P. Singh, J. Fontanarava, G. de Massé, T. Carbonati, J. Li, C. Henry, and L. Fiorina, "Short-term prediction of atrial fibrillation from ambulatory monitoring ECG using a deep neural network," *European Heart Journal - Digital Health*, vol. 3, pp. 208–217, 04 2022.

[54] P. Zhang, F. Lin, F. Ma, Y. Chen, S. Fang, H. Zheng, Z. Xiang, X. Yang, and Q. Li, "Automatic screening of patients with atrial fibrillation from 24-h Holter recording using deep learning," *European Heart Journal - Digital Health*, vol. 4, pp. 216–224, 03 2023.

[55] D. Martins, L. Agodoa, and K. C. Norris, "Hypertensive chronic kidney disease in african americans: strategies for improving care," *Cleveland Clinic journal of medicine*, vol. 79, no. 10, p. 726, 2012.

[56] Y. Sandoval, S. E. Thordsen, S. W. Smith, K. M. Schulz, M. M. Murakami, L. A. Pearce, and F. S. Apple, "Cardiac troponin changes to distinguish type 1 and type 2 myocardial infarction and 180-day mortality risk," *European Heart Journal. Acute Cardiovascular Care*, vol. 3, pp. 317–325, 12 2014.

[57] N. Strodthoff and C. Strodthoff, "Detecting and interpreting myocardial infarction using fully convolutional neural networks," *Physiological Measurement*, vol. 40, p. 015001, jan 2019.

[58] A. Vaid, E. Argulian, S. Lerakis, B. K. Beaulieu-Jones, C. Krittanawong, E. Klang, J. Lampert, V. Y. Reddy, J. Narula, G. N. Nadkarni, and B. S. Glicksberg, "Multi-center retrospective cohort study applying deep learning to electrocardiograms to identify left heart valvular dysfunction," *Communications medicine*, vol. 3, p. 24, February 2023.

[59] G. H. Tison, J. Zhang, F. N. Delling, and R. C. Deo, "Automated and interpretable patient ecg profiles for disease detection, tracking, and discovery," *Circulation: Cardiovascular Quality and Outcomes*, vol. 12, no. 9, p. e005289, 2019.

[60] J. S. Haimovich, N. Diamant, S. Khurshid, P. Di Achille, C. Reeder, S. Friedman, P. Singh, W. Spurlock, P. T. Ellinor, A. Philippakis, P. Batra, J. E. Ho, and S. A. Lubitz, "Artificial intelligence-enabled classification of hypertrophic heart diseases using electrocardiograms," *Cardiovascular Digital Health Journal*, vol. 4, pp. 48–59, Apr. 2023.

[61] A. Sadeghi, A. Rezaee, and F. Hajati, "Deep conv-attention model for diagnosing left bundle branch block from 12-lead electrocardiograms," *arXiv preprint arXiv:2212.04936*, 2022.

[62] D. U. Jeong and K. M. Lim, "Convolutional neural network for classification of eight types of arrhythmia using 2d time–frequency feature map from standard 12-lead electrocardiogram," *Scientific reports*, vol. 11, no. 1, p. 20396, 2021.

[63] D. Heck and A. Jost, "Carotid stenosis, stroke, and carotid artery revascularization," *Progress in Cardiovascular Diseases*, vol. 65, pp. 49–54, 2021.

[64] E. Astorri, A. Bonetti, and P. Fiorina, "Ecg mimicking acute myocardial infarction during heart involvement by lung neoplasm," *International Journal of Cardiology*, vol. 74, no. 2, pp. 225–226, 2000.

[65] È. Spînu, G. Cismaru, P.-M. Boarescu, S. Istratoaie, A. G. Negru, C. Lazea, S. S. Căinap, D. Iacob, A. I. Grosu, G. Saraci, *et al.*, "Ecg markers of cardiovascular toxicity in adult and pediatric cancer treatment," *Disease Markers*, vol. 2021, 2021.

[66] J. Pohl, R.-I. Mincu, S. M. Mrotzek, L. Hinrichs, L. Michel, E. Livingstone, L. Zimmer, R. Wakili, D. Schadendorf, T. Rassaf, *et al.*, "Ecg changes in melanoma patients undergoing cancer therapy-data from the ecor registry," *Journal of Clinical Medicine*, vol. 9, no. 7, p. 2060, 2020.

[67] S. Stern and S. Sclarowsky, "The ecg in diabetes mellitus," *Circulation*, vol. 120, no. 16, pp. 1633–1636, 2009.

[68] G. Swapna, K. Soman, and R. Vinayakumar, "Diabetes detection using ecg signals: An overview," *Deep Learning Techniques for Biomedical and Health Informatics*, pp. 299–327, 2020.

[69] J. L. Isaksen, J. Ghouse, M. W. Skov, M. S. Olesen, A. G. Holst, A. Pietersen, J. B. Nielsen, A. Maier, C. Graff, R. Frikke-Schmidt, and J. K. Kanters, "Associations between primary care electrocardiography and non-alzheimer dementia," *Journal of Stroke and Cerebrovascular Diseases*, vol. 31, no. 9, p. 106640, 2022.

[70] G. Sereny, "Effects of alcohol on the electrocardiogram," *Circulation*, vol. 44, pp. 558–564, Oct. 1971.

[71] I. Karabayir, F. Gunturkun, L. Butler, S. M. Goldman, R. Kamaleswaran, R. L. Davis, K. Colletta, L. Chinthala, J. L. Jefferies, K. Bobay, *et al.*, "Externally validated deep learning model to identify prodromal parkinson's disease from electrocardiogram," *Scientific Reports*, vol. 13, no. 1, p. 12290, 2023.

[72] A. Beckman, P. Bjerle, and B.-O. Olofsson, "Electrocardiographic findings in familial amyloidotic polyneuropathy," *American Journal of noninvasive cardiology*, vol. 6, no. 3, pp. 192–196, 1992.

[73] J. D. Keith, V. Rose, M. Braudo, and R. D. Rowe, "The electrocardiogram in the respiratory distress syndrome and related cardiovascular dynamics," *The Journal of Pediatrics*, vol. 59, no. 2, pp. 167–187, 1961.

[74] R. A. Incalzi, R. Pistelli, L. Fuso, A. Cocchi, M. G. Bonetti, and A. Giordano, "Cardiac arrhythmias and left ventricular function in respiratory failure from chronic obstructive pulmonary disease," *Chest*, vol. 97, no. 5, pp. 1092–1097, 1990.

[75] A. Travaglini, C. Lamberti, J. DeBie, and M. Ferri, "Respiratory signal derived from eight-lead ecg," in *Computers in Cardiology 1998. Vol. 25 (Cat. No. 98CH36292)*, pp. 65–68, IEEE, 1998.

[76] C. A. Manthous, G. A. Schmidt, and J. B. Hall, "Pleural effusion masquerading as myocardial infarction," *Chest*, vol. 103, no. 5, pp. 1619–1621, 1993.

[77] F. Alhamed Alduihi, "Ecg abnormalities in patients with acute exacerbation of bronchiectasis and factors associated with high probability of abnormality," *Pulmonary Medicine*, vol. 2021, 2021.

[78] L. Toma, A. M. Stanciu, A. Zgura, N. Bacalbasa, C. Diaconu, and L. Iliescu, "Electrocardiographic changes in liver cirrhosis-clues for cirrhotic cardiomyopathy," *Medicina*, vol. 56, no. 2, p. 68, 2020.

[79] G. O. Fa, M. E. LF, *et al.*, "Electrocardiographic changes related to acute upper gastrointestinal hemorrhage," *Anales de Medicina Interna (Madrid, Spain: 1984)*, vol. 23, no. 5, pp. 220–223, 2006.

[80] M. Faiz, M. Murad, R. Khalid, T. Mushtaq Shaikh, E. Ali, M. Shah, N. Ejaz, and S. J. Khan, "Aiding gastrointestinal diagnostic laboratory by designing a device for the non invasive detection of peptic ulcer," *Proceedings of the Institution of Mechanical Engineers, Part H: Journal of Engineering in Medicine*, vol. 237, no. 8, pp. 928–935, 2023.

[81] A. F. Cicero, M. Rosticci, A. Reggi, G. Derosa, A. Parini, E. Grandi, S. DâĂŹAddato, and C. Borghi, "Relationship between serum uric acid and electrocardiographic alterations in a large sample of general population: data from the brisighella heart study," *High Blood Pressure & Cardiovascular Prevention*, vol. 22, pp. 129–134, 2015.

[82] Z. Hu, L. Wu, Z. Lin, X. Liu, C. Zhao, and Z. Wu, "Prevalence and associated factors of electrocardiogram abnormalities in patients with systemic lupus erythematosus: a machine learning study," *Arthritis Care & Research*, vol. 74, no. 10, pp. 1640–1648, 2022.

[83] A. Koçak, C. Şenol, A. Coşgun, F. Eyyupkoca, and O. Yıldırım, "The relationship of benign prostatic hyperplasia's symptoms severity with the risk of developing atrial fibrillation," *Journal of Arrhythmia*, vol. 38, no. 2, pp. 232–237, 2022.

[84] M. Rahman, C. D. Brown, J. Coresh, B. R. Davis, J. H. Eckfeldt, N. Kopyt, A. S. Levey, C. Nwachuku, S. Pressel, E. Reisin, C. Walworth, and for the ALLHAT Collaborative Research Group, "The Prevalence of Reduced Glomerular Filtration Rate in Older Hypertensive Patients and Its Association With Cardiovascular Disease: A Report From the Antihypertensive and Lipid-Lowering Treatment to Prevent Heart Attack Trial," *Archives of Internal Medicine*, vol. 164, pp. 969–976, 05 2004.

[85] R. Ranpuria, M. Hall, C. T. Chan, and M. Unruh, "Heart rate variability (HRV) in kidney failure: measurement and consequences of reduced HRV," *Nephrology Dialysis Transplantation*, vol. 23, pp. 444–449, 11 2007.

[86] P. Khairy and A. J. Marelli, "Clinical use of electrocardiography in adults with congenital heart disease," *Circulation*, vol. 116, no. 23, pp. 2734–2746, 2007.

[87] P. E. Lazzerini, F. Laghi-Pasini, M. Acampa, U. Srivastava, I. Bertolozzi, B. Giabbani, F. Finizola, F. Vanni, A. Dokollari, M. Natale, *et al.*, "Systemic inflammation rapidly induces reversible atrial electrical remodeling: The role of interleukin-6–mediated changes in connexin expression," *Journal of the American Heart Association*, vol. 8, no. 16, p. e011006, 2019.

[88] M. Daly, B. Long, A. Koyfman, and S. Lentz, "Identifying cardiogenic shock in the emergency department," *The American Journal of Emergency Medicine*, vol. 38, no. 11, pp. 2425–2433, 2020.

[89] C. Yates and A. F Manini, "Utility of the electrocardiogram in drug overdose and poisoning: theoretical considerations and clinical implications," *Current cardiology reviews*, vol. 8, no. 2, pp. 137–151, 2012.

[90] A. Shaikh al arab, L. GuÃl'don-Moreau, F. Ducrocq, S. Molenda, S. Duhem, J. Salleron, I. Chaudieu, D. Bert, C. Libersa, and G. Vaiva, "Temporal analysis of heart rate variability as a predictor of post traumatic stress disorder in road traffic accidents survivors," *Journal of Psychiatric Research*, vol. 46, no. 6, pp. 790–796, 2012.

[91] I. P. Kyriazidis, D. A. Jakob, J. A. H. Vargas, O. H. Franco, E. Degiannis, P. Dorn, S. Pouwels, B. Patel, I. Johnson, C. J. Houdlen, *et al.*, "Accuracy of diagnostic tests in cardiac injury after blunt chest trauma: a systematic review and meta-analysis," *World journal of emergency surgery*, vol. 18, no. 1, p. 36, 2023.

[92] G. Hassing, H. Van der Wall, G. Van Westen, M. Kemme, A. Adiyaman, A. Elvan, J. Burggraaf, and P. Gal, "Body mass index related electrocardiographic findings in healthy young individuals with a normal body mass index," *Netherlands Heart Journal*, vol. 27, pp. 506–512, 2019.

[93] E. A. Biddiss and T. T. Chau, "Multivariate prediction of upper limb prosthesis acceptance or rejection," *Disability and Rehabilitation: Assistive Technology*, vol. 3, no. 4, pp. 181–192, 2008.

[94] T. He, Z. Zhang, H. Zhang, Z. Zhang, J. Xie, and M. Li, "Bag of tricks for image classification with convolutional neural networks," in *Proceedings of the IEEE/CVF conference on computer vision and pattern recognition*, pp. 558–567, 2019.

[95] J. M. L. Alcaraz and N. Strodthoff, "Diffusion-based time series imputation and forecasting with structured state space models," *Transactions on Machine Learning Research*, 2022.

[96] J. M. L. Alcaraz and N. Strodthoff, "Diffusion-based conditional ECG generation with structured state space models," *Computers in Biology and Medicine*, vol. 163, p. 107115, 2023.

[97] A. Johnson, L. Bulgarelli, T. Pollard, S. Horng, L. A. Celi, and R. Mark, "Mimic-iv," 2023.

[98] S. Farchi, L. Camilloni, P. G. Rossi, F. Chini, G. Lori, V. Tancioni, P. Papini, P. Borgia, and G. Guasticchi, "Agreement between emergency room and discharge diagnoses in a population of injured inpatients: Determinants and mortality," *The Journal of Trauma: Injury, Infection, and Critical Care*, vol. 62, p. 1207âĂŞ1214, May 2007.

Block: Block description. Block AUROC	Code: Code AUROC. Code description	Code: Code AUROC. Code description
I: Infectious and Parasitic Diseases. 0.7709	B9620: 0.862. E. coli<br>A41: 0.857. Other sepsis<br>A047: 0.809. Enterocolitis due to Clostridium difficile	A40: 0.859. Streptococcal sepsis<br>B370: 0.823. Candidal stomatitis
II: Neoplasms (Cancer and Tumors). 0.7975	**C7952: 0.933. Malignant neoplasm of bone marrow**<br>C25: 0.884. Malignant neoplasm of pancreas<br>C8589: 0.853. Other specified types of non-Hodgkin lymphoma	**C925: 0.922. Acute myelomonocytic leukemia**<br>D469: 0.869. Myelodysplastic syndrome<br>C34: 0.826. Malignant neoplasm of bronchus and lung
III: Blood, Immune, and Hematological Disorders. 0.7546	**D65: 0.933. Disseminated intravascular coagulation**<br>D631: 0.857. Anemia in chronic kidney disease	D684: 0.878. Acquired coagulation factor deficiency<br>D618: 0.812. Other specified aplastic anemias and other bone marrow failure syndromes
IV: Endocrine, Nutritional, and Metabolic Diseases. 0.7847	**E1129: 0.924. Type 2 diabetes mellitus with diabetic kidney complication**<br>E103: 0.899. Type 1 diabetes mellitus with ophthalmic complications<br>E1342: 0.880. Diabetes mellitus with diabetic polyneuropathy	**E660: 0.907. Obesity due to excess calories**<br>E43: 0.886. Severe protein-calorie malnutrition<br>E8770: 0.874. Fluid overload
V: Mental and Behavioral Disorders. 0.7657	F1022: 0.894. Alcohol dependence with intoxication<br>F4310: 0.864. Post-traumatic stress disorder<br>F141: 0.826. Cocaine abuse	F1721: 0.880. Nicotine dependence<br>F11: 0.833. Opioid-related disorders<br>F039: 0.819. Unspecified dementia
VI: Nervous System Disorders. 0.7266	G318: 0.838. Other specified degenerative diseases of the nervous system<br>G609: 0.827. Hereditary and idiopathic neuropathy<br>G931: 0.813. Anoxic brain damage<br>G20: 0.803. Parkinson's disease	G629: 0.832. Polyneuropathy, unspecified<br>G893: 0.822. Neoplasm-related pain (acute) (chronic)<br>G309: 0.806. Alzheimer's disease
VIII: Ear and Mastoid Disorders. 0.7501	H9190: 0.809. Unspecified hearing loss, unspecified ear	
IX: Circulatory System Diseases. 0.8428	**I210: 0.986. ST elevation (STEMI) myocardial infarction of anterior wall**<br>**I447: 0.976. Left bundle-branch block**<br>**I451: 0.964. Other right bundle-branch block**<br>**I132: 0.949. Hypertensive heart failure and chronic kidney disease or end-stage renal disease**<br>**I081: 0.944. Rheumatic disorders of both mitral and tricuspid valves**<br>**I5043: 0.940. Acute on chronic combined systolic and diastolic heart failure**	**I314: 0.979. Cardiac tamponade**<br>**I481: 0.966. Persistent atrial fibrillation**<br>**I255: 0.964. Ischemic cardiomyopathy**<br>**I078: 0.948. Other rheumatic tricuspid valve diseases**<br>**I2789: 0.943. Other specified pulmonary heart diseases**<br>**I7025: 0.927. Atherosclerosis of native arteries with ulceration**
X: Respiratory System Diseases. 0.8038	**J9621: 0.951. Acute and chronic respiratory failure with hypoxia**<br>**J80: 0.905. Acute respiratory distress syndrome**<br>J848: 0.883. Interstitial pulmonary diseases	**J94: 0.917. Other pleural conditions**<br>**J910: 0.904. Malignant pleural effusion**<br>J90: 0.879. Pleural effusion, not elsewhere classified
XI: Digestive System Diseases. 0.7409	**K7031: 0.973. Alcoholic cirrhosis of the liver with ascites**<br>**K7290: 0.947. Hepatic failure without coma**<br>K65: 0.853. Peritonitis	**K762: 0.948. Central hemorrhagic necrosis of liver**<br>**K3189: 0.921. Other diseases of stomach and duodenum**<br>K830: 0.848. Cholangitis
XII: Skin and Subcutaneous Tissue Diseases. 0.7903	L891: 0.874. Pressure ulcer of back<br>L0312: 0.851. Acute lymphangitis of a limb part	L9740: 0.870. Non-pressure chronic ulcer
XIII: Musculoskeletal and Connective Tissue Diseases. 0.6903	M129: 0.847. Arthropathy, unspecified<br>M858: 0.805. Other specified disorders of bone density and structure<br>M10: 0.803. Gout	M949: 0.819. Disorder of cartilage<br>M353: 0.805. Polymyalgia rheumatica<br>M321: 0.801. Systemic lupus erythematosus with organ or system involvement
XIV: Genitourinary System Diseases. 0.7588	N186: 0.887. End stage renal disease<br>N9982: 0.857. Postprocedural hemorrhage of a genitourinary system organ<br>N40: 0.811. Benign prostatic hyperplasia	N08: 0.878. Glomerular disorders in diseases classified elsewhere<br>N170: 0.852. Acute kidney failure with tubular necrosis
XVIII: Symptoms and Clinical Findings. 0.7162	**R570: 0.931. Cardiogenic shock**<br>R18: 0.887. Ascites<br>R34: 0.882. Anuria and oliguria	**R64: 0.900. Cachexia**<br>R6521: 0.887. Severe sepsis with septic shock<br>R000: 0.849. Tachycardia, unspecified
XIX: Injuries and External Causes. 0.7765	**T8612: 0.944. Kidney transplant failure**<br>T380: 0.898. Poisoning by, adverse effect of and underdosing of glucocorticoids and synthetic analogues<br>T4551: 0.880. Poisoning by, adverse effect of and underdosing of anticoagulants	T8285: 0.898. Stenosis due to cardiac and vascular prosthetic devices<br>T36: 0.872. Poisoning by, adverse effect of and underdosing of systemic antibiotics
XX: External Causes of Morbidity and Mortality. 0.798	**V422: 0.955. Person on outside of car injured in collision with two- or three-wheeled motor vehicle in nontraffic accident**<br>**V433: 0.9281. Unspecified car occupant injured in collision with car, pick-up truck or van in nontraffic accident**<br>**V462: 0.900. Person on outside of car injured in collision with other nonmotor vehicle in nontraffic accident**	**V850: 0.949. Driver of special construction vehicle injured in traffic accident**<br>**V667: 0.919. Person on outside of heavy transport vehicle injured in collision with other nonmotor vehicle in traffic accident**<br>Y830: 0.8799. Surgical operation with transplant of whole organ as the cause of abnormal reaction of the patient, or of later complication, without mention of misadventure at the time of the procedure
XXI: Health Status and Services. 0.7928	**Z998: 0.946. Dependence on other enabling machines and devices**<br>**Z4502: 0.941. Encounter for adjustment and management of automatic implantable cardiac defibrillator**<br>**Z590: 0.923. Homelessness**	**Z681: 0.944. Body mass index (BMI) 19.9 or less, adult**<br>**Z950: 0.940. Presence of cardiac pacemaker**<br>**Z515: 0.907. Encounter for palliative care**

Table A.4: T(ED2ALL)-E(ED2ALL) model (extended version of Tab. A.4: Best-performing individual statements organized according to ICD chapters underscoring the breadth of accurately predictable statements. The table shows the six best-performing individual statements per ICD chapter (10 for chapter IX (Circulatory system diseases)), where we show only AUROC scores above 0.8. To showcase the breadth of reliably predictable statements, we list only the best-performing statement per 3-digit ICD code. Statements with an AUROC score of 0.9 or higher are marked in boldface.

Chapter	Code	Coverage	Prevalence	Description
II	C79	1/8	0.015	Secondary malignant neoplasm of other and unspecified sites
II	C92	1/2	0.002	Myeloid leukemia
III	**D65**	1/1	0.001	Disseminated intravascular coagulation [defibrination syndrome]
IV	E11	2/18	0.217	Type 2 diabetes mellitus
IV	E66	2/4	0.062	Overweight and obesity
IX	I25	3/10	0.162	Chronic ischemic heart disease
IX	**I48**	6/7	0.152	Atrial fibrillation and flutter
IX	I50	9/15	0.142	Heart failure
IX	I27	3/5	0.037	Other pulmonary heart diseases
IX	**I21**	6/8	0.036	Acute myocardial infarction
IX	**I13**	3/3	0.025	Hypertensive heart and chronic kidney disease
IX	**I11**	2/2	0.022	Hypertensive heart disease
IX	I34	1/3	0.020	Nonrheumatic mitral valve disorders
IX	I42	1/3	0.018	Cardiomyopathy
IX	**I44**	5/5	0.015	Atrioventricular and left bundle-branch block
IX	I45	2/5	0.013	Other conduction disorders
IX	I70	1/5	0.008	Atherosclerosis
IX	I08	1/3	0.008	Multiple valve diseases
IX	I31	2/4	0.007	Other diseases of pericardium
IX	I07	1/2	0.005	Rheumatic tricuspid valve diseases
X	J96	2/10	0.049	Respiratory failure, not elsewhere classified
X	J91	1/3	0.011	Pleural effusion in conditions classified elsewhere
X	**J94**	2/2	0.001	Other pleural conditions
X	**J80**	1/1	0.000	Acute respiratory distress syndrome
XI	K76	2/6	0.023	Other diseases of liver
XI	K31	1/4	0.022	Other diseases of stomach and duodenum
XI	**K70**	4/5	0.016	Alcoholic liver disease
XI	**K72**	6/6	0.006	Hepatic failure, not elsewhere classified
XVIII	R57	2/4	0.011	Shock, not elsewhere classified
XIX	T86	2/3	0.004	Complications of transplanted organs and tissue
XX	V43	1/4	0.014	Car occupant injured in collision with car, pick-up truck or van
XX	V46	1/2	0.011	Car occupant injured in collision with other nonmotor vehicle
XX	V42	2/3	0.007	Car occupant injured in collision with two- or three-wheeled motor vehicle
XX	**V66**	2/2	0.006	Occupant of heavy transport vehicle injured in collision with other nonmotor vehicle
XX	**V85**	2/2	0.005	Occupant of special construction vehicle injured in transport accident
XXI	Z95	5/7	0.084	Presence of cardiac and vascular implants and grafts
XXI	Z68	2/8	0.060	Body mass index (BMI)
XXI	Z99	2/5	0.033	Dependence on enabling machines and devices, not elsewhere classified
XXI	Z51	1/2	0.010	Encounter for other aftercare and medical care
XXI	**Z45**	4/4	0.005	Encounter for adjustment and management of implanted device
XXI	Z59	1/2	0.005	Problems related to housing and economic circumstances

Table A.5: T(ED2ALL)-E(ED2ALL) model: Statements with AUROCs larger than 0.9. The statements are sorted by chapter and prevalence in the dataset. Coverage refers to the fraction of codes within this 3-digit category that exceed the specified threshold of 0.9. Categories in boldface correspond to categories with a coverage of 75% or higher, i.e., a situation where the category and 75% of the subcategories are covered with AUROC scores above 0.9.

Chapter	Code	Coverage	Prevalence	Description
I	**A41**	7/7	0.038	Other sepsis
I	B96	2/9	0.036	Other bacterial agents as the cause of diseases classified elsewhere
I	A04	1/2	0.014	Other bacterial intestinal infections
I	B37	2/4	0.011	Candidiasis
I	**A40**	1/1	0.002	Streptococcal sepsis
II	**C79**	7/8	0.015	Secondary malignant neoplasm of other and unspecified sites
II	C78	3/5	0.014	Secondary malignant neoplasm of respiratory and digestive organs
II	**C34**	3/4	0.012	Malignant neoplasm of bronchus and lung
II	**C61**	1/1	0.005	Malignant neoplasm of prostate
II	**C25**	1/1	0.005	Malignant neoplasm of pancreas
II	**D46**	2/2	0.003	Myelodysplastic syndromes
II	C85	2/3	0.002	Other specified and unspecified types of non-Hodgkin lymphoma
II	**C92**	2/2	0.002	Myeloid leukemia
III	**D63**	3/4	0.041	Anemia in chronic diseases classified elsewhere
III	D68	2/4	0.024	Other coagulation defects
III	**D61**	3/3	0.012	Other aplastic anemias and other bone marrow failure syndromes
III	**D65**	1/1	0.001	Disseminated intravascular coagulation [defibrination syndrome]
IV	E78	1/4	0.285	Disorders of lipoprotein metabolism and other lipidemias
IV	E11	12/18	0.217	Type 2 diabetes mellitus
IV	E87	5/11	0.145	Other disorders of fluid, electrolyte and acid-base balance
IV	**E66**	3/4	0.062	Overweight and obesity
IV	E83	3/9	0.029	Disorders of mineral metabolism
IV	E88	2/5	0.025	Other and unspecified metabolic disorders
IV	**E10**	6/7	0.018	Type 1 diabetes mellitus
IV	**E13**	3/3	0.014	Other specified diabetes mellitus
IV	**E43**	1/1	0.012	Unspecified severe protein-calorie malnutrition
IV	**E46**	1/1	0.011	Unspecified protein-calorie malnutrition
IV	**E16**	2/2	0.004	Other disorders of pancreatic internal secretion
IV	E44	1/2	0.004	Protein-calorie malnutrition of moderate and mild degree
V	F17	1/4	0.076	Nicotine dependence
V	F10	3/8	0.059	Alcohol related disorders
V	**F03**	3/3	0.027	Unspecified dementia
V	F43	2/5	0.017	Reaction to severe stress, and adjustment disorders
V	**F02**	3/3	0.014	Dementia in other diseases classified elsewhere
V	**F11**	5/5	0.013	Opioid-related disorders
V	**F14**	3/3	0.012	Cocaine related disorders
V	**F01**	3/3	0.005	Vascular dementia
V	F34	1/2	0.002	Persistent mood [affective] disorders
VI	G89	1/5	0.044	Pain, not elsewhere classified
VI	G93	1/7	0.033	Other disorders of brain
VI	**G30**	2/2	0.010	Alzheimer's disease
VI	G60	1/2	0.008	Hereditary and idiopathic neuropathy
VI	**G20**	1/1	0.008	Parkinson's disease
VI	**G62**	2/2	0.007	Other and unspecified polyneuropathies
VI	**G31**	2/2	0.007	Other degenerative diseases of nervous system, not elsewhere classified

Table A.6: T(ED2ALL)-E(ED2ALL) model: Statements with AUROCs larger than 0.8 (Part 1). The statements are sorted by chapter and prevalence in the dataset. Coverage refers to the fraction of codes within this 3-digit category that exceed the specified threshold of 0.8. Categories in boldface correspond to categories with a coverage of 75% or higher, i.e., a situation where the category and 75% of the subcategories are covered with AUROC scores above 0.8.

Chapter	Code	Coverage	Prevalence	Description
VIII	H91	1/3	0.008	Other and unspecified hearing loss
IX	**I25**	9/10	0.162	Chronic ischemic heart disease
IX	**I48**	7/7	0.152	Atrial fibrillation and flutter
IX	**I50**	15/15	0.142	Heart failure
IX	I12	2/3	0.090	Hypertensive chronic kidney disease
IX	I95	2/7	0.055	Hypotension
IX	**I27**	5/5	0.037	Other pulmonary heart diseases
IX	**I21**	8/8	0.036	Acute myocardial infarction
IX	**I13**	3/3	0.025	Hypertensive heart and chronic kidney disease
IX	**I11**	2/2	0.022	Hypertensive heart disease
IX	**I34**	3/3	0.020	Nonrheumatic mitral valve disorders
IX	**I42**	3/3	0.018	Cardiomyopathy
IX	**I47**	3/3	0.018	Paroxysmal tachycardia
IX	**I44**	5/5	0.015	Atrioventricular and left bundle-branch block
IX	I69	4/6	0.015	Sequelae of cerebrovascular disease
IX	I82	1/5	0.014	Other venous embolism and thrombosis
IX	I63	1/5	0.013	Cerebral infarction
IX	I35	2/3	0.013	Nonrheumatic aortic valve disorders
IX	**I45**	5/5	0.013	Other conduction disorders
IX	I67	1/3	0.012	Other cerebrovascular diseases
IX	**I24**	2/2	0.009	Other acute ischemic heart diseases
IX	I20	2/3	0.008	Angina pectoris
IX	**I70**	4/5	0.008	Atherosclerosis
IX	**I08**	3/3	0.008	Multiple valve diseases
IX	I51	1/2	0.007	Complications and ill-defined descriptions of heart disease
IX	**I31**	4/4	0.007	Other diseases of pericardium
IX	**I85**	3/3	0.006	Esophageal varices
IX	I65	1/3	0.006	Occlusion and stenosis of precerebral arteries, not resulting in cerebral infarction
IX	**I07**	2/2	0.005	Rheumatic tricuspid valve diseases
IX	**I46**	2/2	0.004	Cardiac arrest
IX	**I16**	1/1	0.003	Hypertensive crisis
X	J45	1/4	0.068	Asthma
X	J44	2/3	0.066	Other chronic obstructive pulmonary disease
X	**J96**	10/10	0.049	Respiratory failure, not elsewhere classified
X	J98	3/7	0.018	Other respiratory disorders
X	**J91**	3/3	0.011	Pleural effusion in conditions classified elsewhere
X	**J84**	3/3	0.008	Other interstitial pulmonary diseases
X	J15	1/4	0.007	Bacterial pneumonia, not elsewhere classified
X	J95	2/4	0.006	Intraoperative and postprocedural complications and disorders of respiratory system, not elsewhere classified
X	**J90**	1/1	0.006	Pleural effusion, not elsewhere classified
X	**J47**	1/1	0.003	Bronchiectasis
X	**J94**	2/2	0.001	Other pleural conditions
X	**J80**	1/1	0.000	Acute respiratory distress syndrome
XI	**K76**	6/6	0.023	Other diseases of liver
XI	**K31**	4/4	0.022	Other diseases of stomach and duodenum
XI	**K70**	5/5	0.016	Alcoholic liver disease
XI	K83	2/3	0.010	Other diseases of biliary tract
XI	**K72**	6/6	0.006	Hepatic failure, not elsewhere classified
XI	K65	1/2	0.004	Peritonitis

Table A.7: T(ED2ALL)-E(ED2ALL) model: Statements with AUROCs larger than 0.8 (Part 2). The statements are sorted by chapter and prevalence in the dataset. Coverage refers to the fraction of codes within this 3-digit category that exceed the specified threshold of 0.8. Categories in boldface correspond to categories with a coverage of 75% or higher, i.e., a situation where the category and 75% of the subcategories are covered with AUROC scores above 0.8.

Chapter	Code	Coverage	Prevalence	Description
XII	L03	2/5	0.016	Cellulitis and acute lymphangitis
XII	**L89**	3/3	0.013	Pressure ulcer
XII	**L97**	4/5	0.010	Non-pressure chronic ulcer of lower limb, not elsewhere classified
XIII	**M10**	2/2	0.034	Gout
XIII	M94	1/2	0.011	Other disorders of cartilage
XIII	M35	1/2	0.005	Other systemic involvement of connective tissue
XIII	**M85**	2/2	0.004	Other disorders of bone density and structure
XIII	M32	1/2	0.003	Systemic lupus erythematosus (SLE)
XIII	**M12**	2/2	0.002	Other and unspecified arthropathy
XIV	N18	4/7	0.131	Chronic kidney disease (CKD)
XIV	N17	1/3	0.121	Acute kidney failure
XIV	**N40**	3/3	0.044	Benign prostatic hyperplasia
XIV	**N08**	1/1	0.006	Glomerular disorders in diseases classified elsewhere
XIV	N99	2/3	0.004	Intraoperative and postprocedural complications and disorders of genitourinary system, not elsewhere classified
XVII	**Q23**	1/1	0.001	Congenital malformations of aortic and mitral valves
XVIII	R00	1/4	0.070	Abnormalities of heart beat
XVIII	R53	1/4	0.030	Malaise and fatigue
XVIII	**R65**	4/4	0.027	Symptoms and signs specifically associated with systemic inflammation and infection
XVIII	R41	1/4	0.025	Other symptoms and signs involving cognitive functions and awareness
XVIII	R33	1/3	0.023	Retention of urine
XVIII	R13	1/4	0.021	Aphagia and dysphagia
XVIII	**R57**	4/4	0.011	Shock, not elsewhere classified
XVIII	R40	2/3	0.010	Somnolence, stupor and coma
XVIII	**R18**	2/2	0.010	Ascites
XVIII	**R64**	1/1	0.007	Cachexia
XVIII	**R34**	1/1	0.002	Anuria and oliguria
XIX	T82	4/7	0.011	Complications of cardiac and vascular prosthetic devices, implants and grafts
XIX	**T45**	4/4	0.011	Poisoning by, adverse effect of and underdosing of primarily systemic and hematological agents, not elsewhere classified
XIX	S72	1/2	0.010	Fracture of femur
XIX	T40	1/2	0.008	Poisoning by, adverse effect of and underdosing of narcotics and psychodysleptics [hallucinogens]
XIX	**T50**	1/1	0.008	Poisoning by, adverse effect of and underdosing of diuretics and other and unspecified drugs, medicaments and biological substances
XIX	T81	2/5	0.007	Complications of procedures, not elsewhere classified
XIX	**T38**	2/2	0.005	Poisoning by, adverse effect of and underdosing of hormones and their synthetic substitutes and antagonists, not elsewhere classified
XIX	**T86**	3/3	0.004	Complications of transplanted organs and tissue
XIX	**T36**	1/1	0.004	Poisoning by, adverse effect of and underdosing of systemic antibiotics

Table A.8: T(ED2ALL)-E(ED2ALL) model: Statements with AUROCs larger than 0.8 (Part 3). The statements are sorted by chapter and prevalence in the dataset. Coverage refers to the fraction of codes within this 3-digit category that exceed the specified threshold of 0.8. Categories in boldface correspond to categories with a coverage of 75% or higher, i.e., a situation where the category and 75% of the subcategories are covered with AUROC scores above 0.8.

Chapter	Code	Coverage	Prevalence	Description
XX	Y92	4/12	0.122	Place of occurrence of the external cause
XX	Y83	1/6	0.031	Surgical operation and other surgical procedures as the cause of abnormal reaction of the patient, or of later complication, without mention of misadventure at the time of the procedure
XX	W18	2/5	0.029	Other slipping, tripping and stumbling and falls
XX	Y84	1/4	0.024	Other medical procedures as the cause of abnormal reaction of the patient, or of later complication, without mention of misadventure at the time of the procedure
XX	**V43**	4/4	0.014	Car occupant injured in collision with car, pick-up truck or van
XX	**V17**	2/2	0.013	Pedal cycle rider injured in collision with fixed or stationary object
XX	**V15**	2/2	0.012	Pedal cycle rider injured in collision with railway train or railway vehicle
XX	**V46**	2/2	0.011	Car occupant injured in collision with other nonmotor vehicle
XX	**V10**	2/2	0.010	Pedal cycle rider injured in collision with pedestrian or animal
XX	**V42**	3/3	0.007	Car occupant injured in collision with two- or three-wheeled motor vehicle
XX	**V66**	2/2	0.006	Occupant of heavy transport vehicle injured in collision with other nonmotor vehicle
XX	**V44**	1/1	0.005	Car occupant injured in collision with heavy transport vehicle or bus
XX	**V60**	2/2	0.005	Occupant of heavy transport vehicle injured in collision with pedestrian or animal
XX	**V85**	2/2	0.005	Occupant of special construction vehicle injured in transport accident
XX	**V45**	2/2	0.002	Car occupant injured in collision with railway train or railway vehicle
XX	**V64**	1/1	0.000	Occupant of heavy transport vehicle injured in collision with heavy transport vehicle or bus
XXI	Z79	6/11	0.237	Long term (current) drug therapy
XXI	Z85	2/15	0.093	Personal history of malignant neoplasm
XXI	**Z95**	7/7	0.084	Presence of cardiac and vascular implants and grafts
XXI	**Z66**	1/1	0.062	Do not resuscitate
XXI	**Z68**	8/8	0.060	Body mass index [BMI]
XXI	Z91	1/6	0.049	Personal risk factors, not elsewhere classified
XXI	Z98	3/6	0.045	Other postprocedural states
XXI	**Z99**	5/5	0.033	Dependence on enabling machines and devices, not elsewhere classified
XXI	Z90	2/9	0.028	Acquired absence of organs, not elsewhere classified
XXI	Z96	1/4	0.027	Presence of other functional implants
XXI	Z92	1/4	0.018	Personal history of medical treatment
XXI	**Z51**	2/2	0.010	Encounter for other aftercare and medical care
XXI	**Z89**	5/5	0.009	Acquired absence of limb
XXI	**Z72**	2/2	0.007	Problems related to lifestyle
XXI	Z94	2/3	0.006	Transplanted organ and tissue status
XXI	**Z00**	2/2	0.005	Encounter for general examination without complaint, suspected or reported diagnosis
XXI	**Z45**	4/4	0.005	Encounter for adjustment and management of implanted device
XXI	**Z59**	2/2	0.005	Problems related to housing and economic circumstances
XXI	**Z93**	1/1	0.005	Artificial opening status
XXI	**Z76**	3/3	0.004	Persons encountering health services in other circumstances

Table A.9: T(ED2ALL)-E(ED2ALL) model: Statements with AUROCs larger than 0.8 (Part 4). The statements are sorted by chapter and prevalence in the dataset. Coverage refers to the fraction of codes within this 3-digit category that exceed the specified threshold of 0.8. Categories in boldface correspond to categories with a coverage of 75% or higher, i.e., a situation where the category and 75% of the subcategories are covered with AUROC scores above 0.8.

Chapter	Code	Coverage	Prevalence	Description
I	B95	1/6	0.017	Streptococcus, Staphylococcus, and Enterococcus as the cause of diseases classified elsewhere
I	**B97**	1/1	0.004	Viral agents as the cause of diseases classified elsewhere
I	**B00**	1/1	0.001	Herpesviral [herpes simplex] infections
II	D47	1/3	0.008	Other neoplasms of uncertain behavior of lymphoid, hematopoietic and related tissue
III	**D72**	5/5	0.035	Other disorders of white blood cells
III	D50	2/3	0.027	Iron deficiency anemia
III	D70	1/2	0.004	Neutropenia
III	**D86**	1/1	0.002	Sarcoidosis
III	**D75**	1/1	0.001	Other and unspecified diseases of blood and blood-forming organs
IV	E78	1/4	0.285	Disorders of lipoprotein metabolism and other lipidemias
IV	**E86**	3/4	0.057	Volume depletion
IV	**E55**	2/2	0.011	Vitamin D deficiency
IV	**E04**	2/2	0.007	Other nontoxic goiter
IV	E53	1/2	0.007	Deficiency of other B group vitamins
IV	**E89**	2/2	0.006	Postprocedural endocrine and metabolic complications and disorders, not elsewhere classified
IV	**E05**	1/1	0.006	Thyrotoxicosis [hyperthyroidism]
IV	**E21**	2/2	0.004	Hyperparathyroidism and other disorders of parathyroid gland
V	**F32**	2/2	0.116	Major depressive disorder, single episode
V	**F41**	3/3	0.090	Other anxiety disorders
V	**F31**	2/2	0.023	Bipolar disorder
V	F43	1/5	0.017	Reaction to severe stress, and adjustment disorders
V	**F25**	2/2	0.006	Schizoaffective disorders
V	F06	1/2	0.001	Other mental disorders due to known physiological condition
VI	G47	2/6	0.080	Sleep disorders
VI	G89	3/5	0.044	Pain, not elsewhere classified
VI	G93	1/7	0.033	Other disorders of brain
VI	**G40**	3/3	0.026	Epilepsy and recurrent seizures
VI	**G43**	3/3	0.015	Migraine
VI	**G25**	3/3	0.010	Other extrapyramidal and movement disorders
VI	**G81**	2/2	0.008	Hemiplegia and hemiparesis
VI	**G91**	1/1	0.003	Hydrocephalus
VII	**H53**	1/1	0.010	Visual disturbances
VII	**H26**	2/2	0.003	Other cataract
VIII	**H81**	1/1	0.007	Disorders of vestibular function
IX	I95	2/7	0.055	Hypotension
IX	I67	1/3	0.012	Other cerebrovascular diseases
IX	**I77**	1/1	0.005	Other disorders of arteries and arterioles
IX	I80	1/2	0.001	Phlebitis and thrombophlebitis

Table A.10: T(ED2ALL)-E(ED2ALL) model: Statements with AUROCs smaller than 0.7 (Part 1). The statements are sorted by chapter and prevalence in the dataset. Coverage refers to the fraction of codes within this 3-digit category that stay below the specified threshold of 0.7. Categories in boldface correspond to categories with a coverage of 75% or higher, i.e., a situation where the category and 75% of the subcategories are covered with AUROC scores below 0.7.

Chapter	Code	Coverage	Prevalence	Description
X	J45	2/4	0.068	Asthma
X	**J06**	2/2	0.010	Acute upper respiratory infections of multiple and unspecified sites
X	**J38**	1/1	0.002	Diseases of vocal cords and larynx, not elsewhere classified
XI	**K21**	2/2	0.142	Gastro-esophageal reflux disease
XI	**K59**	4/4	0.042	Other functional intestinal disorders
XI	K92	1/4	0.021	Other diseases of digestive system
XI	**K22**	3/3	0.017	Other diseases of esophagus
XI	K29	1/3	0.014	Gastritis and duodenitis
XI	**K56**	3/4	0.012	Paralytic ileus and intestinal obstruction without hernia
XI	**K86**	2/2	0.010	Other diseases of pancreas
XI	**K80**	3/3	0.010	Cholelithiasis
XI	**K52**	2/2	0.009	Other and unspecified noninfective gastroenteritis and colitis
XI	**K58**	2/2	0.008	Irritable bowel syndrome
XI	**K85**	2/2	0.008	Acute pancreatitis
XI	**K50**	3/3	0.007	Crohn's disease [regional enteritis]
XI	**K62**	1/1	0.006	Other diseases of anus and rectum
XI	**K81**	1/1	0.002	Cholecystitis
XI	**K43**	1/1	0.002	Ventral hernia
XI	**K51**	1/1	0.002	Ulcerative colitis
XI	**K66**	2/2	0.002	Other disorders of peritoneum
XI	**K12**	2/2	0.001	Stomatitis and related lesions
XII	L27	1/2	0.004	Dermatitis due to substances taken internally
XII	**L98**	1/1	0.003	Other disorders of skin and subcutaneous tissue, not elsewhere classified
XIII	**M54**	4/4	0.049	Dorsalgia
XIII	**M79**	6/6	0.036	Other and unspecified soft tissue disorders, not elsewhere classified
XIII	**M25**	5/5	0.024	Other joint disorder, not elsewhere classified
XIII	**M06**	2/2	0.014	Other rheumatoid arthritis
XIII	**M48**	3/4	0.013	Other spondylopathies
XIII	M62	2/3	0.011	Other disorders of muscle
XIII	M47	2/3	0.009	Spondylosis
XIII	M17	1/2	0.007	Osteoarthritis of knee
XIII	**M51**	1/1	0.004	Thoracic, thoracolumbar, and lumbosacral intervertebral disc disorders
XIV	**N28**	2/2	0.006	Other disorders of kidney and ureter, not elsewhere classified
XIV	**N20**	2/2	0.004	Calculus of kidney and ureter
XIV	**N31**	2/2	0.003	Neuromuscular dysfunction of bladder, not elsewhere classified
XVII	**Q21**	2/2	0.003	Congenital malformations of cardiac septa

Table A.11: T(ED2ALL)-E(ED2ALL) model: Statements with AUROCs smaller than 0.7 (Part 2). The statements are sorted by chapter and prevalence in the dataset. Coverage refers to the fraction of codes within this 3-digit category that stay below the specified threshold of 0.7. Categories in boldface correspond to categories with a coverage of 75% or higher, i.e., a situation where the category and 75% of the subcategories are covered with AUROC scores below 0.7.

Chapter	Code	Coverage	Prevalence	Description
XVIII	**R07**	3/4	0.191	Pain in throat and chest
XVIII	R00	1/4	0.070	Abnormalities of heart beat
XVIII	**R10**	7/7	0.068	Abdominal and pelvic pain
XVIII	R06	3/6	0.053	Abnormalities of breathing
XVIII	**R55**	1/1	0.048	Syncope and collapse
XVIII	**R11**	4/4	0.043	Nausea and vomiting
XVIII	**R42**	1/1	0.043	Dizziness and giddiness
XVIII	**R53**	3/4	0.030	Malaise and fatigue
XVIII	**R51**	1/1	0.030	Headache
XVIII	**R19**	2/2	0.029	Other symptoms and signs involving the digestive system and abdomen
XVIII	**R41**	3/4	0.025	Other symptoms and signs involving cognitive functions and awareness
XVIII	R79	1/4	0.023	Other abnormal findings of blood chemistry
XVIII	**R45**	3/4	0.022	Symptoms and signs involving emotional state
XVIII	**R05**	1/1	0.020	Cough
XVIII	R50	1/4	0.020	Fever of other and unknown origin
XVIII	R74	1/3	0.019	Abnormal serum enzyme levels
XVIII	**R47**	4/4	0.018	Speech disturbances, not elsewhere classified
XVIII	R91	2/3	0.016	Abnormal findings on diagnostic imaging of lung
XVIII	**R29**	3/4	0.015	Other symptoms and signs involving the nervous and musculoskeletal systems
XVIII	R20	1/2	0.014	Disturbances of skin sensation
XVIII	R60	1/2	0.014	Edema, not elsewhere classified
XVIII	R94	1/3	0.011	Abnormal results of function studies
XVIII	**R56**	2/2	0.010	Convulsions, not elsewhere classified
XVIII	R04	1/2	0.010	Hemorrhage from respiratory passages
XVIII	R40	1/3	0.010	Somnolence, stupor and coma
XVIII	**R73**	3/3	0.008	Elevated blood glucose level
XVIII	R78	1/3	0.008	Findings of drugs and other substances, not normally found in blood
XVIII	**R25**	1/1	0.005	Abnormal involuntary movements
XVIII	**R21**	1/1	0.005	Rash and other nonspecific skin eruption
XVIII	**R68**	1/1	0.004	Other general symptoms and signs
XVIII	**R59**	1/1	0.003	Enlarged lymph nodes
XIX	S06	1/3	0.014	Intracranial injury
XIX	T82	1/7	0.011	Complications of cardiac and vascular prosthetic devices, implants and grafts
XIX	**S22**	2/2	0.010	Fracture of rib(s), sternum and thoracic spine
XIX	**S82**	1/1	0.005	Fracture of lower leg, including ankle
XIX	**S42**	1/1	0.005	Fracture of shoulder and upper arm
XIX	**T84**	1/1	0.002	Complications of internal orthopedic prosthetic devices, implants and grafts
XX	Y92	3/12	0.122	Place of occurrence of the external cause
XX	Y83	4/6	0.031	Surgical operation and other surgical procedures as the cause of abnormal reaction of the patient, or of later complication, without mention of misadventure at the time of the procedure
XX	W18	2/5	0.029	Other slipping, tripping and stumbling and falls
XX	**X58**	1/1	0.011	Exposure to other specified factors
XXI	Z79	1/11	0.237	Long term (current) drug therapy
XXI	Z87	3/7	0.163	Personal history of other diseases and conditions
XXI	Z86	7/10	0.099	Personal history of certain other diseases
XXI	Z85	2/15	0.093	Personal history of malignant neoplasm
XXI	Z91	1/6	0.049	Personal risk factors, not elsewhere classified
XXI	Z98	1/6	0.045	Other postprocedural states
XXI	Z90	1/9	0.028	Acquired absence of organs, not elsewhere classified
XXI	**Z23**	1/1	0.025	Encounter for immunization
XXI	**Z16**	1/1	0.003	Resistance to antimicrobial drugs

Table A.12: T(ED2ALL)-E(ED2ALL) model: Statements with AUROCs smaller than 0.7 (Part 3). The statements are sorted by chapter and prevalence in the dataset. Coverage refers to the fraction of codes within this 3-digit category that stay below the specified threshold of 0.7. Categories in boldface correspond to categories with a coverage of 75% or higher, i.e., a situation where the category and 75% of the subcategories are covered with AUROC scores below 0.7.

Block	Code, AUROC and description	Code, AUROC and description
I: Infectious and Parasitic Diseases	A4151: 0.834. E. coli	A40: 0.830. Streptococcal sepsis
II: Neoplasms (Cancer and Tumors)	C925: 0.874. Acute myelomonocytic leukemia<br>C34: 0.829. Malignant neoplasm of bronchus and lung<br>C25: 0.813. Malignant neoplasm of pancreas	C7952: 0.852. Secondary malignant neoplasm of bone marrow<br>C786: 0.822. Secondary malignant neoplasm of retroperitoneum and peritoneum<br>C22: 0.812. Malignant neoplasm of liver and intrahepatic bile ducts
III: Blood, Immune, and Hematological Disorders	**D65: 0.902. Disseminated intravascular coagulation [defibrination syndrome]**<br>D631: 0.834. Anemia in chronic kidney disease	D684: 0.852. Acquired coagulation factor deficiency<br>D89: 0.831. Other disorders involving the immune mechanism
IV: Endocrine, Nutritional, and Metabolic Diseases	**E660: 0.913. Obesity due to excess calories**<br>E103: 0.878. Type 1 diabetes mellitus with ophthalmic complications<br>E134: 0.839. Other specified diabetes mellitus with neurological complications	E1129: 0.881. Type 2 diabetes mellitus with other diabetic kidney complications<br>E43: 0.864. Unspecified severe protein-calorie malnutrition<br>E7800: 0.820. Pure hypercholesterolemia, unspecified
V: Mental and Behavioral Disorders	F1022: 0.881. Alcohol dependence with intoxication<br>F1410: 0.828. Cocaine abuse, uncomplicated<br>F068: 0.813. Other specified mental disorders due to known physiological conditions	F11: 0.855. Opioid-related disorders<br>F129: 0.826. Cannabis use, unspecified<br>F4310: 0.812. Post-traumatic stress disorder, unspecified
VI: Nervous System Disorders	G931: 0.859. Anoxic brain damage, not elsewhere classified<br>G20: 0.803. Parkinson's disease	G309: 0.807. Alzheimer's disease, unspecified
IX: Circulatory System Diseases	**I2109: 0.983. ST elevation (STEMI) myocardial infarction involving other coronary artery of anterior wall**<br>**I255: 0.949. Ischemic cardiomyopathy**<br>**I4510: 0.939. Unspecified right bundle-branch block**<br>**I481: 0.926. Persistent atrial fibrillation**<br>**I078: 0.909. Other rheumatic tricuspid valve diseases**<br>I2789: 0.897. Other specified pulmonary heart diseases	**I447: 0.959. Left bundle-branch block, unspecified**<br>**I314: 0.947. Cardiac tamponade**<br>**I5023: 0.929. Acute on chronic systolic (congestive) heart failure**<br>**I428: 0.910. Other cardiomyopathies**<br>**I851: 0.901. Secondary esophageal varices**<br>I13: 0.891. Hypertensive heart and chronic kidney disease
X: Respiratory System Diseases	J9621: 0.880. Acute and chronic respiratory failure with hypoxia<br>J948: 0.8534. Other specified pleural conditions<br>J47: 0.8128. Bronchiectasis	J910: 0.860. Malignant pleural effusion<br>J441: 0.826. Chronic obstructive pulmonary disease with (acute) exacerbation<br>J80: 0.803. Acute respiratory distress syndrome
XI: Digestive System Diseases	**K767: 0.953. Hepatorenal syndrome**<br>**K7291: 0.909. Hepatic failure, unspecified with coma**<br>K65: 0.855. Peritonitis	**K701: 0.921. Alcoholic hepatitis**<br>**K3189: 0.904. Other diseases of stomach and duodenum**<br>K830: 0.821. Cholangitis
XII: Skin and Subcutaneous Tissue Diseases	L9740: 0.842. Non-pressure chronic ulcer of unspecified heel and midfoot	L8915: 0.835. Pressure ulcer of sacral region
XIV: Genitourinary System Diseases	N186: 0.869. End-stage renal disease<br>N170: 0.822. Acute kidney failure with tubular necrosis	N08: 0.832. Glomerular disorders in diseases classified elsewhere<br>N401: 0.813. Benign prostatic hyperplasia with lower urinary tract symptoms
XVII: Congenital Abnormalities	Q23: 0.866. Congenital malformations of aortic and mitral valves	
XVIII: Symptoms and Clinical Findings	**R570: 0.916. Cardiogenic shock**<br>R18: 0.876. Ascites<br>R000: 0.831. Tachycardia, unspecified	R64: 0.893. Cachexia<br>R6521: 0.870. Severe sepsis with septic shock<br>R402: 0.819. Coma
XIX: Injuries and External Causes	**T8612: 0.903. Kidney transplant failure**<br>T8289: 0.807. Other specified complications of cardiac and vascular prosthetic devices, implants, and grafts	T811: 0.813. Postprocedural shock<br>T4551: 0.805. Poisoning by, adverse effect of and underdosing of anticoagulants
XX: External Causes of Morbidity and Mortality	**V850: 0.930. Driver of special construction vehicle injured in traffic accident**<br>**V433: 0.904. Unspecified car occupant injured in collision with car, pick-up truck, or van in nontraffic accident**<br>V600: 0.865. Driver of heavy transport vehicle injured in collision with pedestrian or animal in nontraffic accident	**V422: 0.920. Person on outside of car injured in collision with two- or three-wheeled motor vehicle in nontraffic accident**<br>V462: 0.871. Person on outside of car injured in collision with other nonmotor vehicle in nontraffic accident<br>V667: 0.848. Person on outside of heavy transport vehicle injured in collision with other nonmotor vehicle in traffic accident
XXI: Health Status and Services	**Z4502: 0.960. Encounter for adjustment and management of automatic implantable cardiac defibrillator**<br>**Z681: 0.913. Body mass index (BMI) 19.9 or less, adult**<br>Z7682: 0.888. Awaiting organ transplant status	**Z9581: 0.935. Presence of other cardiac implants and grafts**<br>**Z9981: 0.912. Dependence on supplemental oxygen**<br>Z590: 0.882. Homelessness

Table A.13: T(ALL2ALL)-E(ALL2ALL) model: Best-performing individual statements organized according to ICD chapters. The table shows the six best-performing individual statements per ICD chapter (10 for chapter IX), where we show only AUROC scores above 0.8, see also Tab. A.14, Tab. A.15-Tab. A.17, and Tab. A.18-Tab. A.20 for a summary corresponding summary of ICD codes at 3-digit level with AUROC scores above 0.9, 0.8 and below 0.7, respectively. To showcase the breadth of reliably predictable statements by listing only the best-performing statement per 3-digit ICD code. Statements with AUROC score of 0.9 or higher are marked in boldface.

Chapter	Code	Coverage	Prevalence	Description
III	**D65**	1/1	0.002	Disseminated intravascular coagulation [defibrination syndrome]
IV	E66	2/4	0.081	Overweight and obesity
IX	I25	2/10	0.229	Chronic ischemic heart disease
IX	I48	3/7	0.204	Atrial fibrillation and flutter
IX	I50	4/15	0.194	Heart failure
IX	I21	4/8	0.054	Acute myocardial infarction
IX	I42	1/3	0.028	Cardiomyopathy
IX	**I44**	4/5	0.024	Atrioventricular and left bundle-branch block
IX	I45	2/5	0.015	Other conduction disorders
IX	I31	1/4	0.011	Other diseases of pericardium
IX	I07	1/2	0.009	Rheumatic tricuspid valve diseases
IX	I85	1/3	0.009	Esophageal varices
XI	K76	1/6	0.032	Other diseases of liver
XI	K31	1/4	0.027	Other diseases of stomach and duodenum
XI	K70	2/5	0.019	Alcoholic liver disease
XI	K72	1/6	0.009	Hepatic failure, not elsewhere classified
XVIII	R57	1/4	0.015	Shock, not elsewhere classified
XIX	T86	1/3	0.010	Complications of transplanted organs and tissue
XX	V43	1/4	0.020	Car occupant injured in collision with car, pick-up truck or van
XX	V42	1/3	0.010	Car occupant injured in collision with two- or three-wheeled motor vehicle
XX	V85	1/2	0.008	Occupant of special construction vehicle injured in transport accident
XXI	Z95	4/7	0.106	Presence of cardiac and vascular implants and grafts
XXI	Z68	2/8	0.072	Body mass index [BMI]
XXI	Z99	2/5	0.036	Dependence on enabling machines and devices, not elsewhere classified
XXI	**Z45**	3/4	0.007	Encounter for adjustment and management of implanted device

Table A.14: T(ALL2ALL)-E(ALL2ALL) model: Statements with AUROCs larger than 0.9. The statements are sorted by chapter and prevalence in the dataset. Coverage refers to the fraction of codes within this 3-digit category that exceed the specified threshold of 0.9. Categories in boldface correspond to categories with a coverage of 75% or higher, i.e., a situation where the category and 75% of the subcategories are covered with AUROC scores above 0.9.

Chapter	Code	Coverage	Prevalence	Description
I	A41	4/7	0.049	Other sepsis
I	**A40**	1/1	0.003	Streptococcal sepsis
II	C79	3/8	0.019	Secondary malignant neoplasm of other and unspecified sites
II	C78	2/5	0.018	Secondary malignant neoplasm of respiratory and digestive organs
II	C34	1/4	0.014	Malignant neoplasm of bronchus and lung
II	**C25**	1/1	0.005	Malignant neoplasm of pancreas
II	**C92**	2/2	0.005	Myeloid leukemia
II	**C22**	1/1	0.005	Malignant neoplasm of liver and intrahepatic bile ducts
III	D63	2/4	0.052	Anemia in chronic diseases classified elsewhere
III	D68	2/4	0.032	Other coagulation defects
III	**D89**	1/1	0.004	Other disorders involving the immune mechanism, not elsewhere classified
III	**D65**	1/1	0.002	Disseminated intravascular coagulation [defibrination syndrome]
IV	E78	1/4	0.353	Disorders of lipoprotein metabolism and other lipidemias
IV	E11	10/18	0.248	Type 2 diabetes mellitus
IV	E87	1/11	0.186	Other disorders of fluid, electrolyte and acid-base balance
IV	**E66**	3/4	0.081	Overweight and obesity
IV	**E13**	3/3	0.024	Other specified diabetes mellitus
IV	E10	5/7	0.022	Type 1 diabetes mellitus
IV	**E43**	1/1	0.014	Unspecified severe protein-calorie malnutrition
V	F10	4/8	0.061	Alcohol related disorders
V	F43	1/5	0.020	Reaction to severe stress, and adjustment disorders
V	F02	1/3	0.015	Dementia in other diseases classified elsewhere
V	**F11**	5/5	0.014	Opioid related disorders
V	**F14**	3/3	0.012	Cocaine related disorders
V	**F12**	2/2	0.009	Cannabis related disorders
V	F06	1/2	0.006	Other mental disorders due to known physiological condition
VI	G93	1/7	0.041	Other disorders of brain
VI	**G30**	2/2	0.011	Alzheimer's disease
VI	**G20**	1/1	0.008	Parkinson's disease
IX	**I25**	9/10	0.229	Chronic ischemic heart disease
IX	**I48**	7/7	0.204	Atrial fibrillation and flutter
IX	**I50**	13/15	0.194	Heart failure
IX	I12	1/3	0.124	Hypertensive chronic kidney disease
IX	**I21**	8/8	0.054	Acute myocardial infarction
IX	**I27**	5/5	0.050	Other pulmonary heart diseases
IX	**I34**	3/3	0.031	Nonrheumatic mitral valve disorders
IX	**I42**	3/3	0.028	Cardiomyopathy
IX	I47	1/3	0.028	Paroxysmal tachycardia
IX	**I13**	3/3	0.027	Hypertensive heart and chronic kidney disease
IX	**I35**	3/3	0.025	Nonrheumatic aortic valve disorders
IX	**I44**	5/5	0.024	Atrioventricular and left bundle-branch block
IX	**I11**	2/2	0.024	Hypertensive heart disease
IX	**I70**	4/5	0.023	Atherosclerosis
IX	I20	2/3	0.020	Angina pectoris
IX	I69	2/6	0.020	Sequelae of cerebrovascular disease
IX	I71	1/3	0.020	Aortic aneurysm and dissection
IX	**I45**	5/5	0.015	Other conduction disorders
IX	I97	1/3	0.014	Intraoperative and postprocedural complications and disorders of circulatory system, not elsewhere classified

Table A.15: T(ALL2ALL)-E(ALL2ALL) model: Statements with AUROCs larger than 0.8 (Part 1). The statements are sorted by chapter and prevalence in the dataset. Coverage refers to the fraction of codes within this 3-digit category that exceed the specified threshold of 0.8. Categories in boldface correspond to categories with a coverage of 75% or higher, i.e., a situation where the category and 75% of the subcategories are covered with AUROC scores above 0.8.

Chapter	Code	Coverage	Prevalence	Description
IX	**I08**	3/3	0.011	Multiple valve diseases
IX	**I31**	4/4	0.011	Other diseases of pericardium
IX	I65	1/3	0.011	Occlusion and stenosis of precerebral arteries, not resulting in cerebral infarction
IX	**I07**	2/2	0.009	Rheumatic tricuspid valve diseases
IX	**I85**	3/3	0.009	Esophageal varices
IX	I51	1/2	0.009	Complications and ill-defined descriptions of heart disease
IX	**I46**	2/2	0.006	Cardiac arrest
IX	**I16**	1/1	0.003	Hypertensive crisis
IX	**I33**	2/2	0.002	Acute and subacute endocarditis
X	J44	1/3	0.088	Other chronic obstructive pulmonary disease
X	**J96**	8/10	0.067	Respiratory failure, not elsewhere classified
X	**J91**	3/3	0.022	Pleural effusion in conditions classified elsewhere
X	J95	1/4	0.015	Intraoperative and postprocedural complications and disorders of respiratory system, not elsewhere classified
X	J15	1/4	0.011	Bacterial pneumonia, not elsewhere classified
X	**J47**	1/1	0.003	Bronchiectasis
X	**J94**	2/2	0.003	Other pleural conditions
X	**J80**	1/1	0.002	Acute respiratory distress syndrome
XI	K76	4/6	0.032	Other diseases of liver
XI	**K31**	4/4	0.027	Other diseases of stomach and duodenum
XI	**K70**	5/5	0.019	Alcoholic liver disease
XI	K83	1/3	0.013	Other diseases of biliary tract
XI	**K72**	6/6	0.009	Hepatic failure, not elsewhere classified
XI	**K65**	2/2	0.007	Peritonitis
XI	K66	1/2	0.005	Other disorders of peritoneum
XII	**L89**	3/3	0.020	Pressure ulcer
XII	**L97**	4/5	0.020	Non-pressure chronic ulcer of lower limb, not elsewhere classified
XIV	N18	2/7	0.171	Chronic kidney disease (CKD)
XIV	N17	1/3	0.165	Acute kidney failure
XIV	**N40**	3/3	0.057	Benign prostatic hyperplasia
XIV	**N08**	1/1	0.008	Glomerular disorders in diseases classified elsewhere
XVII	**Q23**	1/1	0.003	Congenital malformations of aortic and mitral valves
XVIII	R00	1/4	0.080	Abnormalities of heart beat
XVIII	R06	1/6	0.039	Abnormalities of breathing
XVIII	**R65**	4/4	0.036	Symptoms and signs specifically associated with systemic inflammation and infection
XVIII	R50	1/4	0.025	Fever of other and unknown origin
XVIII	**R57**	3/4	0.015	Shock, not elsewhere classified
XVIII	**R18**	2/2	0.015	Ascites
XVIII	R40	1/3	0.011	Somnolence, stupor and coma
XVIII	**R64**	1/1	0.009	Cachexia
XVIII	**R34**	1/1	0.003	Anuria and oliguria
XIX	T82	1/7	0.024	Complications of cardiac and vascular prosthetic devices, implants and grafts
XIX	T81	1/5	0.014	Complications of procedures, not elsewhere classified
XIX	T45	1/4	0.013	Poisoning by, adverse effect of and underdosing of primarily systemic and hematological agents, not elsewhere classified
XIX	**T86**	3/3	0.010	Complications of transplanted organs and tissue

Table A.16: T(ALL2ALL)-E(ALL2ALL) model: Statements with AUROCs larger than 0.8 (Part 2). The statements are sorted by chapter and prevalence in the dataset. Coverage refers to the fraction of codes within this 3-digit category that exceed the specified threshold of 0.8. Categories in boldface correspond to categories with a coverage of 75% or higher, i.e., a situation where the category and 75% of the subcategories are covered with AUROC scores above 0.8.

Chapter	Code	Coverage	Prevalence	Description
XX	Y83	1/6	0.064	Surgical operation and other surgical procedures as the cause of abnormal reaction of the patient, or of later complication, without mention of misadventure at the time of the procedure
XX	Y84	1/4	0.039	Other medical procedures as the cause of abnormal reaction of the patient, or of later complication, without mention of misadventure at the time of the procedure
XX	V43	2/4	0.020	Car occupant injured in collision with car, pick-up truck or van
XX	**V10**	2/2	0.016	Pedal cycle rider injured in collision with pedestrian or animal
XX	**V46**	2/2	0.012	Car occupant injured in collision with other nonmotor vehicle
XX	**V42**	3/3	0.010	Car occupant injured in collision with two- or three-wheeled motor vehicle
XX	**V85**	2/2	0.008	Occupant of special construction vehicle injured in transport accident
XX	**V66**	2/2	0.008	Occupant of heavy transport vehicle injured in collision with other nonmotor vehicle
XX	**V60**	2/2	0.006	Occupant of heavy transport vehicle injured in collision with pedestrian or animal
XXI	Z79	1/11	0.265	Long term (current) drug therapy
XXI	Z85	1/15	0.119	Personal history of malignant neoplasm
XXI	**Z95**	7/7	0.106	Presence of cardiac and vascular implants and grafts
XXI	Z68	5/8	0.072	Body mass index [BMI]
XXI	Z98	2/6	0.069	Other postprocedural states
XXI	**Z66**	1/1	0.067	Do not resuscitate
XXI	**Z99**	4/5	0.036	Dependence on enabling machines and devices, not elsewhere classified
XXI	Z51	1/2	0.015	Encounter for other aftercare and medical care
XXI	**Z89**	5/5	0.014	Acquired absence of limb
XXI	**Z45**	4/4	0.007	Encounter for adjustment and management of implanted device
XXI	**Z76**	3/3	0.006	Persons encountering health services in other circumstances
XXI	**Z59**	2/2	0.004	Problems related to housing and economic circumstances

Table A.17: T(ALL2ALL)-E(ALL2ALL) model: Statements with AUROCs larger than 0.8 (Part 3). The statements are sorted by chapter and prevalence in the dataset. Coverage refers to the fraction of codes within this 3-digit category that exceed the specified threshold of 0.8. Categories in boldface correspond to categories with a coverage of 75% or higher, i.e., a situation where the category and 75% of the subcategories are covered with AUROC scores above 0.8.

Chapter	Code	Coverage	Prevalence	Description
I	B96	2/9	0.045	Other bacterial agents as the cause of diseases classified elsewhere
I	B95	2/6	0.030	Streptococcus, Staphylococcus, and Enterococcus as the cause of diseases classified elsewhere
I	**A08**	1/1	0.005	Viral and other specified intestinal infections
I	**B00**	1/1	0.001	Herpesviral [herpes simplex] infections
II	D47	2/3	0.011	Other neoplasms of uncertain behavior of lymphoid, hematopoietic and related tissue
II	**C91**	3/3	0.009	Lymphoid leukemia
III	D64	2/3	0.096	Other anemias
III	**D72**	5/5	0.040	Other disorders of white blood cells
III	**D50**	3/3	0.036	Iron deficiency anemia
III	D68	1/4	0.032	Other coagulation defects
III	**D86**	1/1	0.003	Sarcoidosis
III	**D75**	1/1	0.002	Other and unspecified diseases of blood and blood-forming organs
IV	E78	1/4	0.353	Disorders of lipoprotein metabolism and other lipidemias
IV	**E03**	2/2	0.106	Other hypothyroidism
IV	**E86**	3/4	0.066	Volume depletion
IV	E83	1/9	0.036	Disorders of mineral metabolism
IV	**E55**	2/2	0.013	Vitamin D deficiency
IV	**E27**	3/3	0.011	Other disorders of adrenal gland
IV	**E53**	2/2	0.009	Deficiency of other B group vitamins
IV	**E04**	2/2	0.008	Other nontoxic goiter
IV	**E89**	2/2	0.008	Postprocedural endocrine and metabolic complications and disorders, not elsewhere classified
IV	**E22**	2/2	0.008	Hyperfunction of pituitary gland
IV	**E05**	1/1	0.007	Thyrotoxicosis [hyperthyroidism]
IV	**E21**	2/2	0.005	Hyperparathyroidism and other disorders of parathyroid gland
V	**F32**	2/2	0.129	Major depressive disorder, single episode
V	**F41**	3/3	0.098	Other anxiety disorders
V	**F31**	2/2	0.026	Bipolar disorder
V	F43	1/5	0.020	Reaction to severe stress, and adjustment disorders
V	**F25**	2/2	0.007	Schizoaffective disorders
VI	G47	2/6	0.102	Sleep disorders
VI	**G89**	4/5	0.052	Pain, not elsewhere classified
VI	**G40**	3/3	0.032	Epilepsy and recurrent seizures
VI	**G25**	3/3	0.012	Other extrapyramidal and movement disorders
VI	**G81**	2/2	0.008	Hemiplegia and hemiparesis
VI	**G91**	1/1	0.005	Hydrocephalus
VII	**H54**	2/2	0.009	Blindness and low vision
VII	**H53**	1/1	0.009	Visual disturbances
VII	**H26**	2/2	0.004	Other cataract
VIII	**H81**	1/1	0.005	Disorders of vestibular function
IX	I95	3/7	0.075	Hypotension
IX	I67	2/3	0.012	Other cerebrovascular diseases
IX	**I77**	1/1	0.007	Other disorders of arteries and arterioles
IX	**I80**	2/2	0.002	Phlebitis and thrombophlebitis
X	J45	2/4	0.076	Asthma
X	J98	1/7	0.033	Other respiratory disorders
X	**J38**	1/1	0.003	Diseases of vocal cords and larynx, not elsewhere classified

Table A.18: T(ALL2ALL)-E(ALL2ALL) model: Statements with AUROCs smaller than 0.7 (Part 1). The statements are sorted by chapter and prevalence in the dataset. Coverage refers to the fraction of codes within this 3-digit category that stay below the specified threshold of 0.7. Categories in boldface correspond to categories with a coverage of 75% or higher, i.e., a situation where the category and 75% of the subcategories are covered with AUROC scores below 0.7.

Chapter	Code	Coverage	Prevalence	Description
XI	**K21**	2/2	0.176	Gastro-esophageal reflux disease
XI	**K59**	4/4	0.050	Other functional intestinal disorders
XI	K92	1/4	0.024	Other diseases of digestive system
XI	**K57**	3/3	0.021	Diverticular disease of intestine
XI	K56	1/4	0.020	Paralytic ileus and intestinal obstruction without hernia
XI	**K22**	3/3	0.020	Other diseases of esophagus
XI	**K29**	3/3	0.016	Gastritis and duodenitis
XI	**K80**	3/3	0.013	Cholelithiasis
XI	**K86**	2/2	0.013	Other diseases of pancreas
XI	**K58**	2/2	0.011	Irritable bowel syndrome
XI	**K85**	2/2	0.011	Acute pancreatitis
XI	K63	1/2	0.009	Other diseases of intestine
XI	**K64**	2/2	0.009	Hemorrhoids and perianal venous thrombosis
XI	**K52**	2/2	0.009	Other and unspecified noninfective gastroenteritis and colitis
XI	**K50**	3/3	0.009	Crohn's disease [regional enteritis]
XI	**K62**	1/1	0.007	Other diseases of anus and rectum
XI	**K25**	1/1	0.005	Gastric ulcer
XI	**K51**	1/1	0.004	Ulcerative colitis
XI	**K81**	1/1	0.003	Cholecystitis
XII	L03	1/5	0.024	Cellulitis and acute lymphangitis
XII	**L40**	1/1	0.008	Psoriasis
XII	L27	1/2	0.006	Dermatitis due to substances taken internally
XIII	**M54**	4/4	0.048	Dorsalgia
XIII	**M79**	5/6	0.035	Other and unspecified soft tissue disorders, not elsewhere classified
XIII	**M25**	5/5	0.021	Other joint disorder, not elsewhere classified
XIII	M48	2/4	0.016	Other spondylopathies
XIII	**M06**	2/2	0.015	Other rheumatoid arthritis
XIII	**M47**	3/3	0.013	Spondylosis
XIII	M94	1/2	0.013	Other disorders of cartilage
XIII	M62	2/3	0.012	Other disorders of muscle
XIII	M17	1/2	0.008	Osteoarthritis of knee
XIII	**M51**	1/1	0.006	Thoracic, thoracolumbar, and lumbosacral intervertebral disc disorders
XIII	M85	1/2	0.004	Other disorders of bone density and structure
XIV	**N28**	2/2	0.009	Other disorders of kidney and ureter, not elsewhere classified
XIV	**N32**	1/1	0.005	Other disorders of bladder
XIV	**N20**	2/2	0.004	Calculus of kidney and ureter
XVII	**Q21**	2/2	0.004	Congenital malformations of cardiac septa
XVIII	R00	1/4	0.080	Abnormalities of heart beat
XVIII	R10	5/7	0.050	Abdominal and pelvic pain
XVIII	**R11**	4/4	0.041	Nausea and vomiting
XVIII	**R06**	5/6	0.039	Abnormalities of breathing
XVIII	**R55**	1/1	0.039	Syncope and collapse
XVIII	**R19**	2/2	0.032	Other symptoms and signs involving the digestive system and abdomen
XVIII	R33	2/3	0.031	Retention of urine
XVIII	R79	2/4	0.026	Other abnormal findings of blood chemistry
XVIII	R50	1/4	0.025	Fever of other and unknown origin
XVIII	**R51**	1/1	0.024	Headache
XVIII	R13	1/4	0.024	Aphagia and dysphagia
XVIII	**R53**	3/4	0.023	Malaise and fatigue
XVIII	**R41**	3/4	0.022	Other symptoms and signs involving cognitive functions and awareness
XVIII	R45	1/4	0.021	Symptoms and signs involving emotional state
XVIII	**R74**	3/3	0.021	Abnormal serum enzyme levels
XVIII	**R91**	3/3	0.017	Abnormal findings on diagnostic imaging of lung
XVIII	**R47**	4/4	0.017	Speech disturbances, not elsewhere classified
XVIII	**R29**	4/4	0.016	Other symptoms and signs involving the nervous and musculoskeletal systems

Table A.19: T(ALL2ALL)-E(ALL2ALL) model: Statements with AUROCs smaller than 0.7 (Part 2). The statements are sorted by chapter and prevalence in the dataset. Coverage refers to the fraction of codes within this 3-digit category that stay below the specified threshold of 0.7. Categories in boldface correspond to categories with a coverage of 75% or higher, i.e., a situation where the category and 75% of the subcategories are covered with AUROC scores below 0.7.

Chapter	Code	Coverage	Prevalence	Description
XVIII	R31	2/3	0.015	Hematuria
XVIII	**R05**	1/1	0.014	Cough
XVIII	R60	1/2	0.014	Edema, not elsewhere classified
XVIII	R63	1/2	0.014	Symptoms and signs concerning food and fluid intake
XVIII	R94	1/3	0.012	Abnormal results of function studies
XVIII	**R26**	2/2	0.012	Abnormalities of gait and mobility
XVIII	R40	1/3	0.011	Somnolence, stupor and coma
XVIII	**R56**	2/2	0.011	Convulsions, not elsewhere classified
XVIII	R20	1/2	0.011	Disturbances of skin sensation
XVIII	R04	1/2	0.010	Hemorrhage from respiratory passages
XVIII	**R73**	3/3	0.009	Elevated blood glucose level
XVIII	**R82**	1/1	0.006	Other and unspecified abnormal findings in urine
XVIII	**R21**	1/1	0.005	Rash and other nonspecific skin eruption
XVIII	**R25**	1/1	0.005	Abnormal involuntary movements
XVIII	**R68**	1/1	0.004	Other general symptoms and signs
XVIII	**R59**	1/1	0.004	Enlarged lymph nodes
XIX	T82	2/7	0.024	Complications of cardiac and vascular prosthetic devices, implants and grafts
XIX	S06	2/3	0.017	Intracranial injury
XIX	**S22**	2/2	0.011	Fracture of rib(s), sternum and thoracic spine
XIX	**S01**	1/1	0.009	Open wound of head
XIX	**T40**	2/2	0.008	Poisoning by, adverse effect of and underdosing of narcotics and psychodysleptics [hallucinogens]
XIX	**S32**	1/1	0.006	Fracture of lumbar spine and pelvis
XIX	**S42**	1/1	0.006	Fracture of shoulder and upper arm
XIX	**S82**	1/1	0.005	Fracture of lower leg, including ankle
XIX	T88	1/2	0.004	Other complications of surgical and medical care, not elsewhere classified
XX	Y92	7/12	0.138	Place of occurrence of the external cause
XX	Y83	1/6	0.064	Surgical operation and other surgical procedures as the cause of abnormal reaction of the patient, or of later complication, without mention of misadventure at the time of the procedure
XX	Y84	1/4	0.039	Other medical procedures as the cause of abnormal reaction of the patient, or of later complication, without mention of misadventure at the time of the procedure
XX	W18	1/5	0.027	Other slipping, tripping and stumbling and falls
XX	**X58**	1/1	0.010	Exposure to other specified factors
XX	**V64**	1/1	0.002	Occupant of heavy transport vehicle injured in collision with heavy transport vehicle or bus
XXI	Z79	2/11	0.265	Long term (current) drug therapy
XXI	**Z87**	7/7	0.189	Personal history of other diseases and conditions
XXI	Z85	5/15	0.119	Personal history of malignant neoplasm
XXI	**Z86**	8/10	0.116	Personal history of certain other diseases
XXI	Z98	2/6	0.069	Other postprocedural states
XXI	Z91	2/6	0.053	Personal risk factors, not elsewhere classified
XXI	Z90	5/9	0.034	Acquired absence of organs, not elsewhere classified
XXI	**Z23**	1/1	0.027	Encounter for immunization
XXI	Z82	1/3	0.017	Family history of certain disabilities and chronic diseases (leading to disablement)
XXI	**Z80**	1/1	0.008	Family history of primary malignant neoplasm

Table A.20: T(ALL2ALL)-E(ALL2ALL) model: Statements with AUROCs smaller than 0.7 (Part 3). The statements are sorted by chapter and prevalence in the dataset. Coverage refers to the fraction of codes within this 3-digit category that stay below the specified threshold of 0.7. Categories in boldface correspond to categories with a coverage of 75% or higher, i.e., a situation where the category and 75% of the subcategories are covered with AUROC scores below 0.7.
